# SlSLAH2 mediates malate exudation and contributes to aluminum tolerance

**DOI:** 10.1038/s41467-026-71651-1

**Published:** 2026-04-10

**Authors:** Danhui Dong, Congyang Jia, Jialong Zhang, Yiran Wang, Ming Gao, Junxin Guo, Chengcheng Shen, Zhirong Wang, Lei Zhang, Tao Lin, Jie Ye, Na Zhang, Yang-Dong Guo

**Affiliations:** 1 College of Horticulture, State Key Laboratory of Efficient Utilization of Agricultural Water Resources, China Agricultural University, Beijing, China; 2 National Key Facility for Crop Gene Resources and Genetic Improvement/Key Laboratory Grain Crop Genetic Resources Evaluation and Utilization, Ministry of Agriculture and Rural Affairs, Institute of Crop Sciences, Chinese Academy of Agricultural Sciences, Beijing, China; 3 Institute of Vegetables and Flowers, Chinese Academy of Agricultural Sciences, Beijing, China; 4 College of Horticulture and Forestry Sciences, Huazhong Agricultural University, Wuhan, China

**Keywords:** Abiotic, Plant molecular biology, Plant signalling

## Abstract

Malate transporters play pivotal roles in plant aluminum tolerance mechanisms. In the classic aluminum tolerance pathway, Al^3+^ induces *ALMT*, which promotes malate exudation to chelate Al^3+^ to enhance aluminum tolerance. However, in tomato, *SlALMT* was inhibited by Al^3+^, but Al^3+^ still induced malate exudation. We found that SlSLAH2, upon induction by Al^3+^, can transport malate and is required for full activation of malate exudation by Al^3+^ stress. SlWRKY37 contributes to *SlSLAH2* induction by Al^3+^. Moreover, SlSLAH2 is phosphorylated in response to Al^3+^. We identify SlCDPK21 and SlPP2C72 as putative upstream kinase and phosphatase that could potentially facilitate phosphorylation homeostasis. SlCDPK21 can interact with SlSLAH2 in a heterologous system, phosphorylate SlSLAH2 at Thr167 in vitro and is also required for full malate exudation. SlPP2C72 can dephosphorylate SlSLAH2 in vitro and knock-out leads to increased malate exudation. Furthermore, Al^3+^ downregulated *SlPP2C72*, and Al^3+^ treated seedling extracts can suppress SlPP2C72 phosphatase activity. We propose a synergistic transcription-phosphorylation cascade that can ensure a robust malate exudation across Al^3+^ environments.

## Abstract (web summary)

This study identifies a tomato Al tolerance pathway: SlSLAH2 mediates malate exudation, transcriptionally activated by SlWRKY37, phosphorylated by SlCDPK21, and repressed by SlPP2C72, to chelate toxic Al.

## Introduction

Aluminum (Al), the most abundant metal element in the Earth’s crust, predominantly occurs in the form of Al oxides or insoluble aluminosilicates when the soil pH is neutral or alkaline. These forms are non-toxic to plants. However, when the soil pH drops below 5.5, Al is transformed into soluble free Al^3+^, which binds to plant root tips and inhibits the absorption of water and nutrients, thereby inducing Al toxicity in plants. Given that approximately 40% of the world’s arable land is acidic soil, Al toxicity is considered the primary factor limiting plant growth in such environments. Al at the micro molar concentration level can cause severe toxicity to plant roots. Consequently, Al stress has emerged as the second most severe abiotic stress after drought stress^1,2^. Although Al causes severe damage to most plants, some species exhibit Al tolerance. For instance, low concentrations of Al have been shown to promote the growth of tea plants^3,4^.

Plants have evolved intricate mechanisms to combat Al toxicity, encompassing both external exclusion and internal tolerance strategies. External exclusion mechanisms mitigate Al toxicity by preventing Al^3+^ from entering root cells, while internal tolerance mechanisms involve sequestering Al^3+^ that have entered the cells into vacuoles or chelating them within the cell, thereby alleviating Al toxicity. The exudation of organic acid (malate, citrate, or oxalate) by roots under Al stress, which chelate Al^3+^ in the rhizosphere and prevent their uptake by plant cells, is the most widely studied and recognized Al resistance mechanism^5–7^. Usually, the transport of organic acid is mediated by transporters or anion channels located in the plasma membrane. For instance, *TaALMT1* (*Aluminum-activated Malate Transporter*) in wheat encoded an aluminum-activated malate transporter that increased the exudation of malate under Al stress, enhancing wheat’s Al tolerance^8^. Subsequently, homologs of *TaALMT1* have been identified in various species, such as *AtALMT1*^9^, *MsALMT1*^10^, and *GmALMT1*^11^, all of which improved plant Al tolerance by promoting the exudation of malate. Among them, the mechanism of AtALMT1 in Al^3+^-induced malate exudation has been the most extensively studied. Al stress enhanced the exudation of malate by AtALMT1 through regulation at both the protein and transcriptional levels. AtALMT1 underwent precise post-translational modifications, presumably mediated by protein kinases and phosphatases^12^. The transcription level of *AtALMT1* was upregulated under Al stress by AtSTOP1^13^ and AtWRKY46^14^. Subsequent studies have revealed that, in addition to its role in Al stress, ALMT also enhances fluoride tolerance, responds to drought stress, and participates in plant growth and development^15–17^.

The slow-type anion channel (also referred to as SLAC/SLAHs) was first found in *Vicia faba* guard cells by the patch-clamp electrophysiology^18,19^. SLAC/SLAHs exhibit slow activation/inactivation kinetics and weak voltage dependence in the 10-s range. It has a high selectivity for nitrate, but has a lesser degree of permeability to chloride and malate^20^. The SLAC1 protein has been identified as the main component of the slow-anion current^21,22^. The *SLAC1* gene codes for a transmembrane protein with ten transmembrane α-helices. The N-terminal (NT) and C-terminal (CT) regions of SLAC1 form cytoplasmic regulatory domains (CRDs) that interact with the transmembrane domain (TMD) to maintain the auto-inhibited state of SLAC1 at rest. During SLAC1 activation, phosphorylation modifications induce conformational changes in the CRD and relieve auto-inhibition^23^. The regulation of anion channel activity involves the phosphorylation dynamics as well as cytoplasmic calcium signaling^24,25^. Intriguingly, alternative activation pathways exist, as demonstrated by SLAH3 gating through pH-dependent histidine protonation independent of kinase signaling^26^. Functional studies have established SLAC/SLAHs involvement in diverse physiological processes, including stomatal regulation^22,27^, alleviation of ammonium toxicity^28–30^, flooding stress^26^, pollen tube growth^31^, nitrogen-potassium homeostasis^32^, salt stress^33^ by mediating the transport of Cl^−^ and NO_3_^−^. Although it has been demonstrated that the SLAC/SLAHs family possesses malate permeability^34^, current evidence only specifically links malate transport through SLAC/SLAHs to stomatal closure mechanisms^35^. It is necessary to explore more physiological processes in which the SLAC/SLAHs family is involved in malate transport.

Previous work demonstrated that Al^3+^-induced malate exudation in tomato varieties was associated with SlALMT9, a tonoplast-localized malate transporter^36^. In this study, we found that in tomato, external Al^3+^ inhibited the expression of *SlALMT* genes, yet still triggered pronounced malate exudation. Thus, we hypothesized that in tomato, there was an Al^3+^-activated malate release independent of *SlALMT*. We now present evidence that the slow-type anion channel SlSLAH2 orchestrated this alternative exudation. Al^3+^ initiated a multilayered signaling cascade that converged on SlSLAH2. Al^3+^ triggered the SlWRKY37 to active the transcription of *SlSLAH2*. Meanwhile, Al^3+^ also activated the Ca^2+^-dependent kinase SlCDPK21, which phosphorylated SlSLAH2 at Thr167 (T167), thereby enhancing channel open probability and malate conductance. To sustain this phosphorylation, Al^3+^ downregulated the phosphatase SlPP2C72 both transcriptionally and post-translationally. SlCDPK21-mediated phosphorylation of SlPP2C72 reduced its phosphatase activity, forming a double-check that prevented dephosphorylation of SlSLAH2. Collectively, our research reveals a divergent malate transporter choice for Al detoxification strategies and establishes the molecular framework underlying organic acid exudation in tomato.

## Results

### In tomato, Al^3+^ induced transcriptional upregulation of *SlSLAC*/*SLAHs* but not *SlALMT*

**Fig. 1:**
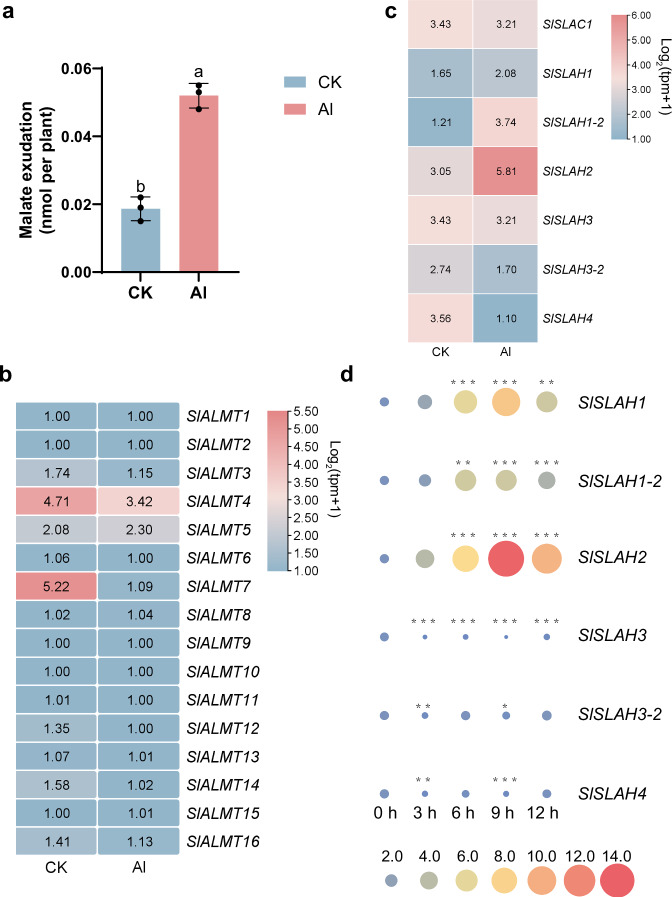
Al^3+^ induced expressions of *SlSLAC*/*SLAHs* instead of *SlALMT* in tomato. **a** Malate exudation in roots of MicroTom WT. One-month-old tomato plants were treated with 0.5 mM CaCl_2_, 90 μM AlCl_3_ (pH 4.7) for 12 h. Data were presented as means ± SD (*n* = 3). Statistical significance was analyzed by unpaired two-tailed *t* test; different lowercase letters indicated significantly different means (*p* ≤ 0.05). Heatmap analysis of (**b**) *SlALMT* and (**c**) *SlSLAC*/*SLAHs* gene expression in MicroTom WT treated with or without 60 μM AlCl_3_ for 9 h (pH 4.7). Expression values were shown as log_2_^(tpm+1)^ (*n* = 3). **d** The expression levels of *SlSLAC*/*SLAHs* were detected by RT-qPCR in 14-day-old MicroTom under 60 μM AlCl_3_ (pH 4.7) treatment in 12 h. *SlUBI* was used as reference gene. Bubble size and color lightness corresponded to relative expression values, with larger/lighter bubbles indicating higher expression. Data were presented as means (*n* = 3). Statistical significance was analyzed by one-way ANOVA (Dunnett’s multiple comparisons test, *p ≤ 0.05; **p ≤ 0.01, ***p ≤ 0.001).

ALMT-type transporters are generally regarded as Al^3+^-activated malate transporters. Although previous studies showed Al^3+^ triggered malate exudation in tomato^36^, but we didn’t find the specific plasma membrane-localized SlALMT responsible for this process^37^. Here, we first confirmed that Al^3+^ upregulated the malate release in tomato root (Fig. 1a). To identify the responsible SlALMT, we analyzed RNA-seq data from Al^3+^ treated MicroTom roots^37^. All *SlALMT* genes were transcriptionally repressed rather than induced by Al^3+^ treatment (Fig. 1b), which was confirmed by RT-qPCR analysis (Fig. S1a). To exclude cultivar specificity, we checked root *SlALMT* expression in several additional varieties and observed the same Al^3+^-dependent down-regulation in other varieties (Figs. S1b and S2). This observation led us to investigate whether tomato possessed alternative malate transporters that functionally compensate for the repressed *SlALMT*.

As the SLAC/SLAHs family has been reported for malate permeability, we re-examined the RNA-seq data and identified three *SlSLAC*/*SLAHs* members that responded to Al stress, with *SlSLAH2* showing the strongest induction (Fig. 1c). Time-course RT-qPCR of Al^3+^-treated roots revealed rapid and robust activation of *SlSLAH2*, *SlSLAH1*-*2*, and *SlSLAH1*, with *SlSLAH2* responding more strongly (Figs. 1d and S2). Thus, unlike Arabidopsis and soybean where ALMT proteins function as malate transporters due to their transcriptional response to Al stress^9,11^, tomato SlSLAC/SLAHs proteins exhibited transcriptional upregulation under Al stress and represented potential candidates for malate exudation carriers.

### SlSLAH2, localized to the plasma membrane, mediated malate transport under Al stress

**Fig. 2:**
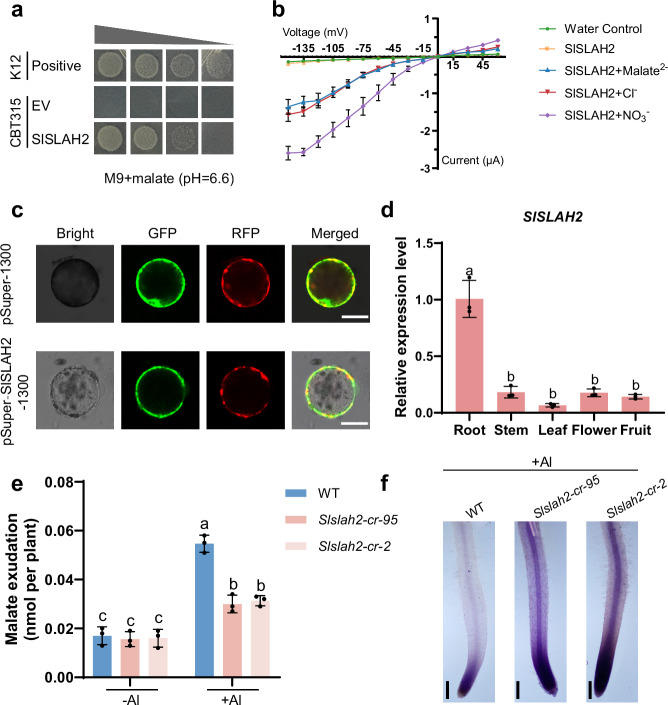
SlSLAH2 was a plasma membrane-localized malate transporter. **a** SlSLAH2 partly restored the growth defect of dicarboxylate-uptake-deficient *E. coli* mutant CBT315. The SlSLAH2-pKK223-3 vector and pKK223-3 empty vector (EV) was transferred into CBT315, respectively. WT (K12) transfected with the EV was set as positive control. The positive transformed monoclonal plaque was cultured on M9 agar medium with 10 mM malate (pH 6.6) as the sole carbon source for 3 days. **b** SlSLAH2 was an anion channel. Average steady-state current–voltage (I–V) curves of SlSLAH2 anion channels recorded in the indicated external solutions. The number of oocytes tested was 3 (water control and SlSLAH2) and 5 (SlSLAH2 + 50 mM Malate^2−^, SlSLAH2 + 50 mM Cl^−^, SlSLAH2 + 50 mM NO_3_^−^). Error bars indicate means ± SE. **c** Subcellular location of SlSLAH2. pSuper-SlSLAH2-1300 was transiently expressed in *N. benthamiana* leaves. RFP was used to visualize plasma membrane, PMH (Plasma Membrane-Localized H^+^-ATPase) was set as plasma membrane marker. pSuper-1300 was set as a mock control. Scale bar, 50 μm. **d** Tissue-specific expression analysis of *SlSLAH2* in tomato. *SlUBI* was used as reference gene. Data were presented as means ± SD (*n* = 3). Statistical significance was analyzed by one-way ANOVA, different lowercase letters indicated significantly different means (Tukey’s multiple comparisons test, *p* ≤ 0.05). **e** Malate exudation in roots of MicroTom WT lines and *Slslah2* mutant lines. One-month-old tomato plants were treated with 0.5 mM CaCl_2_, 90 μM AlCl_3_ (pH 4.7) for 12 h. Data were presented as means ± SD (*n* = 3). Statistical significance was analyzed by two-way ANOVA, different lowercase letters indicated significantly different means (Tukey’s multiple comparisons test, *p* ≤ 0.05). **f** Hematoxylin staining of root Al content. Roots of 14-day-old WT and *Slslah2* mutants treated with 90 μM Al^3+^ were stained with hematoxylin and subsequently washed with ultra-pure water. Scale bar, 100 μm.

To verify SlSLAH2’s malate transport activity, we expressed SlSLAH2 in the dicarboxylate-uptake-deficient *E. coli* strain CBT315, which was unable to utilize malate as carbon source^38^. Expression of SlSLAH2 restored bacterial growth on malate-containing medium, confirming its malate transport capability (Fig. 2a). Electrophysiological analysis of *Xenopus* oocytes transiently expressing SlSLAH2 in a bath solution containing 50 mM malate^2−^ revealed that malate^2^^−^ activated SlSLAH2, indicating that it functioned as a malate-transporting ion channel (Figs. 2b and  S3a). Further functional characterization demonstrated that SlSLAH2 was a multifunctional anion channel capable of transporting nitrate and chloride, as evidenced by voltage-clamp recordings conducted in solutions containing the respective anions (Figs. 2b and S3a). Subcellular localization confirmed its plasma membrane localization (Figs. 2c and S3b). Multiple sequence alignment between Arabidopsis AtSLAC1 and SlSLAH2 revealed high conservation in their transmembrane domains (Fig. S4), and three conserved phenylalanine residues that are essential for SLAC/SLAH channel activation were strictly conserved in SlSLAH2^39,40^. Alphafold3-based structure prediction resolved SlSLAH2 as a ten-transmembrane-helix channel protein, consistent with its role as an anion transporter (Fig. S5). Tissue-specific expression analysis showed *SlSLAH2* was specifically highly expressed in roots (Fig. 2d), further supporting its role in Al^3+^-induced malate exudation.

To determine the *in planta* contribution of SlSLAH2 to Al^3+^-activated malate exudation, we used knockout lines of *SlSLAH2* by CRISPR/Cas9 in MicroTom background. Two independent lines, *Slslah2*-*cr*-*2* (2 bp deletion) and *Slslah2*-*cr*-*95* (95 bp deletion)^41^, both introduced premature stop codons (Fig. S6). When exposed to Al^3+^ treatment for 12 h, two mutant lines showed a about 40% reduction in root malate exudation compared to WT (Fig. 2e), confirming that SlSLAH2 mediated malate transport under Al stress. Enhanced Al accumulation was observed in the roots of the *Slslah2* mutant by hematoxylin staining, demonstrating compromised Al tolerance (Fig. 2f).

In summary, our results demonstrated that the plasma membrane transporter SlSLAH2 contributed to the Al^3+^-induced malate exudation in tomato.

### SlWRKY37 upregulated *SlSLAH2* expression to enhance malate exudation under Al stress

To explore how *SlSLAH2* responds to Al stress transcriptionally, we first examined whether the canonical Al-tolerance regulator SlSTOP1 was involved. In the *Slstop1* mutant^37^, Al^3+^-induced *SlSLAH2* expression was indistinguishable from WT (Fig. S7a, b). Dual-luciferase LUC (Firefly Luciferase)/REN (Renilla Luciferase) assays with two segments of the 2000 bp *SlSLAH2* promoter confirmed that SlSTOP1 failed to trans-activate either segment (Fig. S7c), indicating that SlSTOP1 was not a direct upstream regulatory factor of *SlSLAH2*.

**Fig. 3:**
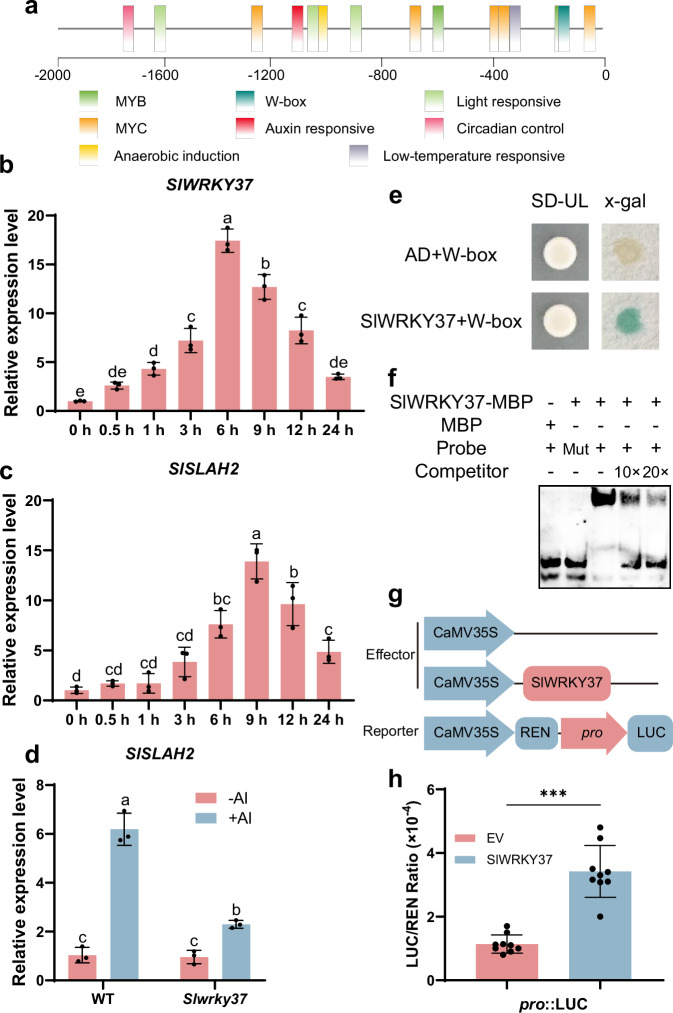
SlWRKY37 regulated *SlSLAH2* expression under Al stress. **a** Promoter analysis of *SlSLAH2*. **b**,** c** The expression levels of *SlWRKY37* and *SlSLAH2* in 14-day-old WT treated with 60 μM AlCl_3_ (pH 4.7) over time were detected by RT-qPCR. *SlUBI* was used as reference gene. Data were presented as means ± SD (*n* = 3). Statistical significance was analyzed by one-way ANOVA, different lowercase letters indicated significantly different means (Tukey’s multiple comparisons test, *p* ≤ 0.05). **d** The expression level of *SlSLAH2* in 14-day-old WT and *Slwrky37* mutant lines treated with or without 60 μM AlCl_3_ (pH 4.7) for 9 h was detected by RT-qPCR. *SlUBI* was used as reference gene. Data were presented as means ± SD (*n* = 3). Statistical significance was analyzed by two-way ANOVA, different lowercase letters indicated significantly different means (Tukey’s multiple comparisons test, *p* ≤ 0.05). **e** Y1H assay. The *SLAH2* promoter fragment containing W-box was cloned into pLacZi vector, *SlWRKY37* was cloned into pGAD424 vector. The transformants pGAD424 and fragment were set as negative control. Transformants with paired constructs were grown on SD-UL medium and then used for binding assay in Z-buffer with X-gal. **f** EMSA assay. Unlabeled probe was set as competitive probe. Mut indicated mutated competitive probe. **g**, **h** LUC/REN assay. *SlWRKY37* was cloned into pGreen II 62-SK. The fragment of *SlSLAH2* was fused with the LUC as reporter. Empty vector (pGreen II 62-SK) co-expressing with reporter was set as the control. Data were presented as means ± SD (*n* = 9). Statistical significance was analyzed by paired two-tailed *t* test (***p ≤ 0.001).

We therefore analyzed the 2000 bp promoter of *SlSLAH2* using the PlantCARE (https://bioinformatics.psb.ugent.be/webtools/plantcare/html/), and identified a canonical W-box (WRKY-binding) motif, together with predicted light and auxin response elements (Fig. 3a). Our previous study showed that the transcription level of *SlWRKY37* was significantly increased after Al^3+^ treatment^42^, and SlWRKY37 acted as a transcriptional activator binding to W-box elements^43^. Time-course RT-qPCR revealed that the expression levels of *SlWRKY37* increased significantly after 1-h Al^3+^ treatment (Fig. 3b), preceding the 6-h induction of *SlSLAH2* (Fig. 3c). Both *SlWRKY37* and *SlSLAH2* were expressed in the root tips (Fig. S8). Consistently, in the *Slwrky37* mutant lines, the Al^3+^-induced expression of *SlSLAH2* was significantly suppressed (Fig. 3d). This evidence all indicated that SlWRKY37 might be an upstream activator of *SlSLAH2* under Al stress.

We then performed a yeast one-hybrid (Y1H) assay by inserting the W-box into the pLacZi vector and co-transforming it with SlWRKY37-pGAD424 into the YM4271 yeast strain. The results showed cells carrying both the SlWRKY37 activator and a reporter gene driven by the W-box turned blue, indicating direct binding (Fig. 3e). EMSA (Electrophoretic Mobility Shift Assay) further confirmed this result, demonstrating that SlWRKY37 could not bind when the W-box was mutated (Fig. 3f). LUC/REN assays indicated that SlWRKY37 activated reporter gene expression driven by the promoter containing the W-box (Fig. 3g, h). In conclusion, our results demonstrated that SlWRKY37 functioned as the upstream transcription factor of *SlSLAH2* and activated its expression under Al stress.

**Fig. 4:**
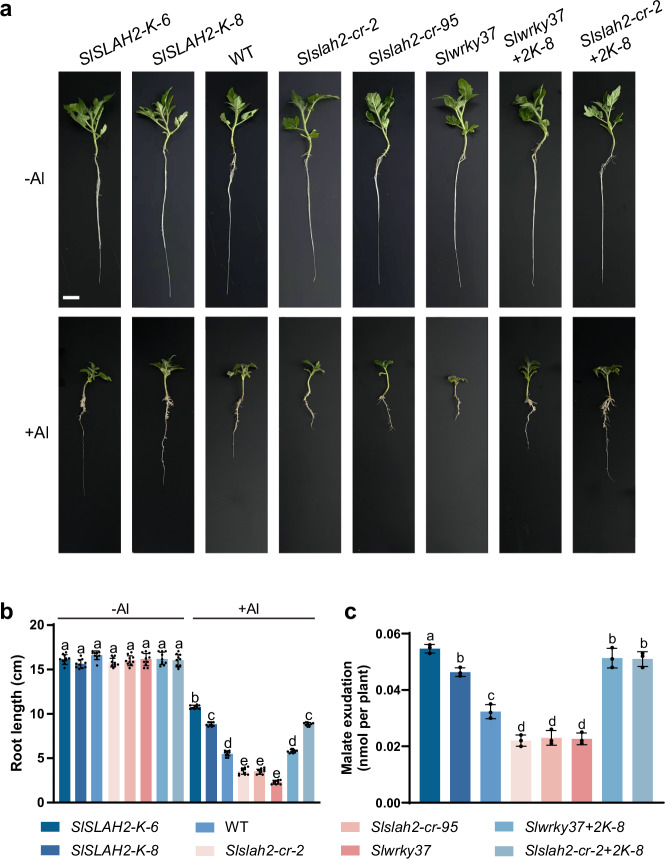
SlSLAH2 enhanced the Al tolerance by increasing the exudation of malate in tomato. **a** Root growth of WT, *SlSLAH2* overexpression lines and knockout lines under normal condition and Al stress. 7-day-old tomato plants were grown in modified Hoagland medium supplemented with 0 or 30 μM AlCl_3_ (pH 4.5) for 14 d. Scale bar, 2 cm. **b** Root length of different lines in (**a**). Root length was shown without or with Al^3+^ treatment for 14 days. Data were presented as means ± SD (*n *= 10). Statistical significance was analyzed by one-way ANOVA, different lowercase letters indicated significantly different means (Tukey’s multiple comparisons test, *p* ≤ 0.05). **c** Malate exudation of different lines. One-month-old tomato plants treated for 12 hours with 0.5 mM CaCl_2_, 90 μM AlCl_3_ (pH 4.7) was used for malate exudation. Data were presented as means ± SD (*n* = 3). Statistical significance was analyzed by one-way ANOVA, different lowercase letters indicated significantly different means (Tukey’s multiple comparisons test, *p* ≤ 0.05).

We evaluated the physiological role of the SlWRKY37-*SlSLAH2* module by exposing tomato seedlings to Al^3+^ treatment for 14 days. Under normal conditions, root elongation was identical across WT, knockout and overexpression lines. However, Al^3+^ treatment shortened the roots of *Slslah2* and *Slwrky37* mutants far more than those of WT plants, while lines overexpressing *SlSLAH2* showed significant longer roots (Fig. 4a, b). Complementation of *SlSLAH2* in the *Slwrky37* mutant lines partially restored the root length (Fig. 4a, b), suggesting that the two genes functioned along the same pathway. After 12-h Al^3+^ treatment, both *Slslah2* and *Slwrky37* mutant lines showed reduced malate exudation compared to WT, which was largely recovered by *SlSLAH2* complementation (Fig. 4c).

Collectively, these findings suggested that SlSLAH2 conferred Al tolerance by mediating malate exudation, and this process was regulated by SlWRKY37.

### SlCDPK21 directly interacted with and phosphorylated SlSLAH2

**Fig. 5:**
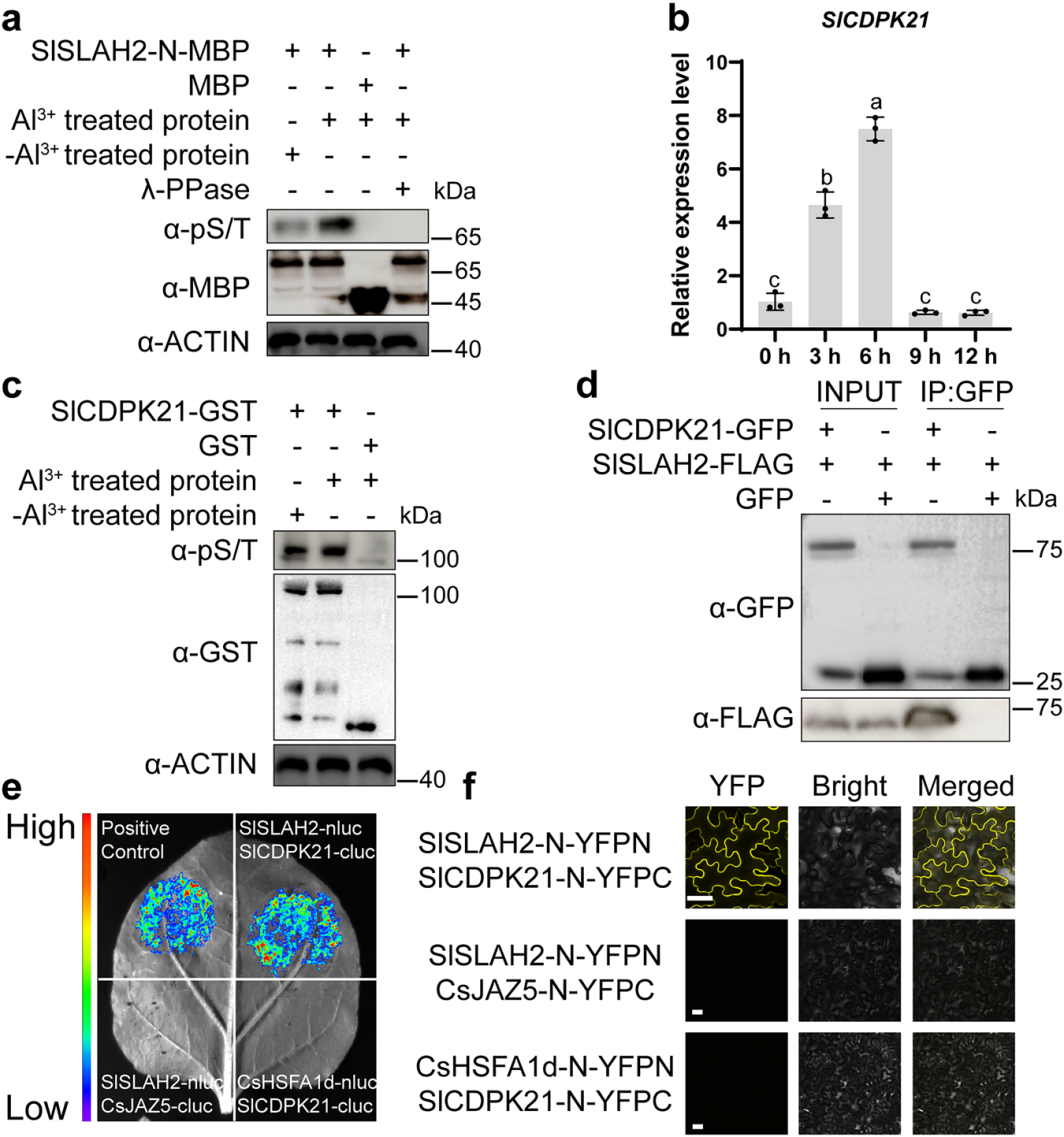
SlCDPK21 directly interacted with SlSLAH2. **a** Phosphorylation of the N terminus of SlSLAH2 induced by 0 or 90 μM AlCl_3_. Total proteins were extracted from 0 or 90 μM AlCl_3_ treated tomato roots of WT. The samples derived from the same experiment and that blots were processed in parallel. **b** The expression level of *SlCDPK21* in WT treated with 60 μM AlCl_3_ (pH 4.7) in different time points were detected by RT-qPCR. *SlUBI* was used as reference gene. Data were presented as means ± SD (*n* = 3). Statistical significance was analyzed by one-way ANOVA, different lowercase letters indicate significantly different means (Tukey’s multiple comparisons test, *p* ≤ 0.05). **c** Phosphorylation of SlCDPK21 induced by 0 or 90 μM AlCl_3_. Total proteins were extracted from 0 or 90 μM AlCl_3_ treated tomato roots of WT. The samples derived from the same experiment and that blots were processed in parallel. **d** Co-IP assay showed SlCDPK21 interacted with SlSLAH2. SlSLAH2-FLAG and SlCDPK21-GFP were transiently co-transfected into *N. benthamiana* leaves. **e** LCI assay showed the interaction between SlCDPK21 and SlSLAH2. Constructs carrying SlCDPK21-cluc and SlSLAH2-nluc were co-expressed in *N. benthamiana* leaves for 3 d. CsHSFA1d-nluc and CsJAZ5-cluc were used as positive control. **f** BiFC assay showed that the interaction between SlCDPK21 and SlSLAH2 occurred at the plasma membrane. SlCDPK21-N-YFPC and SlSLAH2-N-YFPN proteins were transiently co-expressed in *N. benthamiana*. Scale bar, 50 μm. Experiments in (**a**, **c**–**f**) were independently repeated three times with similar results.

Studies have shown that SLAC/SLAHs family proteins undergo post-translational regulation by kinases and phosphatases, including calcium-dependent protein kinases (CDPKs/CPKs) and type 2 C protein phosphatases (PP2Cs), to modulate channel activity under various conditions^29,31,44–46^. To investigate whether SlSLAH2 was activated through phosphorylation during Al stress to facilitate malate exudation, we performed phosphorylation assays to confirm SlSLAH2 undergo Al^3+^-induced phosphorylation. The phosphorylation level of SlSLAH2 increased with rising Al^3+^ concentrations (Fig. S9a). Recombinant SlSLAH2-N-terminal protein fused with MBP tag was co-incubated with Al^3+^-treated root extracts, resulting in significantly enhanced phosphorylation, which was abolished by λ-PPase treatment (Figs. 5a and S9b). These results provided biochemical evidence that phosphorylation of SlSLAH2 was regulated by Al^3+^-inducible kinase pathways.

Al stress is known to alter cytosolic Ca^2+^ activity^47–49^, potentially contributing to Al toxicity. Under Al stress, Al^3+^-induced Ca^2+^ burst promoted the exudation of malate by increasing the expression of *AtALMT1*^48^. We hypothesized that CDPKs, as direct Ca^2+^ sensors, might perceive Al^3+^-induced cytosolic Ca^2+^ increase and transduced Al toxicity signals to SlSLAH2 through phosphorylation, ultimately enhancing Al tolerance in tomato. To verify this hypothesis, we identified potential SlCDPK candidates interacting with SlSLAH2 through tomato multi-omics data analysis platform (https://bioinformatics.cau.edu.cn/TomAP/index.html). Although, RNA-seq data of Al^3+^ treatment exhibited no significant change of *SlCDPK21* expression, other *SlCDPKs* exhibited decreased expressions after Al^3+^ treatment (Fig. S10a). Phylogenetic analysis revealed that SlCDPK21 was closely homologous to AtCPK21 and AtCPK23 (Fig. S10b), which interacted with and phosphorylated AtSLAC1^44^. Time-course RT-qPCR result showed that *SlCDPK21* responded Al^3+^ treatment at earlier time points (3 h and 6 h) than RNA-seq sample time (9 h), confirming its transcriptional response to Al stress (Fig. 5b). Tissue-specific expression profiles revealed that *SlCDPK21* exhibited constitutively high expression levels across all tissues^50^ and showed higher expression at root tips (Fig. S10c, S10d), while other *SlCDPKs* showed lower expression in root. CDPK proteins typically undergo autophosphorylation, a modification that enables signal transduction. To verify Al^3+^-induced phosphorylation of SlCDPK21, we co-incubated SlCDPK21-GST with Al^3+^-treated root protein. Results demonstrated that Al^3+^ promoted SlCDPK21 phosphorylation compared to non-Al^3+^ treated protein (Fig. 5c). These findings demonstrated that SlCDPK21 was activated both transcriptionally and post-transcriptionally by Al^3+^ treatment, suggesting it may be the kinase responsible for SlSLAH2 phosphorylation in response to Al stress.

We hypothesized that SlCDPK21 might mediate Al toxicity signals by directly targeting SlSLAH2, as CDPK was known to phosphorylate SLAC1 both in vitro and in vivo^44^. We confirmed a direct interaction between SlCDPK21 and SlSLAH2 through Co-IP (Co-Immunoprecipitation) and LCI (Split-Luciferase) assay (Fig. 5d, e). SlSLAH2-FLAG co-immunoprecipitated with GFP-tagged SlCDPK21, and their co-expression produced strong luciferase signals. BiFC (Bimolecular Fluorescence Complementation) further localized this interaction to the plasma membrane (Fig. 5f).

**Fig. 6:**
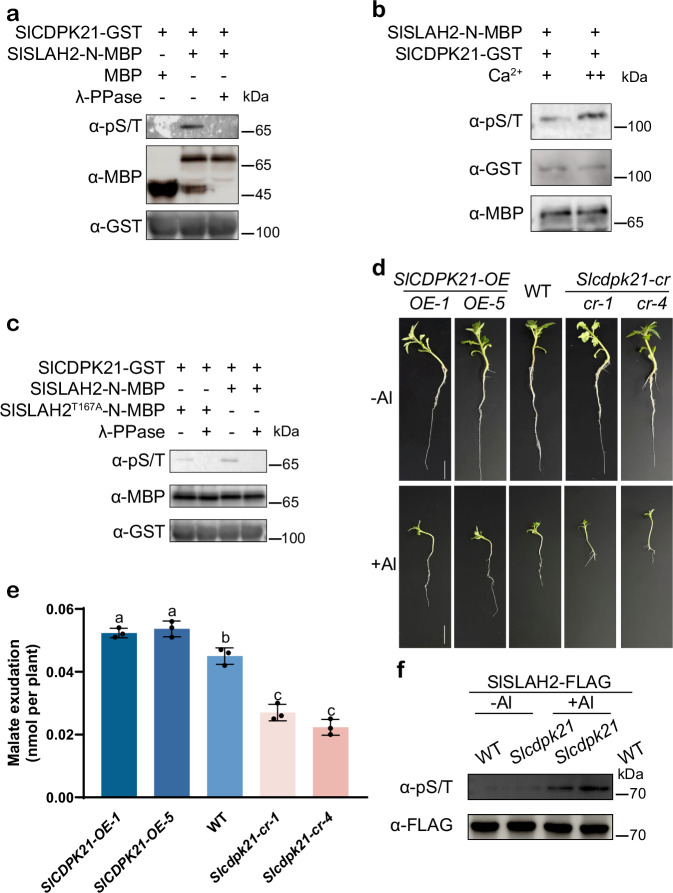
SlCDPK21 enhanced Al tolerance in tomato by increasing phosphorylation of SlSLAH2. **a** In vitro kinase assay showed SlCDPK21 phosphorylated SlSLAH2. Recombinant SlSLAH2 and SlCDPK21 proteins were incubated in protein kinase buffer supplemented with  ATP. **b** In vitro kinase assay showed the phosphorylation of SlSLAH2 by SlCDPK21 was calcium-dependent. Recombinant SlSLAH2 and SlCDPK21 proteins were incubated in protein kinase buffer supplemented with  ATP and either 1 mM or 5 mM CaCl_2_. **c** In vitro kinase assay showed T167 of SlSLAH2 was a target of SlCDPK21. Recombinant SlSLAH2, SlSLAH2^T167A^ (mutant form with the T167 replaced with A167) and SlCDPK21 proteins were incubated in protein kinase buffer supplemented with  ATP. **d** Root growth of WT, *SlCDPK21* overexpression lines and knockout lines under normal condition and aluminum stress. 7-day-old tomato plants were grown in modified Hoagland medium supplemented with 0 or 30 μM AlCl_3_ (pH 4.5) for 14 days. Scale bar, 2 cm. **e** Malate exudation of different lines. One-month-old tomato plants were treated with 0.5 mM CaCl_2_, 90 μM AlCl_3_ (pH 4.7) for 12 h. Data were presented as means ± SD (*n* = 3). Statistical significance was analyzed by one-way ANOVA, different lowercase letters indicated significantly different means (Tukey’s multiple comparisons test, *p* ≤ 0.05). **f** Phosphorylation levels of SlSLAH2 in WT and *Slcdpk21*. SlSLAH2 protein was co-incubated with WT or *Slcdpk21* protein treated with 0 or 90 μM AlCl_3_ for 3 h. Experiments in (**a**–**f**) were independently repeated three times with similar results.

We tested SlCDPK21’s ability to phosphorylate SlSLAH2 using an α-pS/T antibody. The results showed that the recombinant N-terminal SlSLAH2 fragment (SlSLAH2-N-MBP) was strongly phosphorylated by SlCDPK21 in vitro compared to a MBP control (Fig. 6a), and the phosphorylation signal increased upon CaCl_2_ addition, confirming calcium dependence (Fig. 6b). Therefore, we concluded that SlCDPK21 phosphorylated SlSLAH2 in a calcium-dependent manner. Mass spectrometry^51^ identified Thr167 (T167) as a phosphorylation site in SlSLAH2’s N-terminal region, which was conserved with AtSLAH3 Thr187 (T187) and highly conserved in SLAH across different plant species (Fig. S11). To verify T167 as a target of SlCDPK21, we conducted in vitro phosphorylation assays using SlSLAH2-N-MBP and SlSLAH2^T167A^-N-MBP, in which the T167 residue was replaced with Ala167 (A167) to prevent phosphorylation. The SlSLAH2^T167A^-N-MBP showed reduced phosphorylation level in the presence of SlCDPK21 (Figs. 6c and S13a), indicating that T167 was a major, though not exclusive, phosphorylation site for SlCDPK21 on SlSLAH2.

To investigate whether SlCDPK21 was involved in regulating Al tolerance, we generated *Slcdpk21* mutants using the CRISPR/Cas9 gene editing system (Fig. S12). After Al^3+^ treatment, root elongation was significantly inhibited in the *Slcdpk21* mutant lines, whereas *SlCDPK21* overexpressing lines exhibited longer roots compared with the WT, indicating that SlCDPK21 enhanced Al tolerance in tomato (Fig. 6d). Further short-term Al^3+^ treatment experiments revealed that knockout of *SlCDPK21* markedly reduced Al^3+^-induced malate exudation (Fig. 6e). These results support the conclusion that SlCDPK21 contributed to tomato Al tolerance by regulating malate exudation. To examine whether SlCDPK21 mediated malate exudation via phosphorylation of SlSLAH2, we analyzed the phosphorylation status of SlSLAH2. Co-incubation of SlSLAH2 protein with protein extracts from WT and *Slcdpk21* mutants treated with or without Al^3+^ revealed a significant increase in the phosphorylation level of SlSLAH2 in the WT, while the phosphorylation level was reduced in the *Slcdpk21* (Figs. 6f and S13b). Taken together, our findings indicated that phosphorylation of SlSLAH2 by SlCDPK21 acted as a mechanism for transducing Al toxicity signals, ultimately to promote malate exudation.

### SlSLAH2 was dephosphorylated by SlPP2C72

**Fig. 7:**
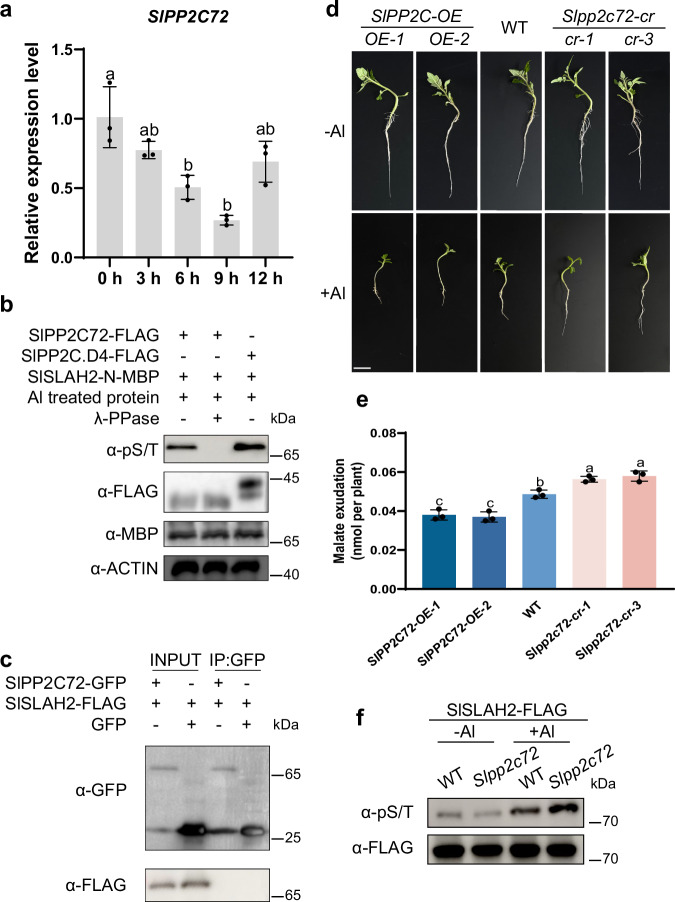
SlSLAH2 was indirectly dephosphorylated by SlPP2C72. **a** The expression level of *SlPP2C72* in WT treated with 60 μM AlCl_3_ (pH 4.7) over time was detected by RT-qPCR. *SlUBI* was used as reference gene. Data were presented as means ± SD (*n* = 3). Statistical significance was analyzed by one-way ANOVA, different lowercase letters indicated significantly different means (Tukey’s multiple comparisons test, *p* ≤ 0.05). **b** SlPP2C72 dephosphorylated SlSLAH2 in vitro. Recombinant SlSLAH2 was co-incubated with 90 μM AlCl_3_ treated tomato root extracted proteins. Then SlPP2C72 or SlPP2C.D4 proteins were added to this system. The samples derive from the same experiment and that blots were processed in parallel. **c** Co-IP assay showed SlSLAH2 did not interact with SlPP2C72. SlSLAH2-FLAG and SlPP2C72-GFP were transiently co-transfected into *N. benthamiana* leaves, and α-GFP affinity magnetic beads were used for immunoprecipitation. **d** Root growth of WT, *SlPP2C72* overexpression lines and knockout lines under normal condition and aluminum stress. 7-day-old tomato plants were grown in modified Hoagland medium supplemented with 0 or 30 μM AlCl_3_ (pH 4.5) for 14 d. Scale bar, 2 cm. **e** Malate exudation of different lines. One-month-old tomato plants treated with 0.5 mM CaCl_2_, 90 μM AlCl_3_ (pH 4.7) for 12 h. Data were presented as means ± SD (*n* = 3). Statistical significance was analyzed by one-way ANOVA, different lowercase letters indicated significantly different means (Tukey’s multiple comparisons test, *p* ≤ 0.05). **f** Phosphorylation levels of SlSLAH2 in WT and *Slpp2c72*. SlSLAH2 protein was co-incubated with WT or *Slpp2c72* treated with 0 or 90 μM AlCl_3_. Experiments in (**b**–**f**) were independently repeated three times with similar results.

SLAC1 was dynamically regulated by reversible phosphorylation to adapt to environmental changes, with PP2C-mediated dephosphorylation restoring its inactivity during stress relief to trigger stomatal reopening^23^. Previous studies demonstrated PP2C family mediated the dephosphorylation of SLAC1 or SLAH3^45,52^, therefore, we hypothesized that following Al stress alleviation, PP2C catalyzed SlSLAH2 dephosphorylation to terminate malate transport activity, thereby preventing excessive malate exudation and maintaining ionic homeostasis. By analyzing Al^3+^ treatment RNA-seq data and tomato tissue expression data^50^, we identified *SlPP2C72* (Solyc08g065670) as a candidate, which showed significant downregulation after Al stress and high root expression, especially root tips (Figs. S14a and S14b). RT-qPCR confirmed that *SlPP2C72* expression was inhibited by Al stress (Fig. 7a).

To investigate the dephosphorylation of SlSLAH2 by SlPP2C72 in vitro, we established an in vitro phosphatase system. We phosphorylated purified SlSLAH2-N-MBP protein using protein extracts from Al^3+^-treated tomato roots, then added SlPP2C72 to assess SlSLAH2 phosphorylation level. Results showed significant reduction in SlSLAH2-N-terminal phosphorylation after SlPP2C72 treatment, confirming its dephosphorylation capability (Figs. 7b and S15a). While our study confirmed that dephosphorylation of SlSLAH2 was mediated by SlPP2C72, BiFC, Co-IP assay, and LCI assay failed to detect direct physical interaction between SlSLAH2 and SlPP2C72 (Figs. 7c and S16). This suggested an indirect regulatory mechanism between SlPP2C72 and SlSLAH2.

We generated *Slpp2c72* mutants to test the phenotype (Fig. S17). After Al^3+^ treatment, root elongation was significantly inhibited in the *SlPP2C72* overexpressing lines, whereas *Slpp2c72* mutant lines exhibited longer roots compared with the WT, indicating that SlPP2C72 negatively regulated Al tolerance in tomato (Fig. 7d). Further short-term Al^3+^ treatment experiments revealed that knockout of *SlPP2C72* markedly increased Al^3+^-induced malate exudation (Fig. 7e).

To examine whether SlPP2C72 regulated malate exudation via dephosphorylation of SlSLAH2, we analyzed the phosphorylation status of SlSLAH2 in WT and *Slpp2c72* plants with or without Al^3+^ treatment. Co-incubation of SlSLAH2 protein with protein extracts from WT and *Slpp2c72* mutants treated with or without Al^3+^ revealed a significant increase in the phosphorylation level of SlSLAH2 in the *Slpp2c72* mutants, while the phosphorylation level was reduced in the WT, which indicated the dephosphorylation of SlSLAH2 by SlPP2C72 (Figs. 7f and S15b).

To verify T167 as a target of SlPP2C72, we conducted in vitro phosphorylation assays using SlSLAH2-N-MBP and SlSLAH2^T167A^-N-MBP. The SlSLAH2^T167A^-N-MBP showed reduced phosphorylation in the presence of SlPP2C72 (Fig. S18), indicating that T167 was a target for SlPP2C72.

### SlCDPK21 phosphorylated SlPP2C72 to ensure SlSLAH2 phosphorylation state

**Fig. 8:**
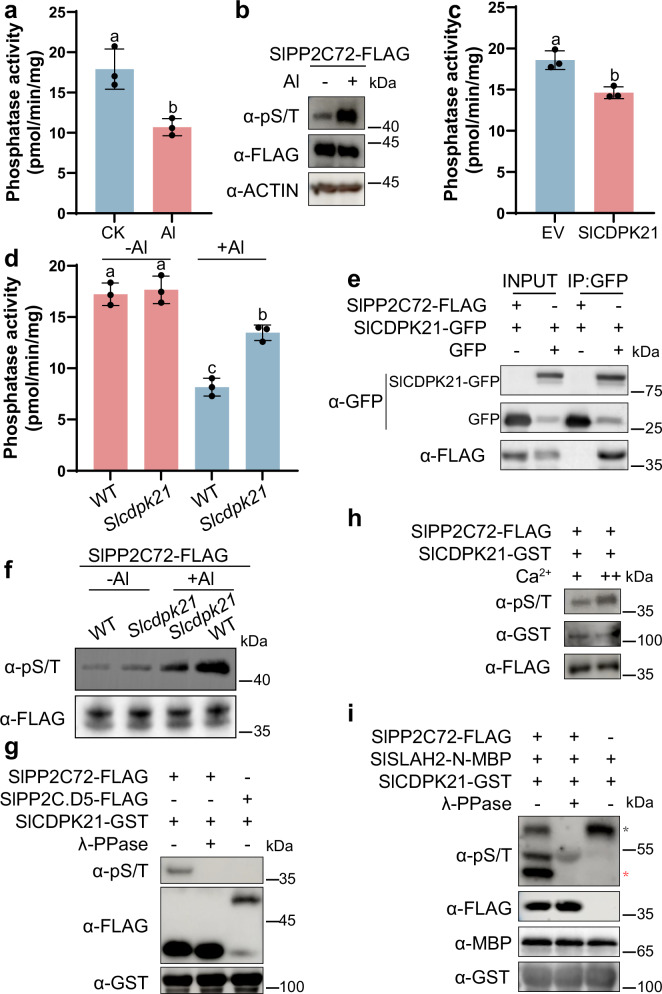
SlCDPK21 phosphorylated SlPP2C72 to ensure SlSLAH2 high phosphorylation state. **a** Effect of Al^3+^ on phosphatase activity of SlPP2C72. Total protein extracts from WT tomato plants treated with 0 or 90 μM AlCl_3_ were incubated with SlPP2C72. Data were presented as means ± SD (*n* = 3). Statistical significance was analyzed by unpaired two-tailed *t* test, different lowercase letters indicated significantly different means (*p* ≤ 0.05). **b** Phosphorylation of SlPP2C72 induced by 0 or 90 μM AlCl_3_. (samples derived from the same experiment, with blots processed in parallel). **c** Effect of SlCDPK21 on phosphatase activity of SlPP2C72. Data were presented as means ± SD (*n* = 3). Statistical significance was analyzed by unpaired two-tailed *t* test, different lowercase letters indicated significantly different means (*p* ≤ 0.05). **d** Effect of SlCDPK21 on phosphatase activity of SlPP2C72. SlPP2C72 was co-incubated with WT or *Slcdpk21* protein treated with 0 or 90 μM AlCl_3_. Data were presented as means ± SD (*n* = 3). Statistical significance was analyzed by one-way ANOVA, different lowercase letters indicated significantly different means (Tukey’s multiple comparisons test, *p* ≤ 0.05). **e** Co-IP assay showed SlCDPK21 interacted with SlPP2C72. The α-GFP affinity magnetic beads were used for immunoprecipitation. **f** Phosphorylation levels of SlPP2C72 in WT and *Slcdpk21*. SlPP2C72 was co-incubated with 0 or 90 μM AlCl_3_ treated proteins from WT or *Slcdpk21*. **g** In vitro kinase assay showed SlCDPK21 phosphorylated SlPP2C72. SlPP2C72 and SlCDPK21 proteins were incubated in protein kinase buffer supplemented with ATP. **h** In vitro kinase assay showed the phosphorylation of SlPP2C72 by SlCDPK21was calcium-dependent. SlPP2C72 and SlCDPK21 proteins were incubated in protein kinase buffer supplementing ATP with 1 mM or 5 mM CaCl_2_. **i** In vitro kinase assay showed SlCDPK21 simultaneously phosphorylated SlSLAH2 and SlPP2C72. SlPP2C72, SlSLAH2, and SlCDPK21 proteins were incubated in Al^3+^ treated proteins. Red and black stars separately indicated the phosphorylated SlPP2C72 and SlSLAH2. Experiments in (**a**–**i**) were independently repeated three times with similar results.

As shown above, SlPP2C72 indirectly repressed SlSLAH2 phosphorylation. However, SlPP2C72 maintained high expression level under normal conditions (Fig. S14a). To explore how Al^3+^ rapidly inhibited SlPP2C72 and promoted SlSLAH2 phosphorylation, we monitored SlPP2C72 phosphatase activity and phosphorylation level, since phosphatase activity and phosphorylation level were crucial for its dephosphorylation ability. Following Al^3+^ treatment, SlPP2C72 phosphatase activity was significantly inhibited (Fig. 8a), which was accompanied by an increase in its phosphorylation level (Fig. 8b). This resembled the Ca^2+^-mediated inhibition of PP2C.D6 by SCaBP8 (SOS3-LIKE Calcium Binding Protein 8) under salt stress in Arabidopsis^53^. Given SlCDPK21’s role as a calcium decoder, we hypothesized it might modulate SlPP2C72 activity through direct interaction and phosphorylation. Indeed, GFP-tagged SlCDPK21 significantly suppressed SlPP2C72 activity (Fig. 8c). Consistent with this, SlPP2C72 phosphatase activity was substantially elevated when co-incubated with *Slcdpk21* mutant (Fig. 8d). Further, Co-IP and LCI assays demonstrated SlCDPK21 interacted with SlPP2C72 (Figs. 8e and S19a). BiFC assay localized this interaction to the plasma membrane (Fig. S19b). Subcellular localization analysis revealed that SlCDPK21 was localized to the plasma membrane, whereas SlPP2C72 was localized to the nucleus, cytoplasm, and plasma membrane (Fig. S19c).

Since SlCDPK21 directly interacted with SlPP2C72 and inhibited its phosphatase activity, we speculated SlCDPK21 phosphorylated SlPP2C72 to inhibit its activity. When we co-incubated SlPP2C72 with protein extracts from WT or *Slcdpk21* mutants, under Al stress, we observed that the phosphorylation of SlPP2C72 exhibited a lower level compared to the WT to indicate SlCDPK21 phosphorylate SlPP2C72 (Figs. 8f and S20a). In vitro kinase assays demonstrated that SlCDPK21 phosphorylated SlPP2C72 (but not SlPP2C.D5) (Fig. 8g), and this phosphorylation was enhanced by Ca^2+^ (Fig. 8h). These results indicated that Al stress could trigger the phosphorylation of SlPP2C72 by SlCDPK21 to inhibit its phosphatase activity.

Given that SlCDPK21 could phosphorylate both SlSLAH2 and SlPP2C72, we tested whether it could do so simultaneously to safeguard SlSLAH2 phosphorylation. Co-incubation experiments revealed that SlCDPK21 could indeed phosphorylate both targets at once, but the presence of SlPP2C72 reduced SlSLAH2 phosphorylation (Figs. 8i and S20b).

**Fig. 9:**
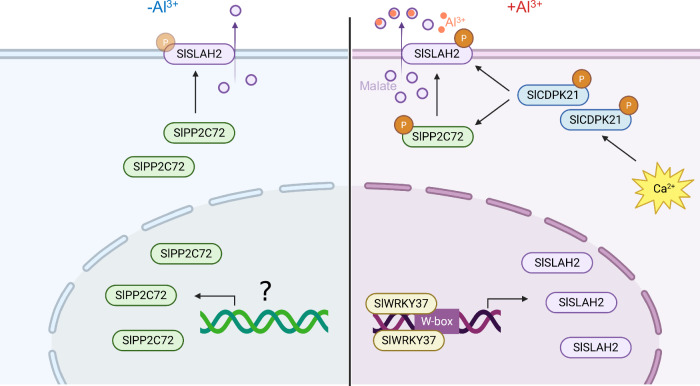
Working model of SlSLAH2-mediated malate exudation under aluminum stress in tomato. Al^3+^ triggered the SlWRKY37 to activate the expression of *SlSLAH2*, and Al^3+^ also activated SlCDPK21 to cause the phosphorylation of SlSLAH2 to promote malate exudation to increase tomato aluminum tolerance; Meanwhile, SlPP2C72 was suppressed by aluminum stress at the transcription level and the phosphorylation by SlCDPK21 reduced its phosphatase activity to disrupt the dephosphorylation of SlSLAH2, therefore, formnig a double-check to sustain SlSLAH2 phosphorylation. Created in BioRender. D, D. (2026) https://BioRender.com/ds8k1s3.

Collectively, these findings established a model: Al stress triggered phosphorylation of SlCDPK21, which phosphorylated and activated SlSLAH2 while simultaneously phosphorylating and inactivating SlPP2C72, thus suppressing its phosphatase activity. This dual action prevented SlSLAH2 dephosphorylation, maintaining channel activation and conferring Al tolerance. At the transcriptional level, activated by Al stress, SlWRKY37 bound the *SlSLAH2* promoter and stimulated its expression. This led to the formation of more malate transporters, which entered the subsequent phosphorylated state to sustain high malate exudation under Al stress. Once Al stress was alleviated, the transcription level of SlPP2C72 was increased and its phosphatase activity was restored, leading to SlSLAH2 dephosphorylation and preventing excessive malate exudation (Fig. 9).

## Discussion

### Divergent selection of malate transporters between tomato and other species for Al detoxification

This study revealed tomato-specific regulatory mechanisms of malate transporters under Al stress. Root exudation of organic acids (malate, citrate, or oxalate) increases Al tolerance, with ALMT-mediated malate exudation considered the typical Al chelation pathway. While transporters for citrate have been reported in tomato^54^, the research on malate transporters remains unclear. RNA-seq analysis of Al^3+^ treated WT tomato roots showed significant downregulation of nearly all *SlALMT*, which was confirmed by RT-qPCR (Figs. 1b and S1). Notably, malate was detected in the root exudation of WT tomato exposed to Al stress (Fig. 1a). This finding led us to investigate the specific malate transporters mediating Al^3+^-induced malate exudation in tomato.

Given the reported malate permeability of the SLAC/SLAHs family, we found *SlSLAH2*, which responded strongly to Al stress and was specifically highly expressed in roots (Figs. 1c, d and 2d). Subcellular localization, dicarboxylate transport functional complementation in *E. coli* and Electrophysiological analysis confirmed SlSLAH2 as a plasma membrane-localized malate transporter (Fig. 2a–c). Knockout of *SlSLAH2* resulted in reduced malate exudation and increased Al accumulation after Al^3+^ treatment, highlighting its role as a key malate transporter in tomato under Al stress (Fig. 2e, f). To exclude cultivar specificity, we also checked root *SlALMT* expression in several additional varieties and observed the same Al^3+^-dependent down-regulation in other varieties. Simultaneously, the expression of *SlSLAC*/*SLAHs* was upregulated under Al stress (Fig. S2).

Our study identified a previously unrecognized malate-exudation pathway in tomato, distinct from the classical ALMT-mediated mechanism. While previous research has focused almost exclusively on the ALMT family as the primary malate transporters under Al stress, we revealed a unique pathway in which SlSLAH2 emerged as a critical, yet previously unappreciated, mediator of malate transport.

However, previous work has shown that the tonoplast-localized SlALMT9 also played a significant role under Al stress: Overexpression lines of *SlALMT9* driven by the *CaMV35S* promoter substantially increased malate exudation during exposing to Al^3+^. The response to Al stress was conserved in AtALMT9 (the Arabidopsis homolog of SlALMT9), which was slowly activated by Al^3+^^55^. AtALMT9 was tonoplast-localized and functioned as a vacuolar malate channel that maintained vacuolar malate homeostasis^56^. This mechanism implied that vacuolar malate stores can be remobilized, presumably through transport, to meet root-exudation demand, while simultaneously alleviating Al toxicity by chelating Al within the cytoplasm of root cells.

Through phylogenetic tree analysis of the ALMT family in tomato and Arabidopsis, we found that SlALMT9 clustered within the same branch as AtALMT3/4/5/6/9, and exhibited the closest phylogenetic relationship to AtALMT4/5/6^57^. AtALMT5 has been identified as a malate and fumarate transporter system that mediated fumarate transport into the vacuoles of mesophyll cells. Knockout of *AtALMT5* disrupted the malate/fumarate ratio balance, resulting in malate accumulation^58^. AtALMT6 functioned as a calcium-activated vacuolar malate channel, subjected to multiple regulations by pH, membrane potential, and substrate concentration. It controlled malate flux and influenced stomatal movement^59^. AtALMT4 was also a vacuolar malate channel required for ABA-induced stomatal closure, establishing the mechanism for solute efflux in stomatal closing.

We proposed that the tonoplast localization of SlALMT9 indicated its function in intracellular malate transport. Based on the observed upregulation of *SlALMT9* promoter activity, as indicated by GUS expression, under high Al^3+^ concentration (200 μM), SlALMT9 may transport the malate from vacuole into cytoplasm, chelate internal Al, or serve as a backup source for plasma membrane malate exudation to meet the continuous demand for malate delivery to roots under Al stress. Furthermore, SlALMT9 likely cooperated with SlSLAC/SLAHs proteins, including SlSLAH2, to confer Al tolerance. This cooperative model mirrors the “dual-channel co-activation” paradigm observed in Arabidopsis guard cells, where S-type anion channels (AtSLAC1, AtSLAH3) and the R-type channel (AtALMT12/QUAC1) act in concert to trigger stomatal closure.

Thus, SlALMT9 and SlSLAH2 were proposed to function synergistically: During the early stages of Al stress, plant roots rapidly exudate malate by SlSLAH2 to chelate Al outside the rhizosphere. To prioritize resources for this immediate defense response, the plant may downregulate genes associated with other non-essential metabolic pathways, such as those involved in fruit acidity maintenance via SlALMT9. In contrast, during the later stages of Al stress, the plant activates an internal tolerance mechanism dominated by SlALMT9. The malate released by SlALMT9 could be utilized in the cytoplasm to chelate Al that have entered the cell, thereby mitigating intracellular toxicity. Alternatively, malate might be transported over long distances or through other means to the roots, providing substrate support for sustained, high-intensity malate exudation in the rhizosphere.

In addition to *SlSLAH2*, we also detected that *SlSLAH1* and *SlSLAH1-1* in the SlSLAH/SLAHs family respond to Al stress, with SlSLAH1 functioning downstream of SlSTOP1 to promote malate exudation^41^. Phylogenetic analysis revealed that SlSLAH1 and SlSLAH1-1 clustered closely with AtSLAH1, while SlSLAH2 was orthologous to AtSLAH3. In Arabidopsis, AtSLAH1 functions as a silent regulatory subunit that gates and activates AtSLAH3, a nitrate- and phosphorylation-dependent anion channel. When co‑expressed, they form a heteromeric complex, allowing sustained AtSLAH3-mediated anion currents even without nitrate or kinase activation. Thus, AtSLAH1 acts as both a functional activator and an environmental signal‑responsive switch for AtSLAH3, forming a key regulatory module that dynamically adjusts anion transport under stress conditions^60^.

Thus, we propose that SlSLAH1 or SlSLAH1-1 and SlSLAH2 may form a complex, in which SlSLAH1 and SlSLAH1-1 serve as regulatory subunits to enhance SlSLAH2-mediated malate exudation. Therefore, at least two Al stress-activated malate exudation pathways exist in tomato: the first is the SlSTOP1-mediated SlSLAH1 pathway, and the second is the SlWRKY37-mediated SlSLAH2 pathway. Ultimately, the Al stress signaling converges on SlSLAH2, establishing SlSLAH2 as the primary Al^3+^-induced malate transporter in tomato. This synergistic mechanism collaboratively enhances Al tolerance in tomato and amplifies the function of SlSLAH2 as an Al^3+^-induced malate exudation channel. Therefore, although SlSLAH2 did not function directly downstream of SlSTOP1, SlSLAH1 acted as an activator of SlSLAH2, integrating the Al stress-induced malate exudation pathway ultimately into SlSLAH2. In coordination with other Al stress-responsive genes regulated by SlSTOP1, collectively alleviates Al toxicity in tomato plants.

### SlCDPK21 linked the Al^3+^ signaling and the activation of anion channels

While Al stress is known to trigger cytosolic Ca^2+^ burst, the molecular mechanisms translating calcium signaling into effectors remain poorly understood. Previous studies proposed indirect regulatory models in wheat and Arabidopsis involving calmodulin (CaM), calcineurin B-like proteins (CBL1), and CML24-CAMTA2 complexes that regulated malate transporter expression^48,61,62^. Plant calcium decoding includes three principal sensor families: CaMs/ CMLs, CBLs, and CDPKs/CPKs^63^. Distinctively, CDPKs harbor both a calmodulin-like domain with EF-hand Ca^2+^ binding site and protein kinase domain, enabling direct calcium signaling to kinase signaling transduction^64,65^. Although CDPKs are reported to participate in many abiotic stress responses^66^, their specific roles in Al sensing and transducing have remained poorly understood.

Our study revealed significant phosphorylation of SlSLAH2 under Al stress (Figs. 5a and S9). We identified SlCDPK21 as a plasma membrane-localized kinase (Fig. S19c), which responded to Al stress (Fig. 5b) and physically interacted with SlSLAH2 (Fig. 5d–f), leading to the phosphorylation of SlSLAH2 (Fig. 6a, f). Three hypotheses have been proposed for Al^3+^ activation of anion channels: (1) direct Al^3+^ binding to transporters; (2) Al^3+^ interaction with membrane receptors or second messengers; (3) intracellular Al^3+^ activation^67^. Our findings supported the second hypothesis, demonstrating that Al^3+^ promoted the activation of anion channels through intracellular second messengers. Therefore, we proposed a signaling cascade model where Al stress triggered Ca^2+^ signaling, leading to SlCDPK21 phosphorylation and activation. The activated SlCDPK21 then interacted with and phosphorylated SlSLAH2, activating the channel to promote malate exudation for Al chelation. This study provided mechanistic evidence for CDPK-mediated calcium signal transduction in Al stress, establishing the Al^3+^-Ca^2+^-SlCDPK21-SlSLAH2-malate regulatory module for Al detoxification in plants.

A recent study has revealed that AtCPK21/23 functioned as a calcium sensor to transduce Al signals. They phosphorylated AtSTOP1, thereby inhibiting the binding of AtRAE1 to AtSTOP1 and enhancing stability of AtSTOP1. This mechanism activated *AtALMT1* expression, promoting malate exudation from roots to chelate Al, ultimately strengthening Al resistance in Arabidopsis^49^. Phylogenetic analysis revealed that SlCDPK21 shared close evolutionary proximity to AtCPK21/23 (Fig. S10b). Crucially, Al stress significantly enhanced SlCDPK21 activation (Fig. 5c). Knockout of *SlCDPK21* reduced the root elongation and malate exudation (Fig. 6d, e). These findings collectively indicated that SlCDPK21 acted as a key calcium sensor in tomato, transducing Al^3+^-triggered signals to downstream defense pathways to positively regulate Al tolerance in tomato.

Given the close phylogenetic relationship between SlCDPK21 and AtCPK21/CPK23, we hypothesized that SlCDPK21 might also regulate Al tolerance by phosphorylating SlSTOP1. Yet, BiFC assays failed to detect an interaction between SlCDPK21 and SlSTOP1 (Fig. S21a). Conversely, SlCDPK20, a closely related member, was found to interact with SlSTOP1 in the nucleus (Fig. S21a). Consistent with this, in vitro phosphorylation assays showed that SlCDPK20 could phosphorylate recombinant SlSTOP1 (Fig. S21b).

Thus, a multi-layered signaling network orchestrated by SlCDPK existed in tomato. This network integrated immediate physiological defense with long-term transcriptional adaptation by phosphorylating distinct key substrates, thereby efficiently enhancing Al tolerance. Specifically, the Al stress signal was channeled through different SlCDPK members into separate downstream pathways, forming a regulatory framework characterized by functional division and coordination. SlCDPK21 rapidly activated malate exudation by directly phosphorylating the plasma membrane-localized malate transporter SlSLAH2, providing a direct external detoxification barrier for the plant. Concurrently, SlCDPK20 phosphorylated the key transcription factor SlSTOP1, which promoted the transcription of downstream Al-responsive genes. This division of signaling labor mediated by distinct SlCDPK members ensured the simultaneous activation of both “rapid defense” and “long-term adaptation” strategies, enabling tomato to combat Al toxicity in a coordinated manner.

### A “Double-Check” mechanism for phosphorylation of SlSLAH2 under Al stress

Our study uncovered a sophisticated phosphorylation-regulatory circuit involving SlPP2C72, an E clade PP2C phosphatase whose expression and activity were suppressed under Al stress. SlPP2C72 indirectly regulated SlSLAH2 dephosphorylation through an unidentified intermediary, as there was no evidence of direct binding. Protein kinases can be dephosphorylated by phosphatases, thereby releasing their phosphorylation of downstream proteins^68^. For instance, OsPP2C09 negatively regulated rice drought resistance and promoted growth by dephosphorylating OsSAPKs (also known as osmotic stress/ABA-activated protein kinases, SnRK2s) to inhibit ABA signaling^69^. We hypothesized that indirect dephosphorylation involves the presence of one or more unknown intermediary factors. We speculated that such an intermediary factor could function through the following possibilities: (1) A scaffold protein might exist that can simultaneously bind to both SlPP2C72 and SlSLAH2. This would recruit the phosphatase into close proximity with its substrate, enabling specific dephosphorylation. (2) SlPP2C72 might dephosphorylate a specific protein kinase that acts upstream of SlSLAH2, indirectly leading to a reduction in the phosphorylation level received by SlSLAH2 to effectively create a dephosphorylation. A similar phenomenon has been reported. For instance, in maize, ZmSLAC1 is activated by phosphorylation through the ZmSIMK1-ZmMEK1 cascade. ZmPP84, an F-clade PP2C, negatively regulates drought tolerance by dephosphorylating ZmMEK1, thereby attenuating the phosphorylation level of ZmSLAC14^52^. Therefore, it was highly plausible that a protein kinase activating SlSLAH2 also exists in tomato, and the phosphorylation status of this kinase was regulated by SlPP2C72. Consequently, SlPP2C72 indirectly regulated the phosphorylation level of SlSLAH2.

We demonstrated that SlCDPK21 employed a dual-check strategy to maintain SlSLAH2 phosphorylation under Al stress: by directly phosphorylating SlSLAH2 through physical interaction, and by phosphorylating SlPP2C72, thereby inhibiting its phosphatase activity and preventing SlSLAH2 dephosphorylation. Previous studies have shown similar mechanisms. For example, in ABA signaling, CPK23 and OST1 (Open Stomata 1) phosphorylated and inhibited ABI1 (ABA Insensitive 1), while ABA further suppressed ABI1 activity to enable CPK21/CPK23-mediated activation of SLAC1^44^. Additionally, ABI4 phosphorylation was maintained by MPK3/6 (Mitogen-Activated Protein Kinase 3/6)-mediated inactivation of PP2C12 and promotion of J-AR (Junction Adventitious Roots) production^70^, BIK1 (Botrytis-Induced Kinase 1) phosphorylated PP2C38 to regulate downstream targets^71^.

Together, our results support a double-check model in which SlCDPK21 directly maintains SlSLAH2 phosphorylation and simultaneously inhibits SlPP2C72 activity under Al stress. When soil pH neutralizes and SlCDPK21 becomes inactive, SlPP2C72 recovers its phosphatase activity and expression, leading to SlSLAH2 dephosphorylation and preventing excessive malate loss.

### SlSLAH2 was regulated by multiple protein kinases under Al stress

Our study suggested that SlSLAH2 activation may be regulated by a multi-kinase regulatory module. While SlCDPK21 phosphorylated SlSLAH2, we also identified other SlCDPKs that physically interact with SlSLAH2 (Fig. S22), potentially contributing to its phosphorylation. This redundancy resembled the mechanism in AtSLAC1, where both ABA-dependent OST1^45,72^ and calcium-activated CPKs/CIPKs^44,46^ phosphorylated different residues to regulate stomatal closure. SLAH3, a homolog of SLAC1, could not be  phosphorylated by OST1, and it acquired CPK21^73^ or PBL27-mediated phosphorylation to control stomatal closure^27^. Therefore, complex environment enhances the activity of anion channels by orchestrating multiple kinases. In future studies, attention should be paid to other protein kinases that regulate SlSLAH2 phosphorylation, not limited to SlCDPK. This can contribute to refining the model of malate transporters’ response to Al stress signaling.

Through mass spectrometry, we identified T167 as a SlCDPK21- and SlPP2C72- targeted residue of SlSLAH2 (Figs. 6c and S18a). Mutation of T167 to A167 reduced phosphorylation but did not eliminate the signal, suggesting the presence of other regulatory sites by SlCDPK21, similar to the multilayered regulation of AtSLAC1, which had six OST1-mediated phosphorylation sites, and Ser59 being a shared target of OST1, CIPK23, and CPK6/23^46^. The phosphorylation-activated SlSLAH2 under Al stress may involve multiple kinases regulating the same or different sites to achieve synergistic phosphorylation. This multilayered phosphorylation control system enables plants to respond to complex environmental cues through a kinase network, providing a robust mechanism for stress adaptation without overcommitting to single signaling pathways. Amino acid sequence alignment revealed SlSLAH2 T167 was conserved with AtSLAH3 T187 (Fig. S11), a residue whose phospho-mimetic mutation (T187D) constitutively activates AtSLAH3 without kinase^73^. Structural modeling revealed that substituting T167 with A167 altered the N-terminal structure of SlSLAH2 (Fig. S23), supporting the role of T167 phosphorylation in channel activity regulation. Based on the protein sequence alignment of SLAH3 orthologs from diverse species, we found that the T167 residue in SLAH3 (T187 in Arabidopsis) was highly conserved (Fig. S24). Furthermore, motif 3 (^105^DFSMFRTKSTLSKQKS^120^), which maintained AtSLAC1 in a closed state when unphosphorylated, was conserved in SlSLAH2, with T167 located within this motif (Fig. S24). Therefore, T167 in SlSLAH2 was likely critical to its channel activity. Future studies will focus on the kinase involved in the phosphorylation of SlSLAH2, specific site phosphorylated by kinase and the function of phosphorylation sites. Especially, whether the T167 site of SlSLAH2 is the most important phosphorylation site that can affect its transport activity.

## Methods

### Plant materials and growth conditions

To obtain *SlSLAH2*, *SlCDPK21*, and *SlPP2C72* knockout lines, two potential guide sequences were selected based on CRISPOR website (http://crispor.tefor.net/). The sgRNAs (guide RNA) were cloned into the binary vector pBSE402 to generate knockout lines. The primers used were listed in Supplementary Table S1. The recombinant plasmid was transferred into *Agrobacterium tumefaciens* GV3101 and subsequently into MicroTom tomato cotyledons following the protocol^74^. This process resulted in the following constructs: *Slslah2*-*cr*-*2* (2 bp deletion), *Slcdpk21*-*cr*-*1* (1 bp deletion), *Slcdpk21*-*cr*-*4* (1 bp deletion), *Slpp2c72*-*cr*-*1* (1 bp deletion) and *Slpp2c72*-*cr*-*3* (4 bp deletion). These led to introduction of a premature stop codon. All of the knockout lines confirmed to be homozygous and Cas9-free.

For *SlSLAH2*, *SlCDPK21*, and *SlPP2C72* overexpressing lines and complementation lines, the coding sequence of *SlSLAH2*, *SlCDPK21*, and *SlPP2C72* excluding the stop codon, driven by native promoter were cloned into pCAMBIA-1305. The primers used were listed in Supplementary Table S1. This process resulted in the following constructs: *SlSLAH2p*::*SlSLAH2*-*6* (*SlSLAH2*-*K*-*6*), *SlSLAH2p*::*SlSLAH2*-*8* (*SlSLAH2*-*K*-*8*), *SlSLAH2p*::*SlSLAH2*-*8*/*Slwrky37* (*Slwrky37* + *2K*-*8*), *SlSLAH2p*::*SlSLAH2*-*8*/*Slslah2*-*cr*-*2* (*Slslah2*-*cr*-*2* + *2K*-*8*), *SlCDPK21p*::*SlCDPK21*-*1* (*SlCDPK21*-*OE*-*1*), *SlCDPK21p*::*SlCDPK21*-*5* (*SlCDPK21*-*OE*-*5*), *SlPP2C72p*::*SlPP2C72*-*1* (*SlPP2C72*-*OE*-*1*), *SlPP2C72p*::*SlPP2C72*-*2* (*SlPP2C72*-*OE*-*2*).

The *Slwrky37* mutant lines were described in previous study^43^, and the *CR*-*1* lines (34 bp deletion) were used in this study. The *Slstop1* mutant lines were described in previous study^37^, and the *Slstop1*-*6*-*11* lines (5 bp deletion) were used in this study. The *Slslah2*-*cr*-*95* (95 bp deletion) were described in previous study^41^.

Tomato cultivars, MicroTom, Cerise VFNT (TS-40), Ailsa Craig (TS-9), Hacienda Rosario (TS-135), Moyobamba (TS-129), Rowpac (TS-186) and TS-261 were used in this study.

The RNA-seq data for Al^3+^-treated MicroTom cultivar analyzed in this work were originally reported by Zhang et al.^37^. The RNA-seq data for Al^3+^-treated AC cultivar analyzed in this work were originally reported by Zhu et al.^54^. The tissue expression data was collected from Gene Expression in Tomato Tissues^50^.

All plant materials were cultivated in a plant growth chamber. The Al^3+^ treatment protocol was conducted following the protocol^37^. All Al^3+^ treatment phenotype observation experiments were independently performed three times, with similar results observed.

### RNA extraction and RT-qPCR

The root samples were ground using liquid nitrogen. Total RNA was extracted with Trizol reagent (abs60154, Absin, Shanghai, China). First-strand cDNA was synthesized from 1 μg total RNA using the Reverse transcription kit (AG11705, Accurate Biotechnology, Changsha, China) following the manufacturer’s protocol. RT-qPCR was conducted on QuantStudio6 instrument (Thermo Fisher Scientific, MA, USA) with ABScript II One Step SYBR Green RT-qPCR Kit (RK20404, ABclonal Technology, Wuhan, China). The expression analysis was performed with the 2^–△△Ct^ method. *SlUBI* was used as a reference gene. The primers were listed in Supplementary Table S1. Three biological replicates were performed for each test.

### Yeast one-hybrid assay (Y1H)

The full-length CDS of *SlWRKY37* was cloned into pGAD424 vector to produce SlWRKY37-pGAD424. The promoter fragment containing W-box was cloned into pLacZi vector. The primers used were listed in Supplementary Table S1. The SlWRKY37-pGAD424 and W-box-pLaczi were co-transferred into YM4271 yeast strain. As a negative control, the pGAD424 and W-box-pLaczi were also co-transferred into YM4271 yeast strain. The transformants were cultured on SD/-U/-L agar medium (PM2291, Coolaber Science & Technology, Beijing, China) at 29 °C for 3 days. The normal growing bacterial plaques were selected for X-gal filter assay^75^. The experiment was independently repeated three times with similar results.

### Dual-Luciferase reporter assay

For the activation of *SlSLAH2* by SlWRKY37, the full length CDS of *SlWRKY37* was cloned into pGreen II 62-SK vector to produce SlWRKY37-SK. The promoter fragment containing W-box was constructed into the pGreen II 0800-LUC vector. The primers used were listed in Supplementary Table S1. SlWRKY37-SK, pGreen II 62-SK empty vector, and W-box-LUC were transformed into *Agrobacterium* strain GV3101-P19-psoup, respectively. The *Agrobacterium* carrying mixtures of SlWRKY37-SK + W-box-LUC or pGreen II 62-SK + W-box-LUC were infiltrated into *N. benthamiana* leaves. The mixtures of pGreen II 62-SK + W-box-LUC were set as negative control. After 4 days, the LUC and REN signals of *N. benthamiana* leaves were detected by Dual Luciferase Assay System (K1136, APExBIO, Houston, USA).

For the combination of *SlSLAH2* and SlSTOP1, the promoter regions of *SlSLAH2*, spanning from -1000 to -1 bp upstream of the ATG and from -2000 to -1001 bp upstream of the ATG, were cloned into the pGreen II 0800-LUC vector. SlSTOP1-SK was constructed in previous research^76^. The combinations of the assay: SlSTOP1-SK + *SlSLAH2* pro-1-LUC, SlSTOP1-SK + *SlSLAH2* pro-2-LUC, pGreen II 62-SK empty vector + *SlSLAH2* pro-1-LUC (negative control), pGreen II 62-SK empty vector + *SlSLAH2* pro-2-LUC (negative control).

The experiments were independently repeated three times with similar results.

### Electrophoretic mobility shift assay (EMSA)

The full-length CDS sequence of *SlWRKY37* was cloned into the pMAL-p5x vector to produce SlWRKY37-MBP. The primers used were listed in Supplementary Table S1. The vector was expressed in  *E. coli* BL21 (DE3) for protein purification. Protein expression was induced with 0.5 mM isopropyl β-D-1-thiogalactopyranoside (IPTG) (CW7301S, CWBIO, Jiangsu, China) at 28 °C for 16 h. The recombinant proteins were purified with Dextrin Beads (SA077005, Smart-lifesciences, Changzhou, China). The 50-bp promoter fragments containing W-box or mutated fragments were labeled by biotin. The EMSA assay was performed with Chemiluminescent EMSA Kit (MH104, Coolaber Science & Technology, Beijing, China), following the manufacturer’s guidelines. The experiment was independently repeated three times with similar results.

### Subcellular localization assay

The full-length CDS sequence of *SlSLAH2*, *SlCDPK21*, *SlPP2C72*, excluding the stop codon, were cloned into pSuper1300-GFP vector, respectively. The primers used were listed in Supplementary Table S1. The constructs were transformed into *Agrobacterium* strain GV3101-P19-psoup respectively and then expressed in *N. benthamiana* leaves. PMH (Plasma Membrane-Localized H^+^-ATPase) was used to visualize plasma membrane. pSuper1300 was set as mock control. After infiltrating into *N. benthamiana* leaves, fluorescence was observed after 4-day incubation by a confocal laser scanning microscopy (Leica SP8, Leica Microsystems Inc., Illinois, USA). The experiments were independently repeated three times with similar results.

### Bimolecular fluorescence complementation assay (BiFC)

Full-length CDS sequence of *SlSLAH2* and *SlPP2C72* were cloned into the pCAMBIA1300-35S-N-YFPN respectively, and full-length CDS sequence of *SlCDPK21* and *SlPP2C72* were cloned into the pCAMBIA1300-35S-N-YFPC respectively. The primers used were listed in Supplementary Table S1. The combinations were respectively infiltrated into *N. benthamiana* leaves. After 4-day incubation, YFP fluorescence was detected by a confocal laser scanning microscopy (Leica SP8, Leica Microsystems Inc., Illinois, USA)^77^. The experiments were independently repeated three times with similar results.

### Co-immunoprecipitation assay (Co-IP)

Full-length CDS sequence of *SlSLAH2* and *SlPP2C72*, excluding the stop codon, were cloned into the pCAMBIA-1305, respectively. The primers used were listed in Supplementary Table S1. The constructs were transformed into *Agrobacterium* strain GV3101-P19-psoup, respectively and then expressed in *N. benthamiana* leaves. For Co-IP assays, total protein was extracted by extraction buffer (20 mM Tris-HCl, pH 7.4, 1 mM EDTA, 100 mM NaCl, 1% NP-40, 1 × protease inhibitor cocktail). After 12,000 × *g* centrifugation under 4 °C for 10 min, the supernatant was collected as the source of total protein. The supernatant was then incubated with α-GFP magnetic beads (PGM025, LABLEAD Inc., Beijing, China) for 2 h at 4 °C. Following the incubation, the beads were washed 3 times with IP buffer (50 mM Tris, 0.15 M NaCl, pH 7.4). Finally, 40 μl elution buffer was added to beads to recover the immunoprecipitated proteins. The eluted protein samples were then resolved by sodium dodecyl sulfate-polyacrylamide gel electrophoresis (SDS-PAGE) and subjected to immunoblot detection using α-FLAG (ab49763, Abcam, Cambridge, UK) and α-GFP antibodies (SLAB3000, Smart-lifesciences, Changzhou, China). The SuperKine™ West Pico PLUS Chemiluminescent Substrate was used for the detection (BMU101, Abbkine Biotechnology Co., Ltd). The experiments were independently repeated three times with similar results.

### Luciferase complementation imaging assay (LCI)

Full-length CDS sequence of *SlSLAH2* and *SlCDPK21*, excluding the stop codon, were cloned into pCAMBIA1300-nluc. Full-length CDS sequence of *SlPP2C72* and *SlCDPK21* were cloned into pCAMBIA1300-cluc. The primers used were listed in Supplementary Table S1. The combination constructs were co-expressed in *N. benthamiana* leaves. The luciferase images were captured after 4-day incubation. The experiments were independently repeated three times with similar results.

### Phosphorylation assay

The full-length CDS sequence of *SlSLAH2*-N, *SlSLAH2*^T167A^-N (mutant form with the T167 replaced with A167 residue) and *SlCDPK21-GFP* were cloned into the pMAL-p5x and pGEX-4T-1 vector respectively to produce SlSLAH2-N-MBP, SlSLAH2^T167A^-N-MBP and SlCDPK21-GST. Point mutations of SlSLAH2 were generated by site-directed mutagenesis following the protocols of the Gloria Nova HS 2×Master Mix (RK20717, ABclonal Technology, WuHan, China). The primers used are listed in Supplementary Table S1. The vector was expressed *E. coli* BL21 (DE3) for protein purification. The recombinant GST-proteins was purified with GST-tagged protein agarose beads (L-2002, Biolinkedin, Shanghai, China). The Al^3+^ treated or untreated tomato roots were extracted with protein extraction buffer as above described.

For Al^3+^-induced phosphorylation, fusion protein was co-incubated with root extracted protein for 2 h at 30 °C, and the mixtures were added with beads for 2 h at 4 °C. The beads were washed 3 times with lysis buffer (GST-tag: 140 mM NaCl, 2.7 mM KCl, 10 mM Na_2_HPO_4_, 1.8 mM KH_2_PO_4_, pH 7.3; MBP-tag: 200 mM NaCl, 20 mM Tris-HCl, 1 mM EDTA, pH 7.4) or IP buffer. SDS-PAGE protein loading buffer was added to beads to recover proteins. λPPase (New England Biolabs, MA, USA) was applied to abolish the phosphorylation.

For SlCDPK21-induced phosphorylation, fusion protein was co-incubated with SlCDPK21-GST protein in kinase reaction buffer (50 mM Tris-HCl [pH 7.5], 20 mM MgCl_2_, 2 mM DTT, and 1 mM ATP) for 1 h at 30 °C, and the mixtures were added with beads of substrate for 2 h at 4 °C. The beads were washed 3 times with lysis buffer or IP buffer. SDS-PAGE protein loading buffer was added to beads to recover proteins. λPPase was applied to abolish the phosphorylation.

For the semi-in vivo phosphorylation levels of SlSLAH2 or SlPP2C72, SlSLAH2-FLAG or SlPP2C72-FLAG was expressed in *N. benthamiana* leaves. Total protein was extracted four days after infiltration and purified using anti-FLAG agarose beads (SA042005, Smart-Lifesciences, Changzhou, China). The SlSLAH2-FLAG or SlPP2C72-FLAG protein was co-incubated with protein extracts prepared from WT, *Slcdpk21*, or *Slpp2c72* lines that had been treated with either 0 or 90 µM Al^3+^ for 3 h.

The α-Pan Phospho-ser/thr (α-pS/T) antibody (Ab117253, Abmart, Shanghai, China) was used to detect the phosphorylation of SlSLAH2-N-MBP, SlCDPK21-GST, SlPP2C72-FLAG, SlSLAH2^T167A^-N-MBP. SlSLAH2-N-MBP, SlSLAH2T167A-N-MBP, SlPP2C72-FLAG and SlCDPK21-GST proteins were detected with α-MBP antibody (HT701-01, TransGen Biotech, Beijing, China), α-FLAG, α-GST antibody (CW0084M, CWBIO, Jiangsu, China). Actin was used as a loading control detected by α-ACTIN antibody (AC038, ABclonal Technology, Wuhan, China). To detect the Ca^2+^-dependent kinase activity of SlCDPK21, CaCl_2_ was added to reaction system.

The phosphorylation experiments were independently repeated three times with similar results.

### Dephosphorylation assay

The dephosphorylation assay was conducted following the protocol with some modification^78^. For dephosphorylation of SlSLAH2-N-MBP by SlPP2C72-FLAG, SlSLAH2-N-MBP was pre-incubated with 90 μM Al^3+^ treated tomato roots protein extractions for 2 h at 30 °C. The mixtures were added with beads for 2 h at 4 °C. The beads were washed 3 times with lysis buffer. Elution buffer (30 mM maltose, 200 mM NaCl, 20 mM Tris-HCl, 1 mM EDTA, 1 mM DTT, pH 7.4) was added to beads to recover proteins. SlPP2C72-FLAG and SlPP2C.D4-FLAG were expressed in *N. benthamiana* leaves and then added to reaction buffer (50 mM HEPES, pH 7.4, 50 mM NaCl, 10 mM MgCl_2_, 10 mM MnCl_2_, 0.1% Triton X-100, and 1 mM DTT). The experiments were independently repeated three times with similar results.

### Phosphatase activity assay

The *N. benthamiana* leaves infiltrated by SlPP2C72-FLAG, SlCDPK21-GFP, GFP respectively, were used for extraction protein. The *N. benthamiana* leaves infiltrated by SlPP2C72-FLAG was co-incubated with protein extracts from 0 μM or 90 μM Al^3+^ treated WT or *Slcdpk21* mutants. The Al^3+^ treated or untreated tomato roots were extracted with protein extraction buffer as above described. For IP assay, total protein was incubated with α-FLAG affinity beads or α-GFP magnetic beads for 2 h at 4 °C. Following the incubation, the beads were washed 3 times with extraction buffer. Finally, 40 μl elution buffer was added to beads to recover the immunoprecipitated proteins.

Phosphatase assay was performed according to previous reported research^53^. The SlPP2C72-FLAG protein was incubated with substrate phosphor-peptides and assay buffer for 10 min. The reactions were stopped by adding molybdate dye (V2460, Promega, WI, USA). Then the absorbance was measured at 600 nm. The experiments were independently repeated three times with similar results.

### Complementation test of SlSLAH2 in *Escherichia coli*

This assay was conducted according to the method described previously^16^. The full-length CDS sequence of *SlSLAH2* was cloned into pKK223-3 vector. The primers used were listed in Supplementary Table S1. The SlSLAH2-pKK223-3 was transformed into dicarboxylate transport mutant strain CBT315. The pKK223-3 empty vector was transformed into CBT315 as a negative control and into wild type K12 as a positive control. Strains were grown on M9 medium supplemented with 10mM L-malic acid (L8090, Solarbio Science &Technology, Beijing, China) as the sole carbon source (pH 6.6) at 37 °C for 3 days. The experiment was independently repeated three times with similar results.

### Hematoxylin staining assay

Following a 6-h exposure of tomato roots to 90 μM Al^3+^, the roots were rinsed twice with distilled water and incubated in hematoxylin staining solution (G1140, Solarbio Science & Technology Co., Ltd, Beijing, China) for 30 min. After a thorough rinse to remove excess dye, the roots were visualized under a microscope. The experiment was independently repeated three times with similar results.

### Detection of root malate exudation

One-month-old tomato plants were treated with 0.5 mM CaCl_2_, 90 μM AlCl_3_ (pH 4.7) for 12 h with constant shaking on a horizontal shaker at room temperature. The malate exudation was determined using the NAD cycling-coupled enzymatic method^79^. The experiment was independently repeated three times with similar results.

### Electrophysiological analysis

For the voltage clamp experiment in *Xenopus* oocytes, the coding sequences of *SlSLAH2* was cloned into the pGEMHE vector. The primers used were listed in Supplementary Table S1. The cRNAs were prepared in vitro using a T7 RiboMAX^TM^ large-scale RNA production system (P1300, Promega, WI, USA). The oocytes were isolated from *Xenopus* laevis. Oocytes injected with RNA-free water were used as a negative control. Oocytes expressed 6 ng cRNA (SlSLAH2) were injected with extracellular solution including none anion or 50 mM anion (malate^2-^, NO_3_^-^, or Cl^-^). The membrane potential was stepped from a 1.45 s holding potential of 0 mV to a potential 60 mV to-150 mV (in 15 mV decrements,7.5 s duration) with a 4.05 s at 0 mV.

### Statistical analyses

Data were analyzed by ANOVA or student’s *t*-test by GraphPad Prism 10 software.

### Accession numbers

Sequence data from this article can be found in the Solanaceae Genomics Network (https://solgenomics.net/).

SlALMT1 (Solyc01g007080), SlALMT2 (Solyc01g007090), SlALMT3 (Solyc01g096140), SlALMT4 (Solyc03g096820), SlALMT5 (Solyc03g119640), SlALMT6 (Solyc05g009580), SlALMT7 (Solyc05g009590), SlALMT8 (Solyc06g061100), SlALMT9 (Solyc06g072920), SlALMT10 (Solyc06g074100), SlALMT11 (Solyc08g006990), SlALMT12 (Solyc08g082950), SlALMT13 (Solyc09g065070), SlALMT14 (Solyc10g081890), SlALMT15 (Solyc11g068970), SlALMT16 (Solyc11g071350), SlSLAC1 (Solyc08g079770), SlSLAH1 (Solyc04g080990), SlSLAH1-2 (Solyc07g051950), SlSLAH2 (Solyc06g036440), SlSLAH3 (Solyc03g007770), SlSLAH3-2 (Solyc09g014610), SlSLAH4 (Solyc03g031590), SlCDPK2 (Solyc04g009800), SlCDPK4 (Solyc01g006840), SlCDPK5 (Solyc01g006730), SlCDPK6 (Solyc10g076900), SlCDPK7 (Solyc05g056570), SlCDPK19 (Solyc07g064610), SlCDPK20 (Solyc02g032820), SlCDPK21 (Solyc03g031670), SlPP2C72 (Solyc08g065670), SlUBI (Solyc07g064130), SlWRKY37 (Solyc01g079360), SlSTOP1 (Solyc11g017140).

### Reporting summary

Further information on research design is available in the Nature Portfolio Reporting Summary linked to this article.

## Supplementary information

Supplementary Information

Reporting Summary

Transparent Peer Review file

## Source data

Source Data

## Data Availability

The authors declare that the data supporting the findings of this study are available within the paper and its supplementary information files. Source data are provided with this paper.
