# Supplementary material for: SlSLAH2 mediates malate exudation and contributes to aluminum tolerance
Source: Nat Commun. 2026 Apr 10;17:5040. doi: 10.1038/s41467-026-71651-1 (PMC13243466; doi:10.1038/s41467-026-71651-1)
Supplement: Supplementary file 1 — Supplementary Information [file 41467_2026_71651_MOESM1_ESM.pdf]

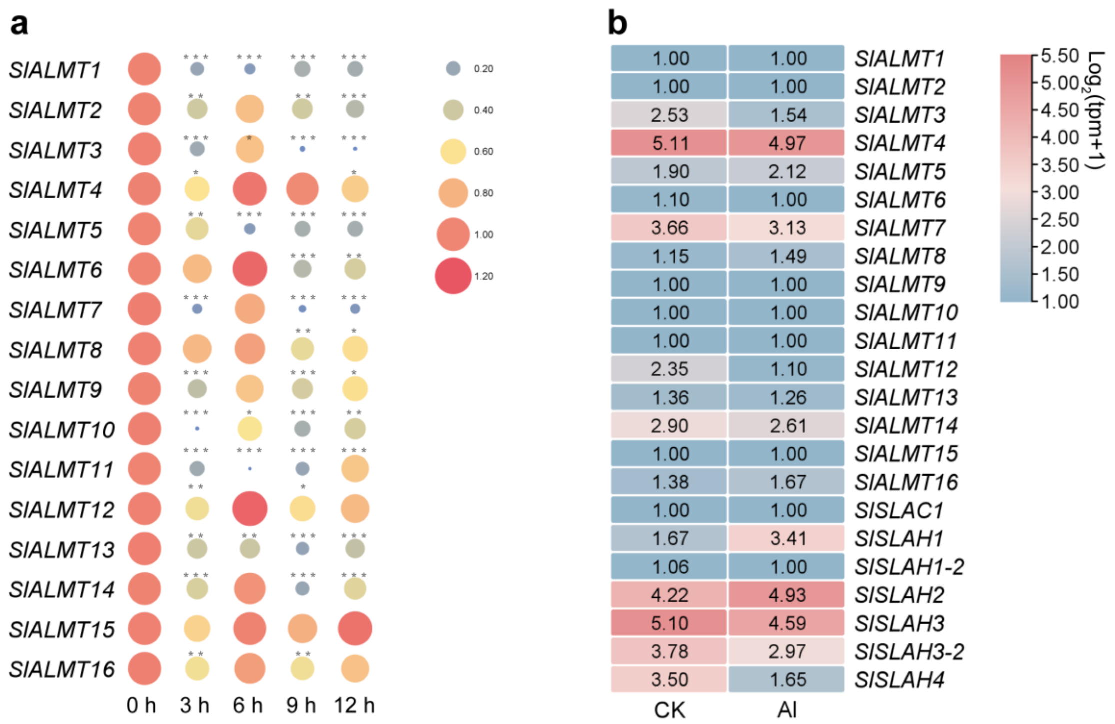

Figure S1. The temporal expression profile of the *SlALMT* genes in tomato under Al stress. (a) The expression levels of *SlALMT* were detected by RT-qPCR in 14-day-old MicroTom WT under treatment with 60 μM $AlCl_3$ (pH 4.7) in 12 hours. *SlUBI* was used as reference gene. Bubble size and color lightness corresponded to relative expression values, with larger/lighter bubbles indicating higher expression. Data were presented as means (n=3). Statistical significance was analyzed by one-way ANOVA (Dunnett's multiple comparisons test, **p* ≤ 0.05; ***p* ≤ 0.01, ****p* ≤ 0.001). **(b)** Heatmap analysis of *SlALMT* gene expression in AC WT tomato treated with or without $AlCl_3$. Expression values were shown as $\log_2{}^{(tpm+1)}$ (n=3).

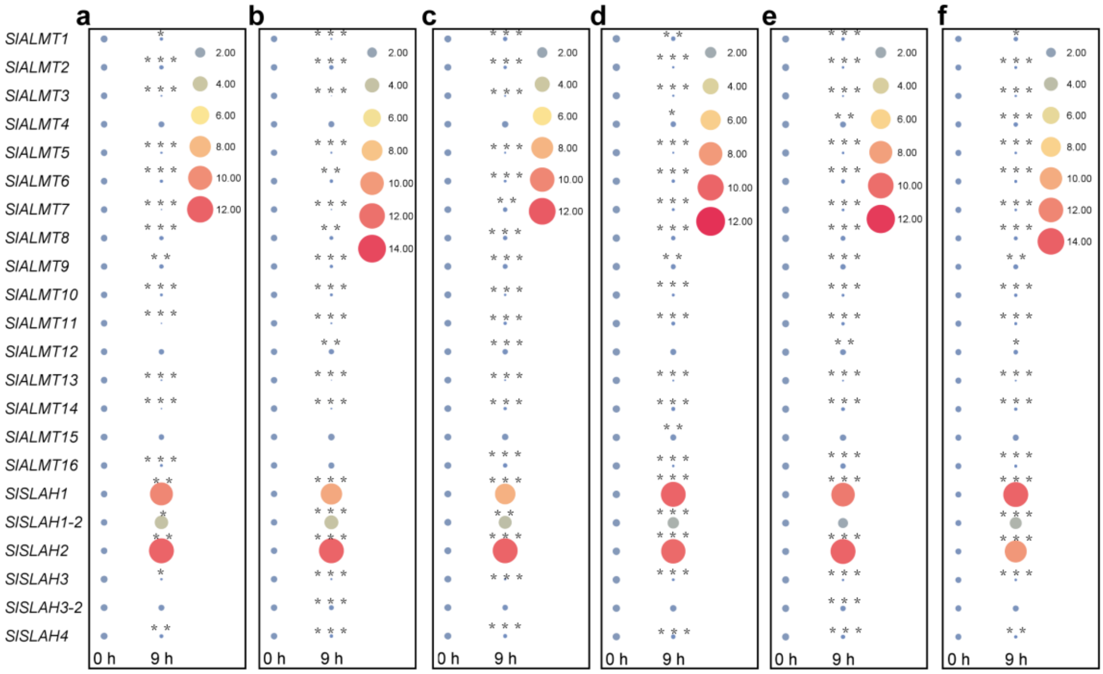

Figure S2. The temporal expression profile of the *SlALMT* and *SlSLAC/SLAHs* genes in tomato under Al stress. The expression levels of *SlALMT* and *SlSLAC/SLAHs* were detected by RT-qPCR in different tomato cultivars under treatment with 60 μM $AlCl_3$ (pH 4.7) in 12 hours. **(a)** Cerise VFNT (TS-40), **(b)** AC (TS-9), **(c)** Hacienda Rosario (TS-135), **(d)** Moyobamba (TS-129) **(e)** Rowpac (TS-186) **(f)** TS-261. *SlUBI* was used as reference gene. Bubble size and color lightness corresponded to relative expression values, with larger/lighter bubbles indicating higher expression. Data were presented as means (n=3). Statistical significance was analyzed by one-way ANOVA (Dunnett's multiple comparisons test, *$p \leq 0.05$; **$p \leq 0.01$, ***$p \leq 0.001$).

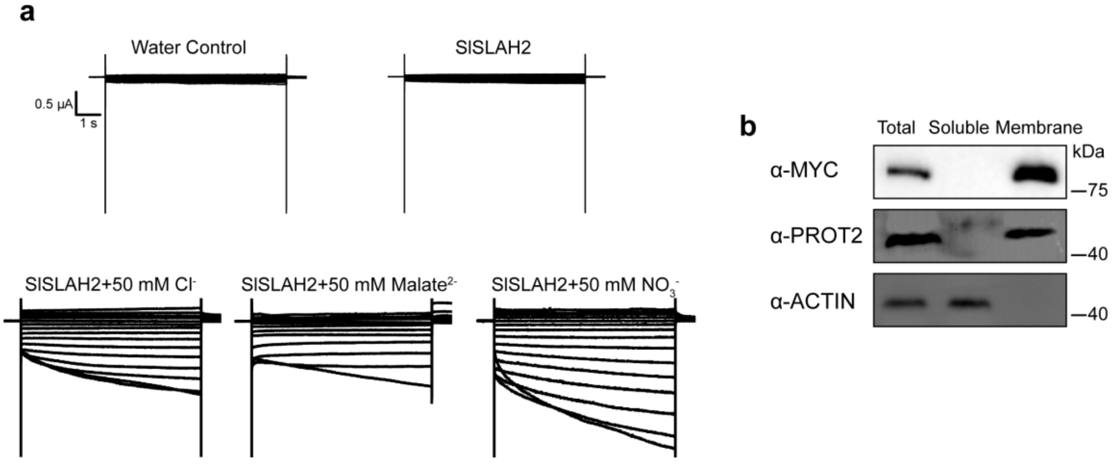

Figure S3. **SlSLAH2 was a plasma membrane localized anion transporter. (a)** Typical whole-cell current traces were recorded from the *Xenopus* oocytes injected with water perfused with extracellular solution including none anion or from the oocytes injected with SlSLAH2 perfused with extracellular solution including none anion, 50 mM NaCl, 50 mM $NaNO_3$ or 50 mM $C_4H_4O_5Na_2$ (Malate^{2-}). **(b)** Immunoblotting confirmed MYC tagged SlSLAH2 in the membrane protein components extracted from *N. benthamiana leaves.* PROT2, membrane protein marker; ACIN, soluble protein marker. Experiment was independently repeated three times with similar results.

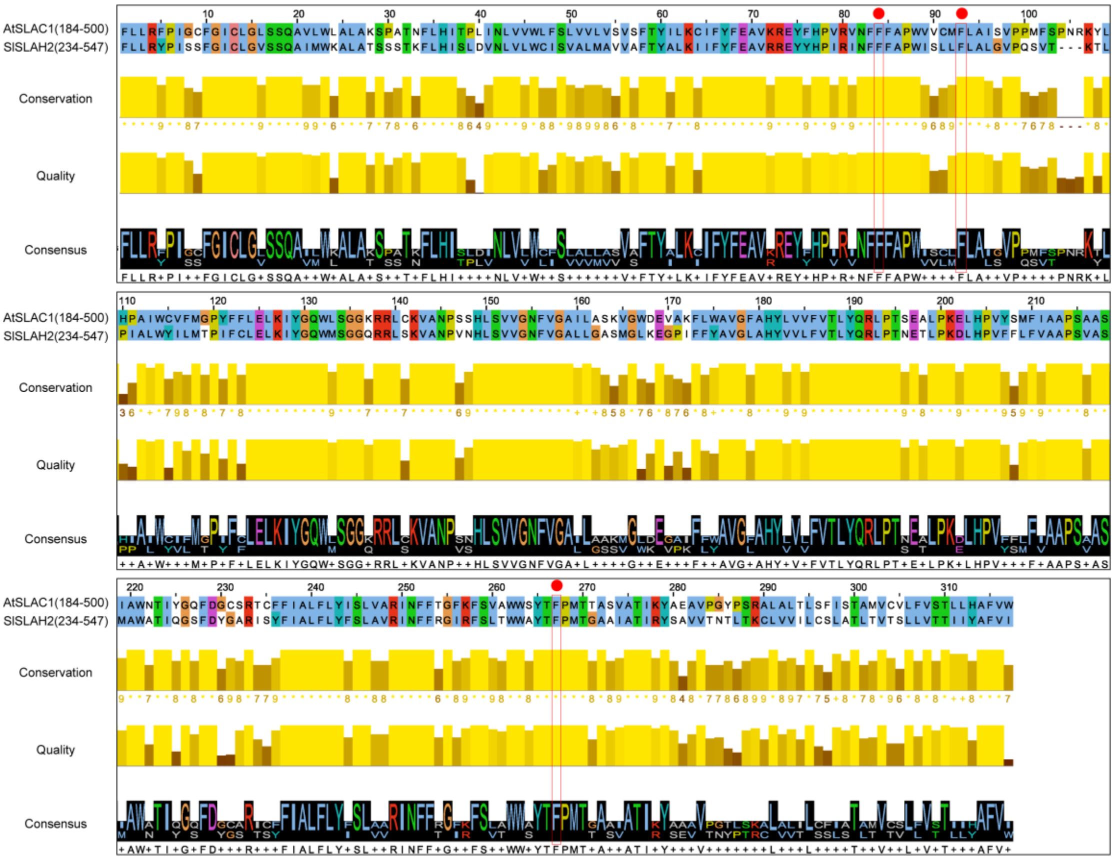

Figure S4. Multiple sequence alignment of SlSLAH2 and AtSLAC1. Multiple protein sequence alignment of the transmembrane domains of SlSLAH2 and AtSLAC1. The phenylalanines (F) in AtSLAC1 were highlighted with red circles and boxes.

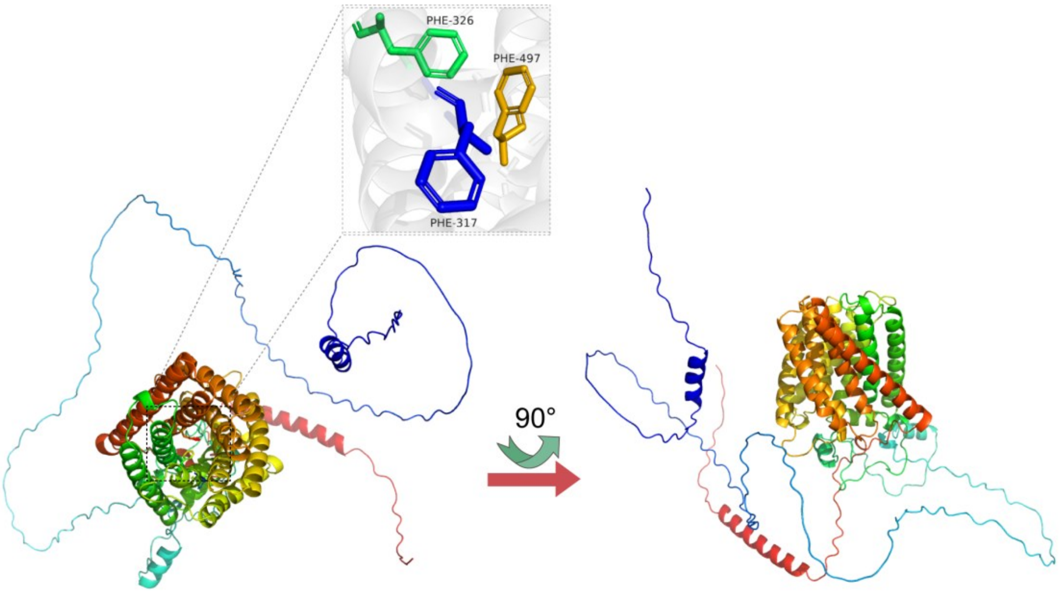

Figure S5. Prediction of the structure of SlSLAH2 protein. Ribbon diagram of SlSLAH2. Viewed from outside (left) and within (right) the membrane. The side chains of F317, F326 and F497 were shown as stick bonds within the channel pore. The structure was predicted by AlphaFold3 and draw by PyMOL.

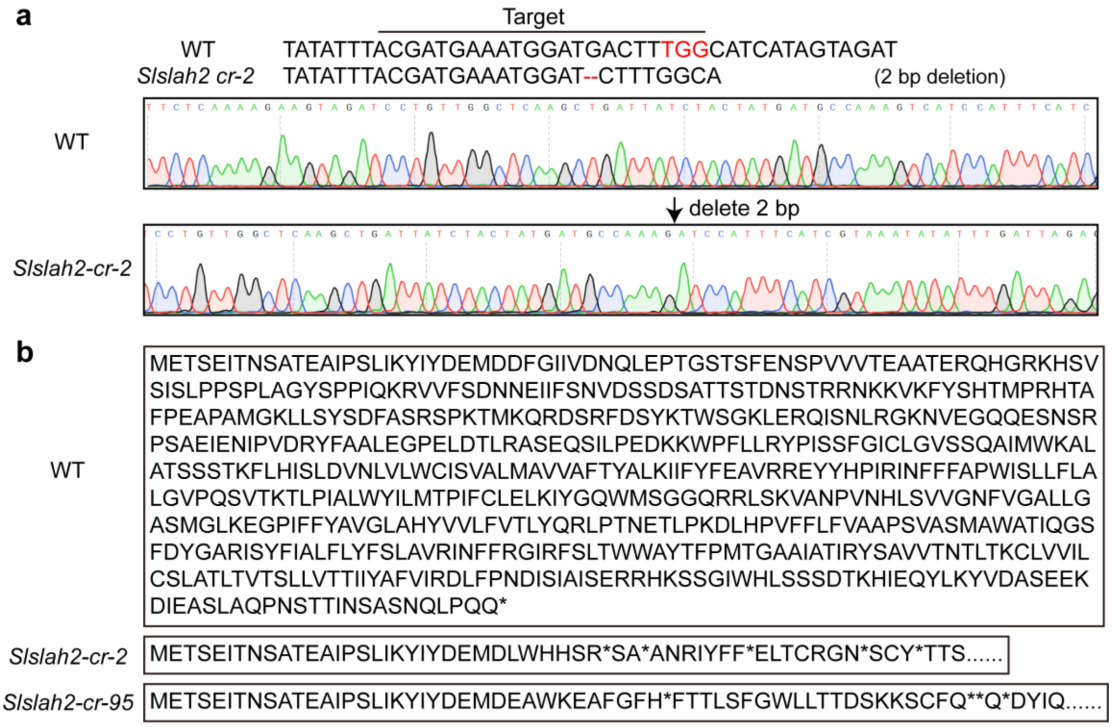

Figure S6. Genotyping of *SlSLAH2* CRISPR mutants. (a) Gene-editing strategy for the *SlSLAH2* knockout lines (above) and sequencing chromatogram of WT and *SLSLAH2* knockout lines (below). **(b)** The protein sequences of SlSLAH2 in WT, *Slslah2-2* and *Slslah2-95*.

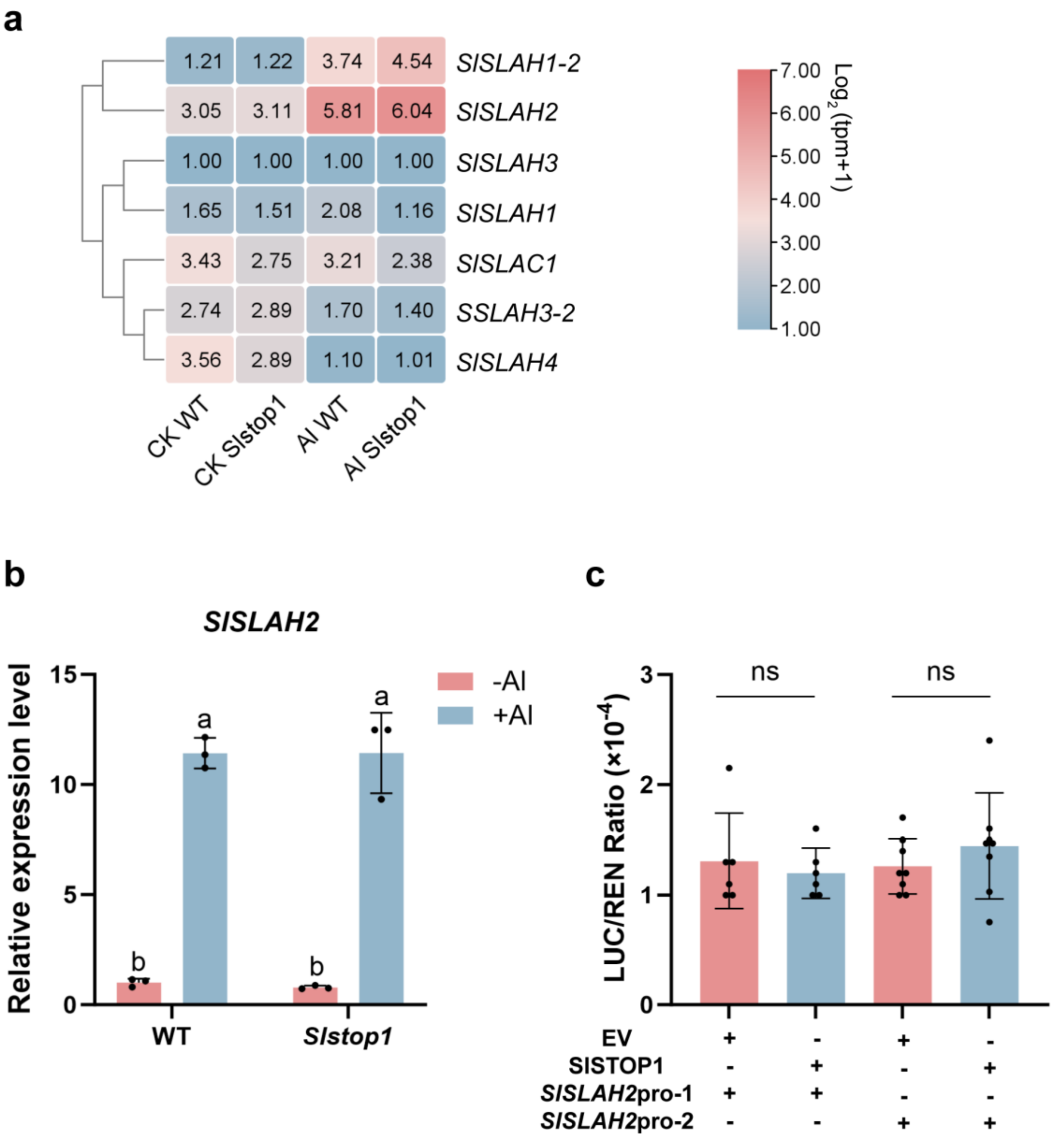

Figure S7. *SlSLAH2* was not a downstream target gene of SlSTOP1. (a) Heatmap analysis of *SlSLAC*/*SLAHs* genes in WT and *Slstop1* mutant treated with or without 60 μM $AlCl_3$ for 9 h (pH 4.7). Expression values were shown as $\log_2{}^{(tpm+1)}$ (n=3). **(b)** The expression level of *SlSLAH2* in WT and *Slstop1* mutant treated with or without 60 μM $AlCl_3$ for 9 h (pH 4.7) was detected by RT-qPCR. *SlUBI* was used as reference gene. Data were presented as means ± SD (n=3). Statistical significance was analyzed by two-way ANOVA, different lowercase letters indicated significantly different means (Tukey's multiple comparisons test, $p \leq 0.05$). **(c)** LUC/REN assay demonstrated the SlSTOP1 could not activate *SlSLAH2*. The promoter segment, which was cloned into pGreenII 0800-LUC vector, was set as reporter. EV (pGreen II

62-SK) co-expressing with reporter respectively was set as negative control. Data were presented as mean ± SD (n=6, 8). Statistical significance was analyzed by paired two-tailed *t*-test (ns, $p > 0.05$).

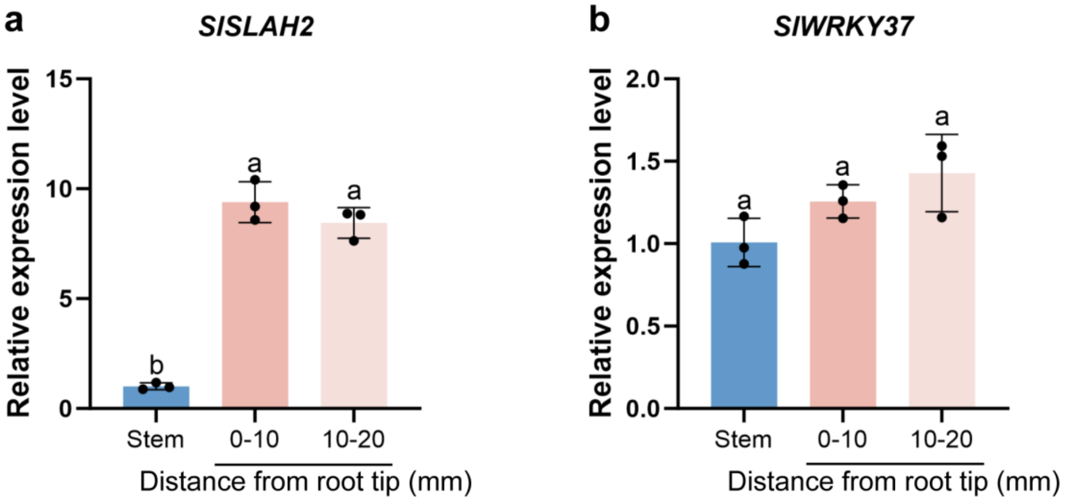

Figure S8. ***SlSLAH2*** **and** ***SlWRKY37*** **were expressed at root tip.** Relative expression levels of *SlSLAH2* **(a)** and *SlWRKY37* **(b)** in root tips (ranging from 0–10, and 10–20 mm) and stem. Data were presented as means ± SD (n=3). Statistical significance was analyzed by one-way ANOVA, different lowercase letters indicated significantly different means (Tukey's multiple comparisons test, $p \leq 0.05$).

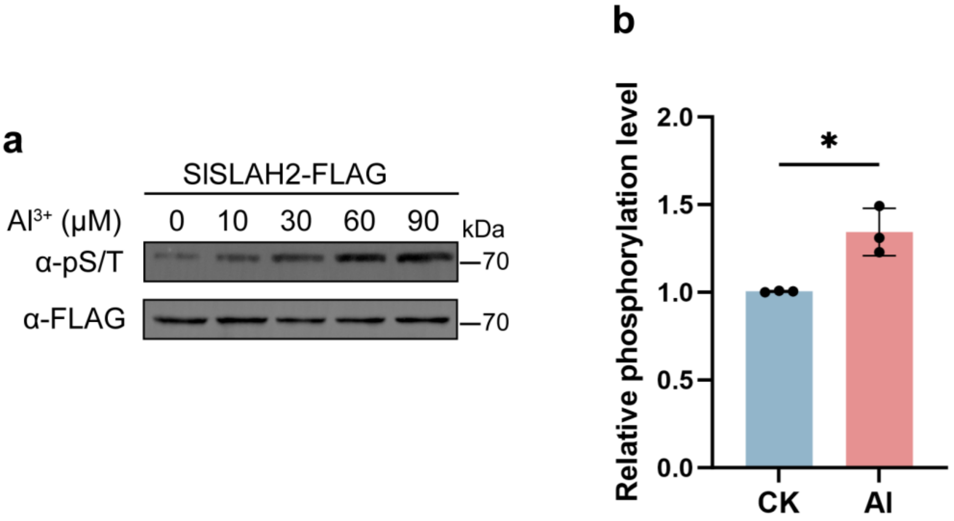

Figure S9. Al^{3+} increased the phosphorylation of SlSLAH2. (a) Phosphorylation of SlSLAH2 induced by a gradient of Al^{3+} concentrations. Total proteins were extracted from *SlSLAH2* overexpression lines treated with increasing concentrations of $AlCl_3$. The experiment was independently repeated three times with similar results. Experiment was independently repeated three times with similar results. **(b)** The statistical analysis of signal intensity in the western blot assays depicted in Figure 5a. Data were presented as means ± SD (n=3). Statistical significance was analyzed by paired two-tailed *t*-test (*$p \leq 0.05$).

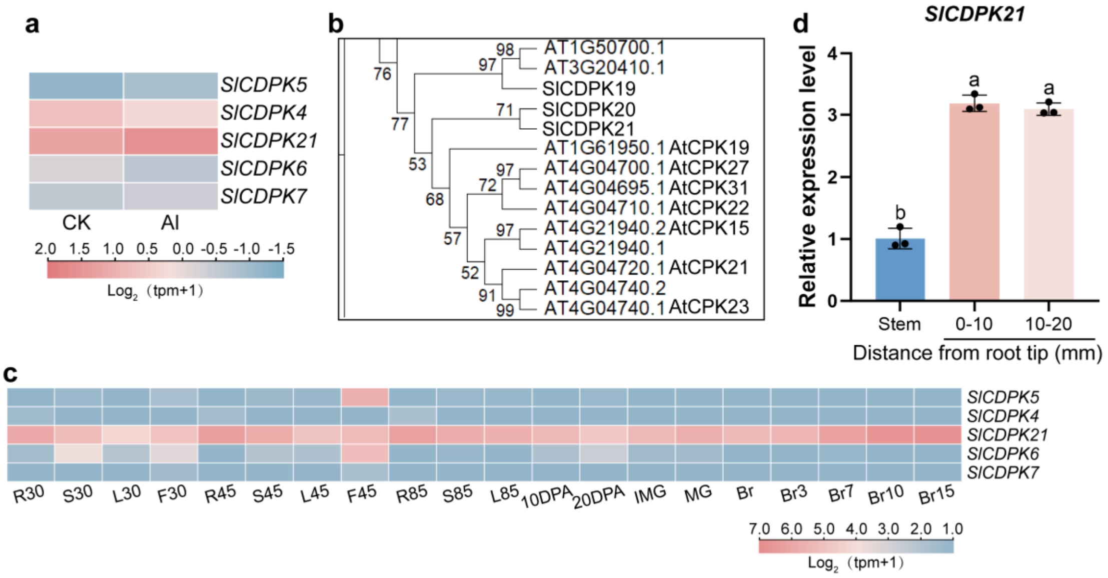

Figure S10. ***SlCDPK21*** **responded to Al stress and was expressed at root. (a)** Heatmap analysis of *SlCDPK* genes in WT treated with or without 60 µM $AlCl_3$ for 9 h (pH 4.7). **(b)** Part phylogenetic tree of SlCDPK in tomato and AtCDPK in Arabidopsis. **(c)** Tissue expression of *SlCDPKs*. The data was collected from Gene Expression in Tomato Tissues. Bud stage (30 DPG): (F 30)-Bud, (L 30)-Leaf, (S 30)-Stem, (R 30)-Root. Flowering stage (45 DPG): (F 45)-Flower, (L 45)-Leaf, (S 45)-Stem, (R 45)-Root. Breaker stage (85 DPG): (L 85)-Leaf, (S 85)-Stem, (R 85)-Root. (10 DPA)-55 DPG. (20 DPA)-65 DPG. (IMG)-75 DPG. (MG)-80 DPG. (Br)-85 DPG. (Br 3)-88 DPG. (Br 7)-92 DPG. (Br 10)-95 DPG. (Br 15)-100 DPG. **(d)** Relative expression levels of *SlCDPK21* in root tips (ranging from 0–10, and 10–20 mm) and stem. Data were presented as means ± SD (n=3). Statistical significance was analyzed by one-way ANOVA, different lowercase letters indicated significantly different means (Tukey's multiple comparisons test, $p \leq 0.05$).

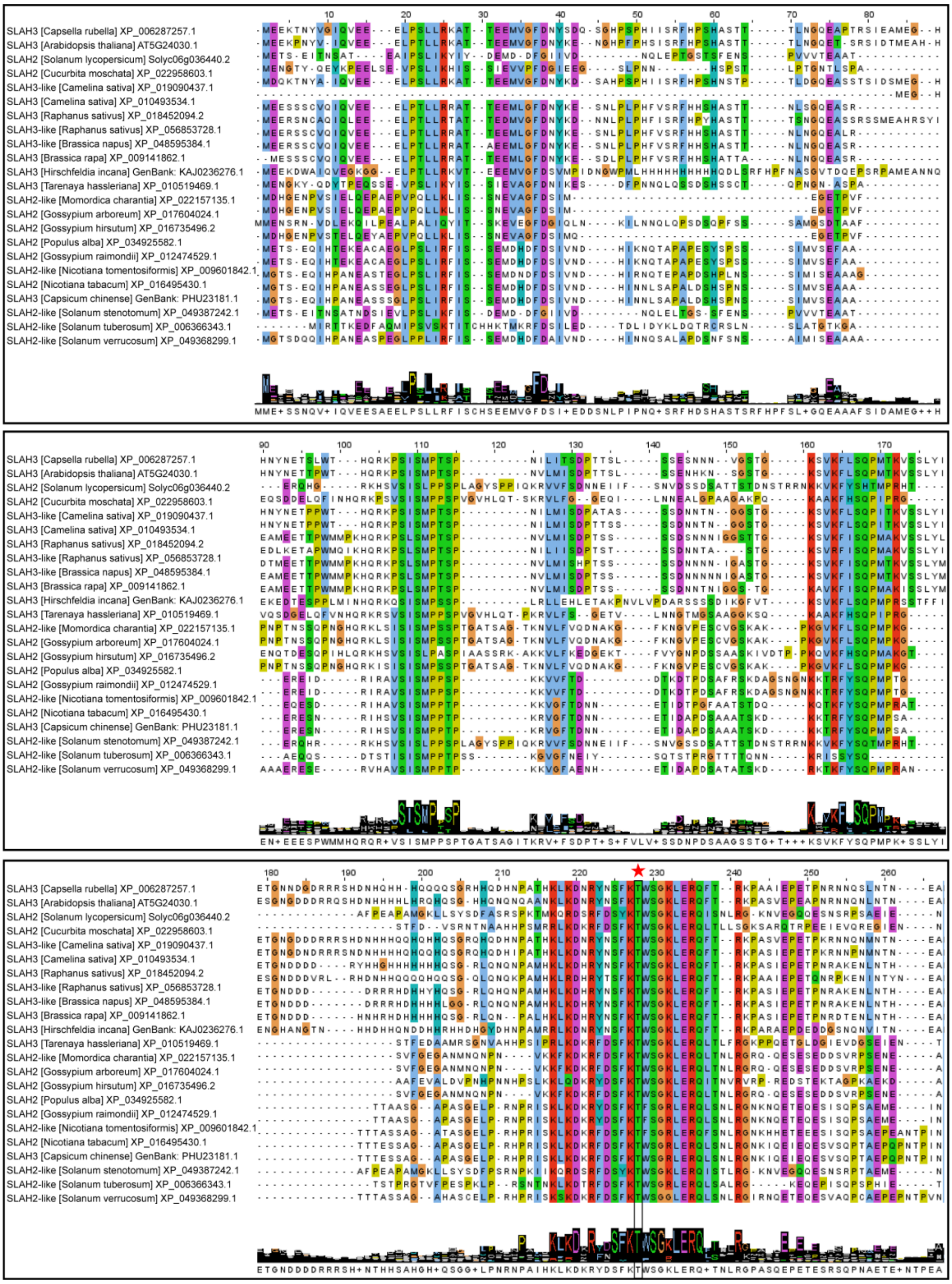

Figure S11. Multiple sequence alignment of SLAH2 from different species. The T167 site residue was marked with a red star. Black boxes indicated the conserved T167 in different species.

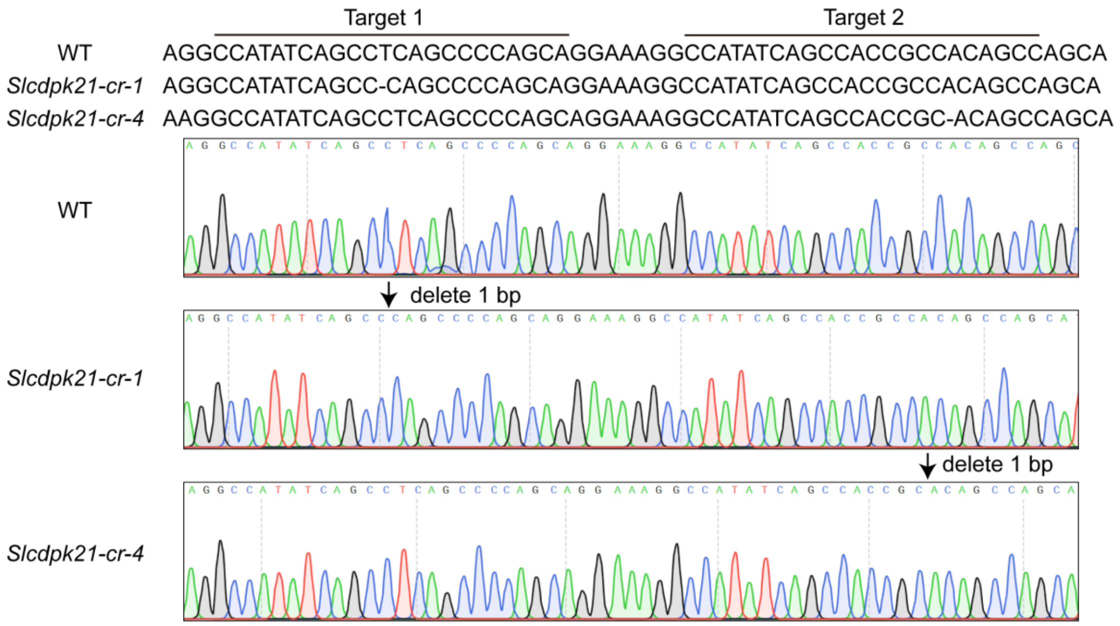

Figure S12. Genotyping of *SlCDPK21* CRISPR mutants. Gene-editing strategy for the *SlCDPK21* knockout lines (above) and sequencing chromatogram of WT and *Slcdpk21* mutant lines (below).

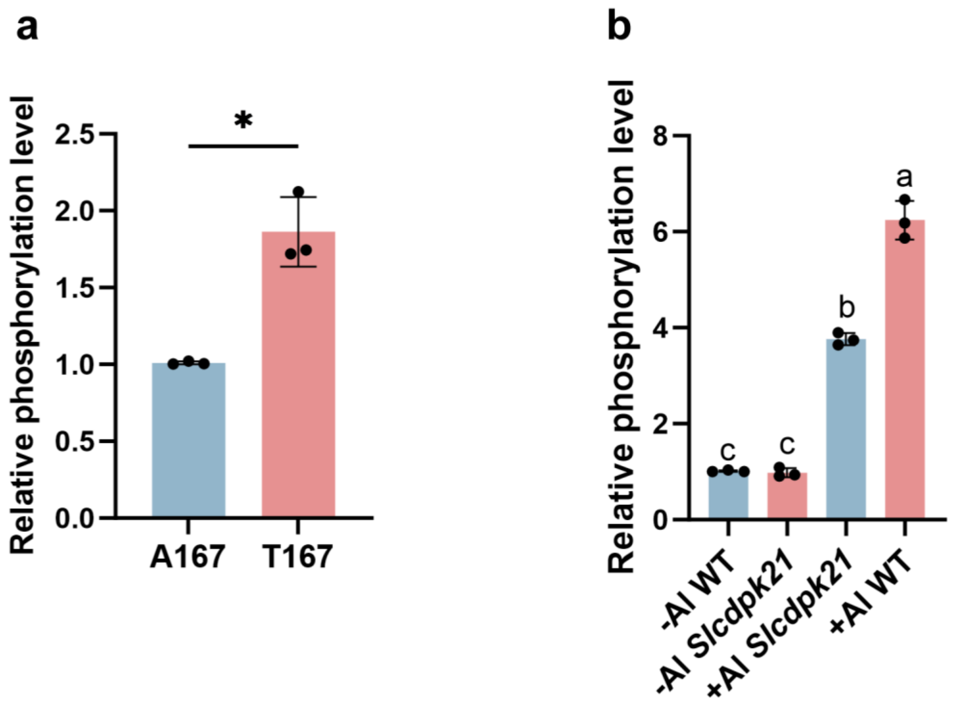

Figure S13. Statistical analysis of signal intensity. The statistical analysis of signal intensity in the western blot assays depicted in Figure 6c and Figure 6f. Data were presented as means ± SD (n=3). Statistical significance was analyzed by **(a)** paired two-tailed *t*-test (*$p \leq 0.05$) and **(b)** one-way ANOVA, different lowercase letters indicated significantly different means (Tukey's multiple comparisons test, $p \leq 0.05$).

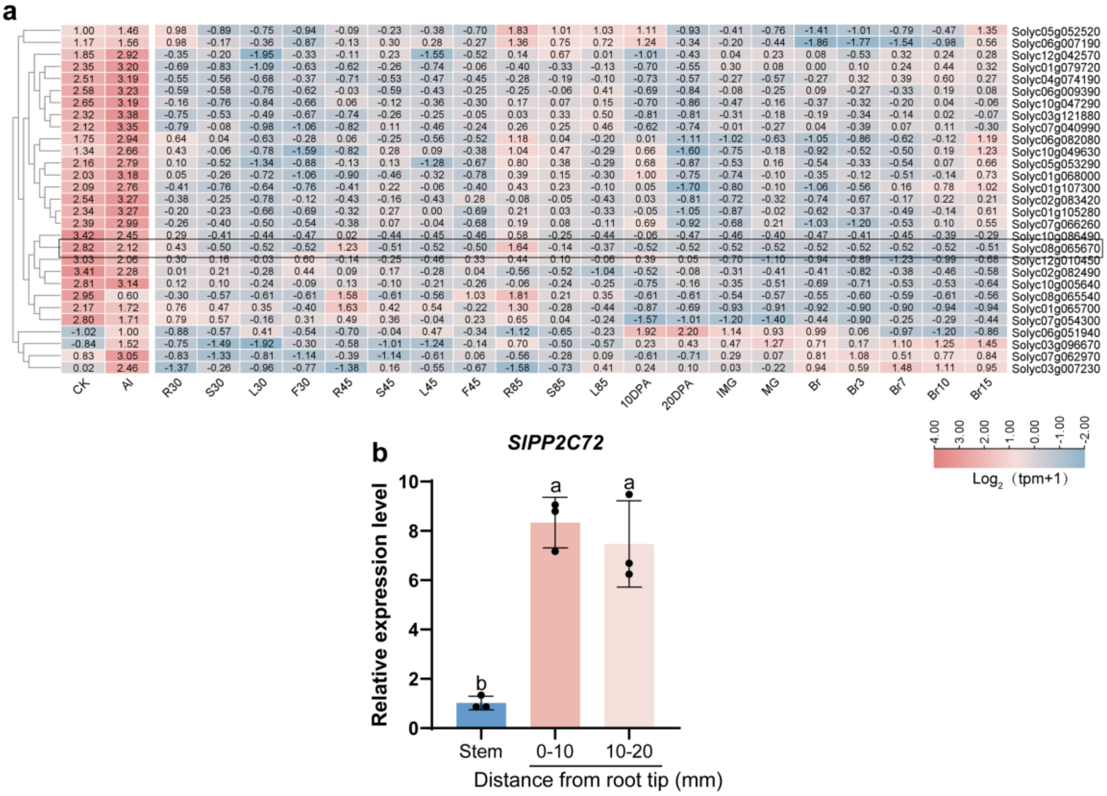

Figure S14. *SlPP2C72* was inhibited by Al stress and expressed at root tip. (a) Heatmap analysis of *SlPP2C* genes (left) in WT treated with or without 60 µM $AlCl_3$ for 9 h (pH 4.7), (right) in different tissue. The data was collected from Gene Expression in Tomato Tissues. Bud stage (30 DPG): (F 30)-Bud, (L 30)-Leaf, (S 30)-Stem, (R 30)-Root. Flowering stage (45 DPG): (F 45)-Flower, (L 45)-Leaf, (S 45)-Stem, (R 45)-Root. Breaker stage (85 DPG): (L 85)-Leaf, (S 85)-Stem, (R 85)-Root. (10 DPA)-55 DPG. (20 DPA)-65 DPG. (IMG)-75 DPG. (MG)-80 DPG. (Br)-85 DPG. (Br 3)-88 DPG. (Br 7)-92 DPG. (Br 10)-95 DPG. (Br 15)-100 DPG. **(b)** Relative expression levels of *SlPP2C72* in root tips (ranging from 0–10, and 10–20 mm) and stem. Data were presented as means ± SD (n=3). Statistical significance was analyzed by one-way ANOVA, different lowercase letters indicated significantly different means (Tukey's multiple comparisons test, $p \leq 0.05$).

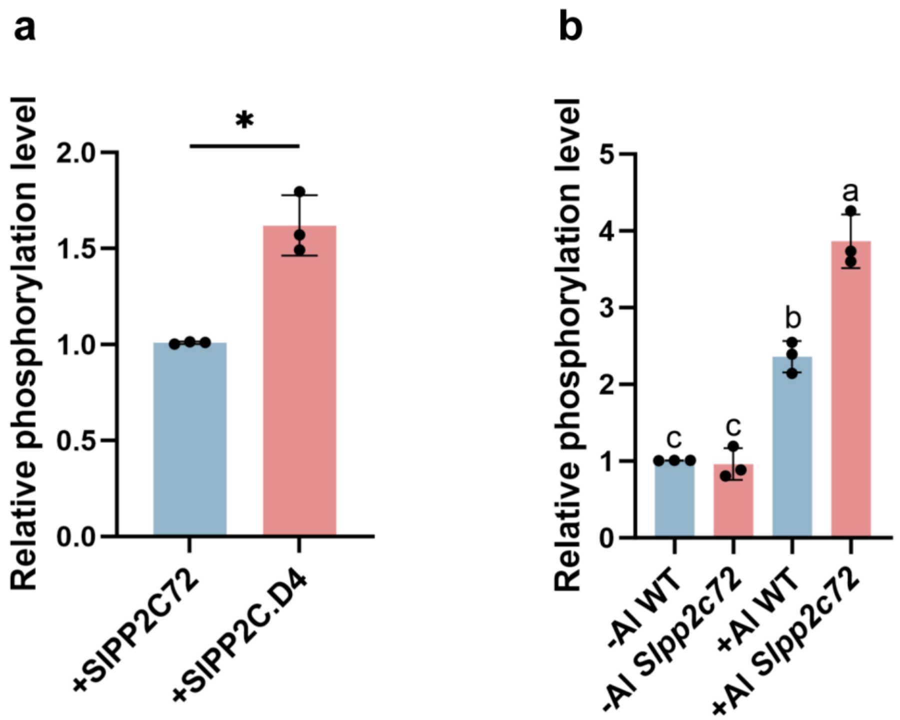

Figure S15. Statistical analysis of signal intensity. The statistical analysis of signal intensity in the western blot assays depicted in Figure 7b and Figure 7f. Data were presented as means ± SD (n=3). Statistical significance was analyzed by **(a)** paired two-tailed *t*-test ($*p \leq 0.05$) and **(b)** one-way ANOVA, different lowercase letters indicated significantly different means (Tukey's multiple comparisons test, $p \leq 0.05$).

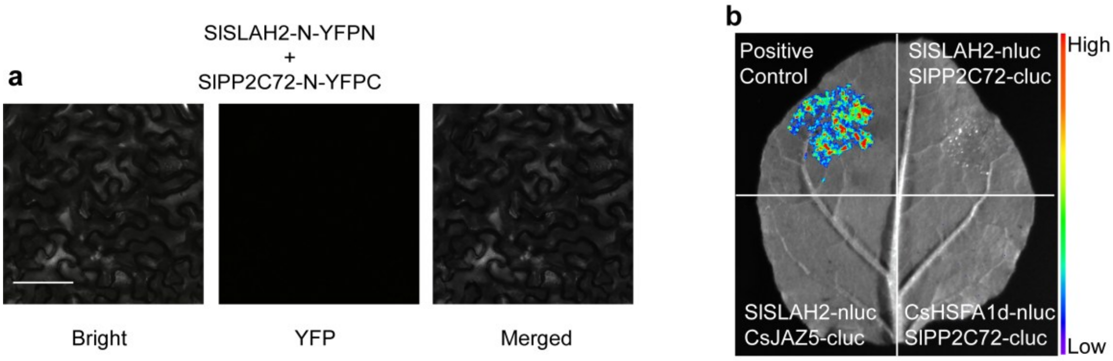

Figure S16. SlPP2C72 did not interact with SlSLAH2. (a) BiFC assay showed no interaction between SlPP2C72 and SlSLAH2. SlPP2C72-N-YFPC and SlSLAH2-N-YFPN proteins were transiently co-expressed in *N. benthamiana*. Scale bar, 50 μm. **(b)** LCI assay showed no interaction between SlPP2C72 and SlSLAH2. Constructs carrying SlPP2C72-cluc and SlSLAH2-nluc were co-expressed in *N. benthamiana* leaves for 3 d. CsJAZ5-cluc and CsHSFA1d-nluc were used as positive control. Experiments in (a) and (b) were independently repeated three times with similar results.

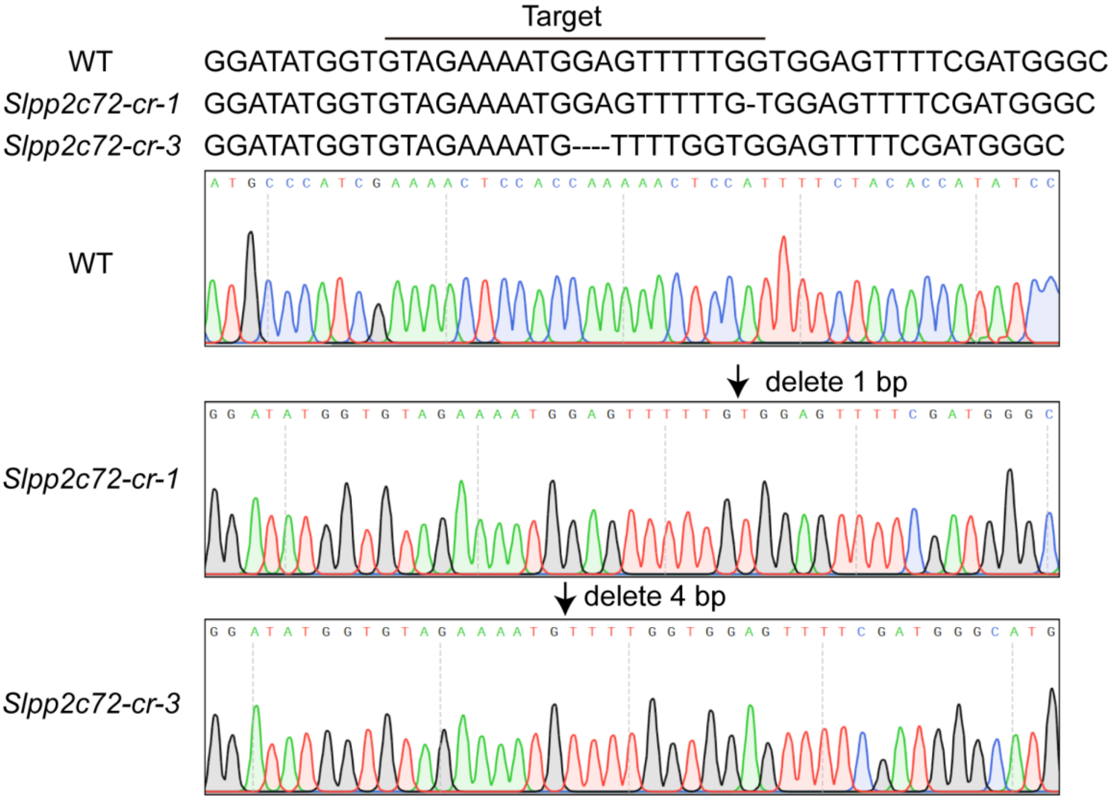

Figure S17. Genotyping of *SlPP2C72* CRISPR mutants. Gene-editing strategy for the *SlPP2C72* knockout lines (above) and sequencing chromatogram of WT and *Slpp2c72* mutant lines (below).

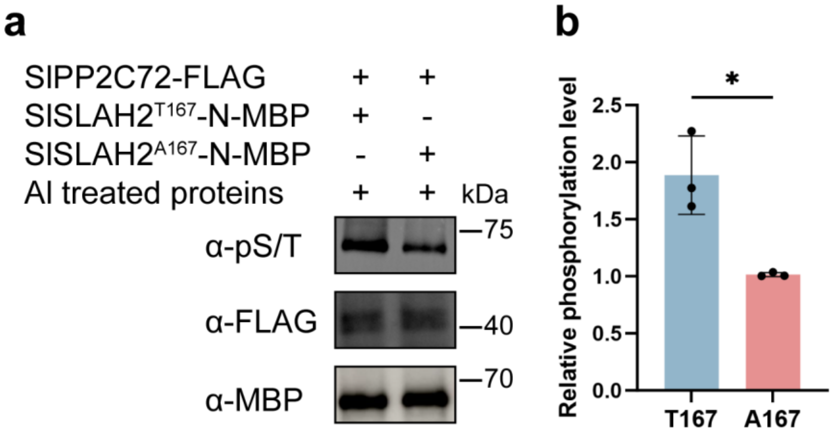

Figure S18. T167 of SlSLAH2 was a target of SlPP2C72. (a) Recombinant SlSLAH2-N-MBP and SlSLAH2^{A167}-N-MBP were co-incubated with 90 μM $AlCl_3$ treated tomato root extracted proteins. Then SlPP2C72-FLAG protein was added to this system. The experiment was independently repeated three times with similar results. Experiment was independently repeated three times with similar results. **(b)** The statistical analysis of signal intensity in (a). Data were presented as means ± SD (n=3). Statistical significance was analyzed by paired two-tailed *t*-test ($*p \leq 0.05$).

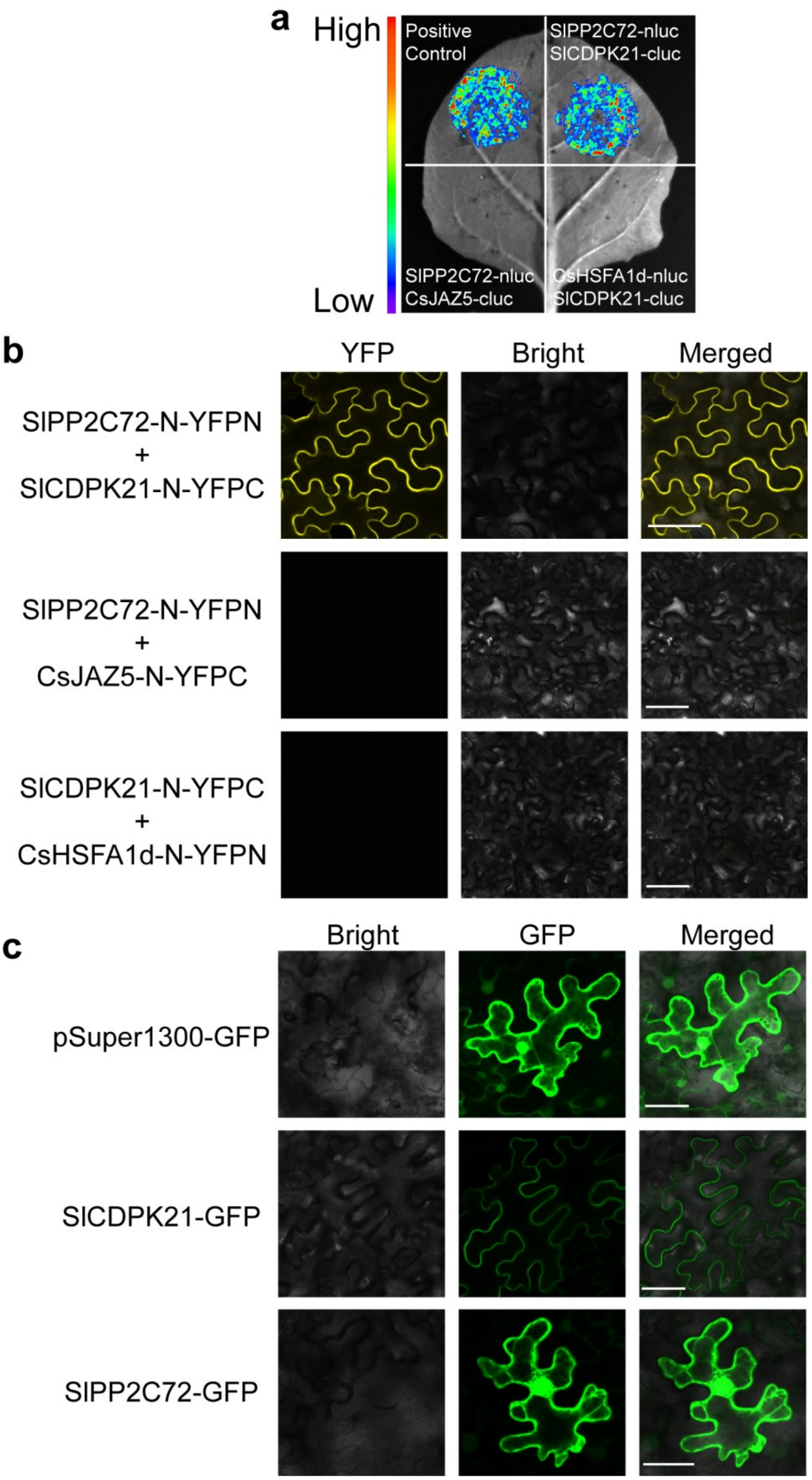

Figure S19. SlCDPK21 interacted with SlPP2C72. (a) BiFC assay showed the interaction between SlCDPK21 and SlPP2C72 occurred at the plasma membrane.

SlCDPK21-N-YFPC and SlPP2C72-N-YFPN proteins were transiently co-expressed in *N. benthamiana*. CsHSFA1d-N-YFPC and CsJAZ5-N-YFPN were used as negative controls. Scale bar, 50 µm. **(b)** LCI assay showed the interaction between SlCDPK21 and SlPP2C72. Constructs carrying SlPP2C72-cluc and SlCDPK21-nluc were co-expressed in *N. benthamiana* leaves for 3 d. SlSTOP1-cluc and SlSZP1-nluc were used as positive control. **(c)** Subcellular location of SlPP2C72 and SlCDPK21. SlPP2C72-GFP and SlCDPK21-GFP was transiently expressed in *N. benthamiana* leaves respectively. Confocal images were taken at 72 h after infiltrating plasmids. Scale bar, 50 µm. Experiments in (a) to (c) were independently repeated three times with similar results.

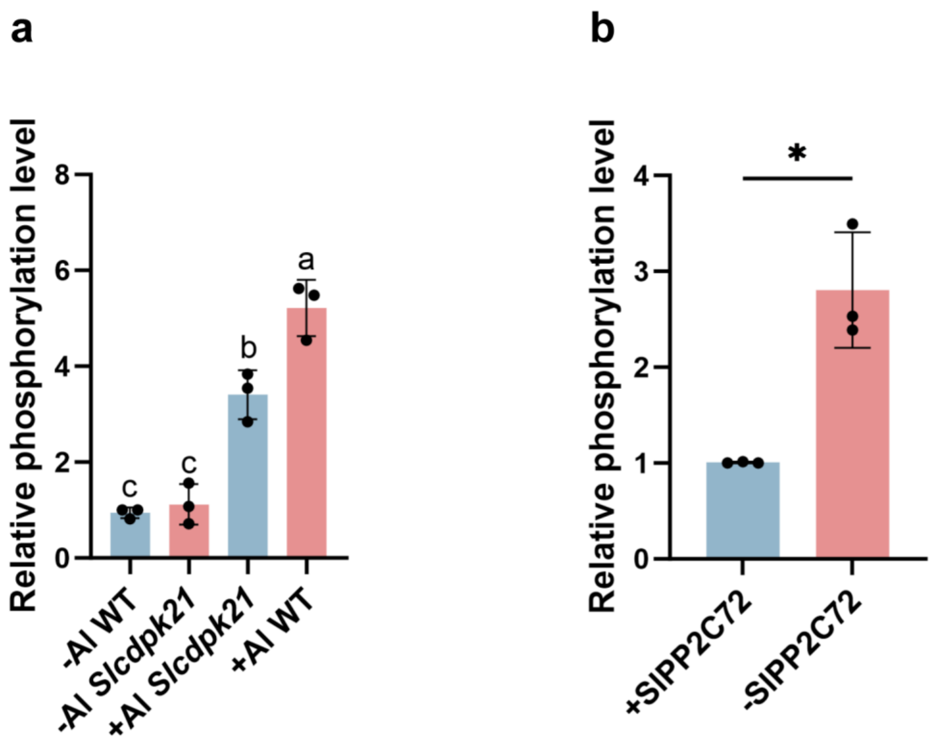

Figure S20. Statistical analysis of signal intensity. The statistical analysis of signal intensity in the western blot assays depicted in Figure 8f and Figure 8i. Data were presented as means ± SD (n=3). Statistical significance was analyzed by **(a)** one-way ANOVA, different lowercase letters indicated significantly different means (Tukey's multiple comparisons test, $p \leq 0.05$) and **(b)** paired two-tailed *t*-test (*$p \leq 0.05$).

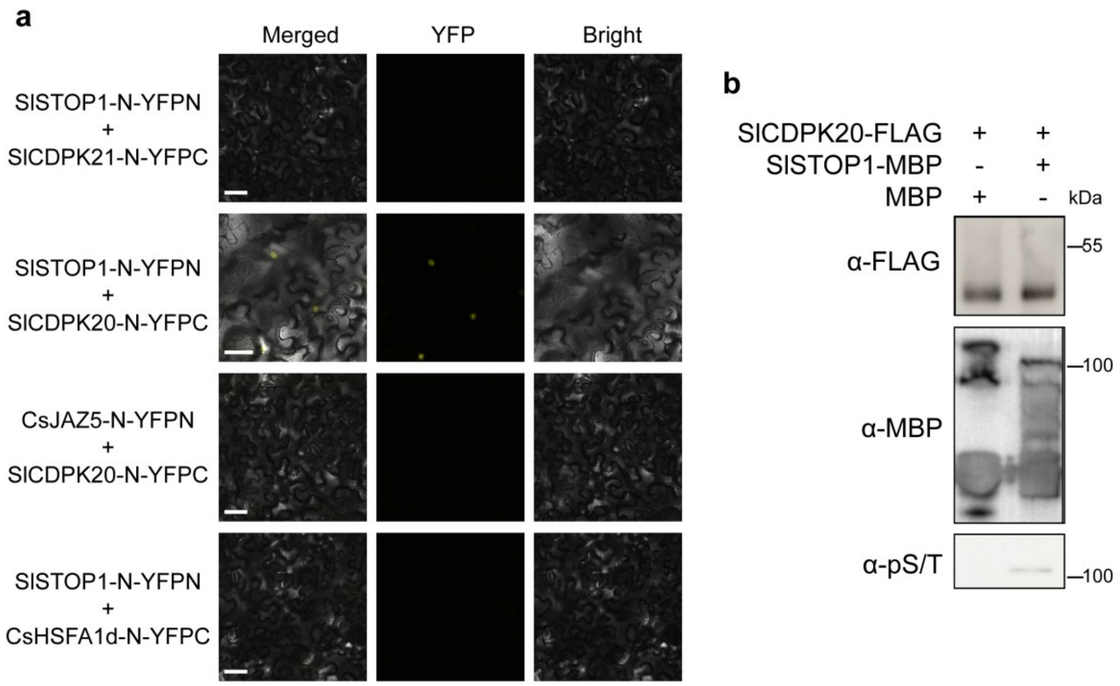

Figure S21. SlCDPK20 interacted and phosphorylated SlSTOP1. (a) BiFC assay showed the interaction between SlCDPK20 and SlSTOP1 occurred at the nuclear. SlCDPK20-N-YFPC and SlSTOP1-N-YFPN proteins were transiently co-expressed in *N. benthamiana*. CsHSFA1d-N-YFPC and CsJAZ5-N-YFPN were used as negative controls. Scale bar, 50 µm. **(b)** *In vitro* kinase assay showed the phosphorylation of SlSTOP1 by SlCDPK20. Recombinant SlSTOP1-MBP was incubated with SlCDPK20-FLAG proteins respectively in protein kinase buffer supplementing ATP. The experiment was independently repeated three times with similar results. Experiments in (a) and (b) were independently repeated three times with similar results.

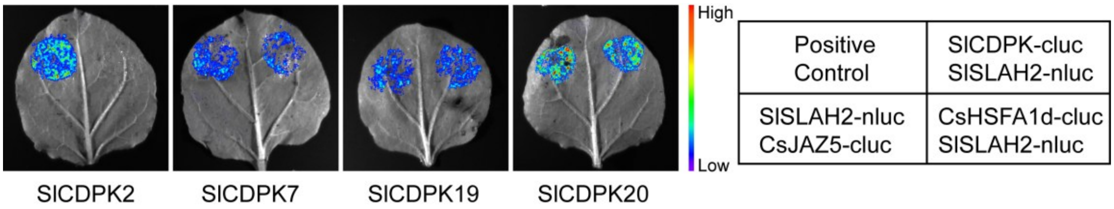

Figure S22. LCI assay showed the interaction between SlCDPKs and SlSLAH2. Constructs carrying SlCDPKs-cluc and SlSLAH2-nluc were co-expressed in *N. benthamiana* leaves for 3 d. CsJAZ5-cluc and CsHSFA1d-nluc were used as positive control. Experiment was independently repeated three times with similar results.

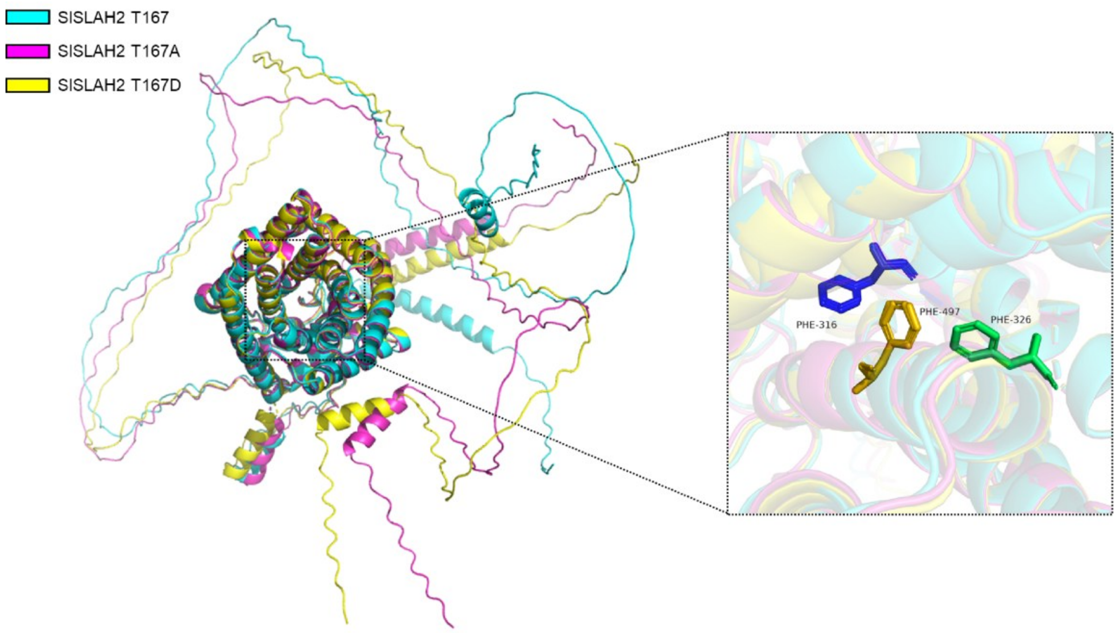

Figure S23. T167 in SlSLAH2 may play vital role in channel activity. Protein structure comparison of SlSLAH2, SlSLAH2^{T167A}, SlSLAH2^{T167D} predicted by Alphafold3.

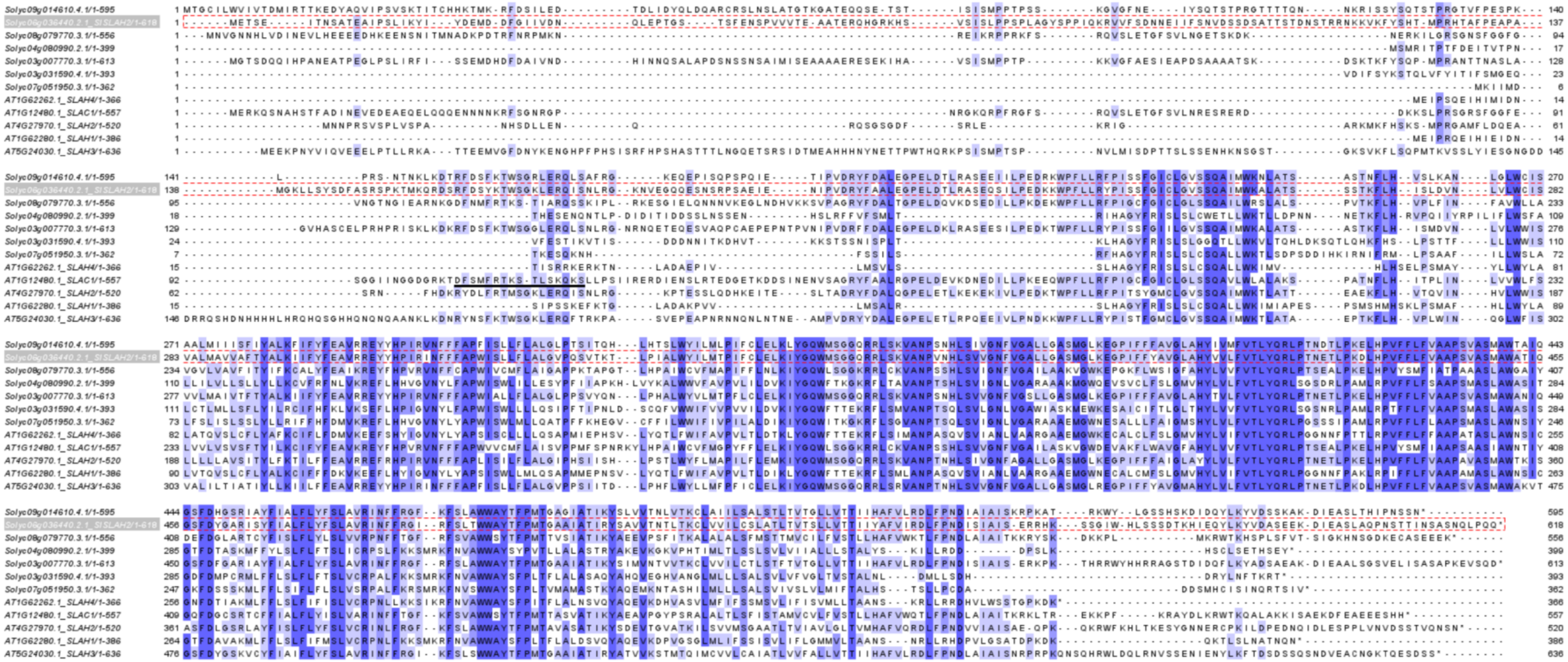

Figure S24. T167 in SlSLAH2 might play vital role in channel activity. Multiple protein sequence alignment of SlSLAC/SLAHs in tomato and ATSLAC/SLAHs in Arabidopsis. The red dashed line indicated the protein sequence of SlSLAH2, while the black solid line denoted the Motif 3 region of AtSLAC1.

Supplemental Table 1. Primers used in this study.

Primers for RT-qPCR

Gene	F	R
SlALMT1	ctaggacacaacccggcc	tatcgacgccaccgacac
SlALMT2	gcgtcggtcgtgtcctta	attcgtggccgatgcgat
SlALMT3	cagtgtcaggctatcgcgt	gcaactcagtacctgccca
SlALMT4	ggtcgcagtgatccgaga	ctcggatcactgcgaccc
SlALMT5	ggcgatggagatgggtcg	aagctctttaaccgcgggt
SlALMT6	tcaatggccttcgcgtct	aaggatagagcactcgcgc
SlALMT7	ggctggaaataggactggag	tgcaaatcttcaccagccca
SlALMT8	ccaatttggtcaggagaaga	ttgctaagccatcgaacttggc
SlALMT9	aacagggctttggggacatt	cacaatcacaacctcctgcc
SlALMT10	tcattggtgtccgtttctggt	tgaagttgttgaccagccca
SlALMT11	aggacagtctggtgcact	tccaatcaagcaacgcgg
SlALMT12	gggagcctgctcatggac	gcaacaggcacaactacgc
SlALMT13	tcgcaggtgctacactaagc	aggcaccaccaatttcacca
SlALMT14	acgcaaccgtcttcctcc	cggtggtggtgaggatcg
SlALMT15	gaatggcaaccttgctagct	tggctccacaactttaccact
SlALMT16	tgtggtgcttgataaggcga	tcttcaccggcccatacc
SlSLAH4	tttgtggctgcacctagc	ctgcccttgctccaacca
SlSLAH1-2	tggattacaaagggcaagcg	tgcagaggctaaggcaagg
SlSLAH1	ggctaaccctacgagccatt	ttgatgcccaagccaagcta
SlSLAH3-2	gtgcacggatcgcctact	tggcaatagcagctccgg
SlSLAH3	agcactcggacttccaacat	cgttgtcctccagacatcca
SlSLAH2	ctgcgcggaaagaatgtgg	ctggtccctccaacgcag
SlWRKY37	ctgccccttcttaccctca	aatgcccgcgaaggatgt
SlPP2C67	ggcctgccttctgaagca	tcgcgacatggccaatcc
SlCDPK21	aaccaccacaaccccagc	ggcggtggctgatatgct

Primers for CRISPR/Cas9

Gene	F	R
SlSLAH2	BsF:atatatggtctcgattgacgatgaaatggatgacttgtt<br>F0:tgacgatgaaatggatgacttgttttagagctagaaatagc	R0:aactgctgtcgttcagtagcagcaatctcttagtcgactctac<br>BsR:attattggtctcgaaactgctgtcgttcagtagcagcaa
SlCDPK21	BsF:atatatggtctcgattgtatcagcctcagccccagcgtt<br>F0:tgtatcagcctcagccccagcgttttagagctagaaatagc	R0:aacggctgtggcggtggctgataaatctcttagtcgactctac<br>BsR:attattggtctcgaaacggctgtggcggtggctgataaa
SlPP2C72	BsF:atatatggtctcgattgtagaaaatggagtttttgggtt<br>F0:tgtagaaaatggagtttttgggttttagagctagaaatagc	r0:aactcgaaaactccaccaaaaacaatctcttagtcgactctac<br>BsR:attattggtctcgaaactcgaaaactccaccaaaaacaa

Primers for Vector Construction

Gene	F	R
SlSLAH2-1305K	tatgaccatgattacgaattcccaaatattgctttcgcaagtta	caggtcgactctagaggatccttgttgaggaagttggtttgag
SlCDPK21-1305K	tatgaccatgattacgaattctttgcttctcgaatgggag	atggtctttgtagtcaagcttgaaaagcttttgttgtggttgt
SlPP2C72-1305K	tatgaccatgattacgaatttcatttccaaacccttggg	caggtcgactctagaggatccttgcaagaacaagcagatga
SlWRKY37-AD424	caaaaaaagagatcgaattcatgggtagaagtaagccaaca	tgcaggtcgacggatccctaagaggagggatctacaagg
PRO-LACZi	atgatgaattgaaaagcttgtaactttatcatataagttgcga	gacagatccccgggtaccaccgaaaatagcaggtacaagct
SlWRKY37-62SK	ggtggcggccgctctagaatgggtagaagtaagccaaca	agcccgggggatccctaagaggagggatctacaagg
PRO-LUC	tagggcgaattgggtacctttatcatataagttgcgactcg	attcgatatcaagcttgaaaatagcaggtacaagct
SlWRKY37-P5X	atcgtcgacggatccatgggtagaagtaagccaaca	gcagggaattcggatccctaagaggagggatctacaagg
SlSLAH2-N-MBP	atcgtcgacggatccatggaaacaagtgaaataactaattct	gcagggaattcggatccaaggaaaggccatttcttgtcc
SlCDPK21-GFP-GST	cgtggatccccggaattcatgggtggttgttttagcaag	acgatgcggccgctcgaggaaaagcttttgttgtggttgt
SLSLAH2-1300	atttaaatactagtatggaaacaagtgaaataactaattctgc	ccttgctcaccatggtaccttgttgaggaagttggtttgaggc
SLPP2C72-1300	tcgacatttaaatactagtatgggtatctgtggactcg	gcccttgctcaccatggtaccttgcaagaacaagcagatga
SLCDPK21-1300	ggtcgacatttaaatactagtatgggtggttgttttagcaag	gcccttgctcaccatggtaccgaaaagcttttgttgtggttgt
SLSLAH2-	catggccggtaccatggaaacaagtgaaat	tcgaacctgcaggtcgacttgttgaggaagtt

NN	aactaattctgc	ggtttgaggc
SLPP2C72-NN	tctatatcatggccggtaccatgggtatctgtggactcg	actcgaacctgcaggtcgacttgcaagaacaagcagatga
SLPP2C72-NC	gctgtacaagggtaccatgggtatctgtggactcg	aacctgcaggtcgacttgcaagaacaagcagatga
SLCDPK21-NC	gctgtacaagggtaccatgggtggttgttttagcaag	aacctgcaggtcgacgaaaagcttttgttgtggttgt
SLSLAH2-NLUC	gtacccgggatccatggaaacaagtgaaataactaattctgc	cgagatctggtcgacttgttgaggaagttggtttgaggc
SLPP2C72-CLUC	cgtcccggggcggtaccatgggtatctgtggactcg	aagctctgcaggtcgacttgcaagaacaagcagatga
SLCDPK21-CLUC	cgtcccggggcggtaccatgggtggttgttttagcaag	aagctctgcaggtcgacgaaaagcttttgttgtggttgt
SLCDPK21-NLUC	cggtacccgggatccatgggtggttgttttagcaag	cgagatctggtcgacgaaaagcttttgttgtggttgt
SLPP2C72-1305	ttcgagctcggtaccatgggtatctgtggactcg	tgcctgcaggtcgacttgcaagaacaagcagatga
SLCDPK21-1305	acggaattcgagctcggtaccatgggtggttgttttagcaag	tgcctgcaggtcgacgaaaagcttttgttgtggttgt
SLSLAH2-1305	ttcgagctcggtaccatggaaacaagtgaaataactaattct	cttgcatgcctgcaggtcgacttgttgaggaagttggtttgag
PKK223-3-SLSLAH2	caggaaacagaattcatggaaacaagtgaaataactaattct	aaacagaagctttcattgttgaggaagttggtttg
SLSLAH2-PGEMHE	ccggggatccgaattcatggaaacaagtgaaataactaattc	ggtaaccagatcaagctttcattgttgaggaagttggtttg
