# Supplementary material for: SlSLAH2 mediates malate exudation and contributes to aluminum tolerance
Source: Nat Commun. 2026 Apr 10;17:5040. doi: 10.1038/s41467-026-71651-1 (PMC13243466; doi:10.1038/s41467-026-71651-1)
Supplement: Supplementary file 2 — Reporting Summary [file 41467_2026_71651_MOESM2_ESM.pdf]

nature portfolio

Corresponding author(s): Yang-Dong Guo

Last updated by author(s): Mar 18, 2026

Reporting Summary

Nature Portfolio wishes to improve the reproducibility of the work that we publish. This form provides structure for consistency and transparency in reporting. For further information on Nature Portfolio policies, see our Editorial Policies and the Editorial Policy Checklist.

Statistics

For all statistical analyses, confirm that the following items are present in the figure legend, table legend, main text, or Methods section.

n/a	Confirmed	
☐	☒	The exact sample size (*n*) for each experimental group/condition, given as a discrete number and unit of measurement
☐	☒	A statement on whether measurements were taken from distinct samples or whether the same sample was measured repeatedly
☐	☒	The statistical test(s) used AND whether they are one- or two-sided<br>*Only common tests should be described solely by name; describe more complex techniques in the Methods section.*
☒	☐	A description of all covariates tested
☒	☐	A description of any assumptions or corrections, such as tests of normality and adjustment for multiple comparisons
☐	☒	A full description of the statistical parameters including central tendency (e.g. means) or other basic estimates (e.g. regression coefficient) AND variation (e.g. standard deviation) or associated estimates of uncertainty (e.g. confidence intervals)
☐	☒	For null hypothesis testing, the test statistic (e.g. *F*, *t*, *r*) with confidence intervals, effect sizes, degrees of freedom and *P* value noted<br>*Give P values as exact values whenever suitable.*
☒	☐	For Bayesian analysis, information on the choice of priors and Markov chain Monte Carlo settings
☒	☐	For hierarchical and complex designs, identification of the appropriate level for tests and full reporting of outcomes
☒	☐	Estimates of effect sizes (e.g. Cohen's *d*, Pearson's *r*), indicating how they were calculated

Our web collection on statistics for biologists contains articles on many of the points above.

Software and code

Policy information about availability of computer code

Data collection	Fluorescent images were taken by laser scanning confocal microscope Leica SP8. Gene expression was measured with qRT-PCR reactions conducted with QuantStudio6 instrument (Thermo Fisher Scientific).
Data analysis	Statistical analyses were conducted with GraphPad Prism 10.

For manuscripts utilizing custom algorithms or software that are central to the research but not yet described in published literature, software must be made available to editors and reviewers. We strongly encourage code deposition in a community repository (e.g. GitHub). See the Nature Portfolio guidelines for submitting code & software for further information.

Data

Policy information about availability of data

All manuscripts must include a data availability statement. This statement should provide the following information, where applicable:

- Accession codes, unique identifiers, or web links for publicly available datasets
- A description of any restrictions on data availability
- For clinical datasets or third party data, please ensure that the statement adheres to our policy

The authors declare that the data supporting the findings of this study are available within the paper and its supplementary information files. Source data are provided with this paper.

Research involving human participants, their data, or biological material

Policy information about studies with human participants or human data. See also policy information about sex, gender (identity/presentation), and sexual orientation and race, ethnicity and racism.

Reporting on sex and gender	N/A
Reporting on race, ethnicity, or other socially relevant groupings	N/A
Population characteristics	N/A
Recruitment	N/A
Ethics oversight	N/A

Note that full information on the approval of the study protocol must also be provided in the manuscript.

Field-specific reporting

Please select the one below that is the best fit for your research. If you are not sure, read the appropriate sections before making your selection.

☒ Life sciences ☐ Behavioural & social sciences ☐ Ecological, evolutionary & environmental sciences

For a reference copy of the document with all sections, see nature.com/documents/nr-reporting-summary-flat.pdf

Life sciences study design

All studies must disclose on these points even when the disclosure is negative.

Sample size	The sample sizes used for all experiments provide sufficient resolving power to show statistical significance due to homogeneity of plant individuals.
Data exclusions	Samples are only excluded after confirming as outliers after statistic analysis.
Replication	All the experiments were performed three times.
Randomization	All plants are grown in the same growth medium in the same growth chamber followed a completely randomized block design.
Blinding	No blinding is performed. All plants were subjected to identical growth conditions in each experiment.

Reporting for specific materials, systems and methods

We require information from authors about some types of materials, experimental systems and methods used in many studies. Here, indicate whether each material, system or method listed is relevant to your study. If you are not sure if a list item applies to your research, read the appropriate section before selecting a response.

Materials & experimental systems

n/a	Involved in the study	
☐	☒	Antibodies
☐	☒	Eukaryotic cell lines
☒	☐	Palaeontology and archaeology
☒	☐	Animals and other organisms
☒	☐	Clinical data
☒	☐	Dual use research of concern
☐	☒	Plants

Methods

n/a	Involved in the study	
☒	☐	ChIP-seq
☒	☐	Flow cytometry
☒	☐	MRI-based neuroimaging

Antibodies

Antibodies used	α-FLAG (ab49763, Abcam, Cambridge, UK), α-GFP antibody (SLAB3000, Smart-lifesciences, Changzhou, China), α-Pan Phospho-ser/thr (α-pS/T) antibody (Ab117253, Abmart, Shanghai, China), α-MBP antibody (HT701-01, TransGen Biotech, Beijing, China), α-GST antibody (CW0084M, CWBIO, Jiangsu, China) and α-ACTIN antibody (AC038, ABclonal Technology, WuHan, China).

Validation	α-pS/T (rabbit), α-MBP (mouse), α-FLAG (mouse), α-GST (mouse) and α-ACTIN (rabbit). All the antibodies validation information can be found on the manufacturer's website.

Eukaryotic cell lines

Policy information about cell lines and Sex and Gender in Research

Cell line source(s)	E. coli strain BL21 (DE3) , Agrobacterium strain GV3101, Agrobacterium strain GV3101-p19-psoup, E. coli strain CBT315, Yeast strain YM4271
Authentication	N/A
Mycoplasma contamination	N/A
Commonly misidentified lines (See ICLAC register)	N/A

Plants

Seed stocks	The tomato (Solanum lycopersicum L.) cultivar, MicroTom, Ailsa Craig, Hacienda Rosario, Moyobamba, Rowpac and TS-261 were used in this study and MicroTom was used as wild-type (WT), the seeds are self-propagated by our laboratory or given by other team.
Novel plant genotypes	Transgenic plants were modified via Agrobacterium tumefaciens (GV3101)-mediated cotyledon explant transformation. The knockout mutants were generated by CRISPR/Cas9 technology, potential guide sequences were predicted by the website CRISPOR (http://crispor.tefor.net/). The editing type were described in supplemental figures.
Authentication	No off-target gene editing has been detected in knockout lines.
