# Supplementary material for: SlSLAH2 mediates malate exudation and contributes to aluminum tolerance
Source: Nat Commun. 2026 Apr 10;17:5040. doi: 10.1038/s41467-026-71651-1 (PMC13243466; doi:10.1038/s41467-026-71651-1)
Supplement: Supplementary file 3 — Transparent Peer Review file [file 41467_2026_71651_MOESM3_ESM.pdf]

nature portfolio

Peer Review File

SlSLAH2 mediates malate exudation and contributes to Aluminum tolerance

Corresponding Author: Professor Na Zhang

Parts of this Peer Review File have been redacted as indicated to remove third-party material.

This file contains all reviewer reports in order by version, followed by all author rebuttals in order by version.

Version 0:

Reviewer comments:

Reviewer #1

(Remarks to the Author)
This study reports that the tomato SlSLAH2 is involved in aluminum-induced root malate secretion, contributing to aluminum stress resistance, and further elucidates the regulatory mechanisms governing SlSLAH2. At the transcriptional level, SlWRKY37 activates SlSLAH2 expression. At the post-transcriptional level, aluminum stress activates SlCDPK21, which directly phosphorylates SlSLAH2 and concurrently phosphorylates SlPP2C67 to inhibit its phosphatase activity, thereby reducing dephosphorylation of SlSLAH2. These findings offer valuable insights into aluminum resistance mechanisms in tomatoes. However, the following issues require attention from the authors:

1. It is well established that members of the ALMT family are responsible for aluminum-activated malate secretion, with wheat ALMT1 being the first identified aluminum-activated malate transporter. Previous studies have also shown that aluminum stress induces the expression of SlALMT9 (Ye et al., The Plant Cell, 2017, 29: 2249-2268), which contributes to aluminum tolerance in tomatoes. Here, the authors suggest that ALMT family members are not involved in Al-induced root malate secretion in Micro-Tom, and instead propose a role for SlSLAH2. The authors should provide more compelling experimental evidence, including:
(1) Line 131: Expression of SlALMT genes across different tomato varieties. Are the selected varieties representative? For instance, the discussion mentions that SlALMT9 (in tomato variety TS40) was significantly induced by Al stress in a 2017 study by Ye et al. published in The Plant Cell. This suggests substantial varietal differences in SlALMT expression. Please supplement with SlALMT expression data from at least TS40 and AC varieties. Additionally, among the varieties examined, SlSLAH2 was consistently induced while SlALMT expression was suppressed. Is there a potential relationship between these two trends?
(2) The malate transport capability of SlSLAH2 has only been indirectly demonstrated in yeast. Please provide electrophysiological evidence using Xenopus oocytes. Furthermore, its homolog in Arabidopsis, SLAC1, has been reported to exhibit high selectivity for nitrate, with weaker affinity for chloride and malate. Given the high conservation of transmembrane domains between SlSLAH2 and SLAC1, please clarify whether SlSLAH2 has similar ion selectivity— specifically, whether it primarily transports malate or other anions.

2. If STOP1 does not regulate SlSLAH2, is malate secretion impaired in stop1 mutants? If so, how does the impairment compare to that in SlSLAH2 mutants? Could this support the conclusion that SlSLAH2 is the primary aluminum-induced malate transporter in tomato? Additionally, SlCDPK21 shows high homology with AtCPK21/23, which acts as a calcium sensor and phosphorylates STOP1. Does SlCDPK21 phosphorylate STOP1 in tomato? Please provide additional evidence and discuss in the Discussion section the relationship between SlCDPK21-mediated phosphorylation of STOP1 and its phosphorylation of SlSLAH2. How do these two pathways interact and function synergistically within the acid-aluminum stress regulatory network?

3. In Fig. 6a, the gene expression annotation for SlPP2C67 appears to be incorrect; please verify. Moreover, SlPP2C67 is localized to both the plasma membrane and the nucleus. Why does it exit the nucleus upon aluminum stress to be phosphorylated by SlCDPK21, thereby inhibiting its phosphatase activity? Wouldn't direct regulation at the plasma membrane be more efficient?

4. Although SlCDPK21 interacts with and inhibits SlPP2C67 phosphatase activity, SlPP2C67 does not directly interact with SlSLAH2 yet can dephosphorylate it. What is the mechanistic basis for this? Please discuss and provide in vivo evidence— for example, the phosphorylation status of SlSLAH2 in SlPP2C67 mutants.

5. Additional evidence is needed to demonstrate that SlCDPK21 phosphorylates SlPP2C67 to inhibit its phosphatase activity, particularly in vivo. For instance, please assess SlPP2C67 phosphatase activity in SlCDPK21 mutants.

6. Please provide data on malate secretion levels in wrky37 mutants. Additionally, include acid-aluminum stress phenotypes and malate secretion data for SlCDPK21 and SlPP2C67 mutants/overexpression lines.

Minor Concerns:
1. Aluminum-induced organic acid secretion occurs primarily in the root tip. Please supplement with spatial expression patterns of each gene involved.
2. Please specify the age of seedlings used in the experiments.

Reviewer #2

(Remarks to the Author)
The exudation of organic acids, such as malate and citrate, mediated by ALMTs and MATEs, is regarded as a major mechanism of aluminum (Al) resistance in plants. Studies have found that plants may utilize either citrate or malate, or both to chelate external Al. In this manuscript, Dong et al. identified SlSLAH2 is associated with aluminum toxicity in tomato. The authors intend to uncover the molecular mechanisms underlying how Al regulates SlSLAH2 activity, thereby conferring Al resistance. The manuscript provides a substantial amount of data; however, I still have several questions.
1.Figure 1: It is curious to note that nearly all of the genes from the ALMT family are reduced by Al (Figure 1). Although the authors discussed the possible role of SlALMT9 in Al toxicity as located in the vacuolar membrane, its overexpression significantly enhances malate exudation by Al (Ye et al., 2017). Additionally, SlSTOP1 plays a major role in Al-induced organic acid exudation; but it cannot bind with the promoter of SlSLAH2. Therefore, SlSLAH2 may not play a predominant role in Al-induced malate exudation. The authors should interpret in the discussion section.
2.Figure 2: Data from E. coli to confirm the role of SlSLAH2 in malate transport (possible influx or adsorption) is insufficient. I would suggest that the authors use Xenopus oocytes to determine whether this protein mediates malate efflux. Furthermore, root elongation and Al content should also be included here.
3.Figure 4b: Root length and malate concentrations should be presented as two separate figures.
4.Figure 6: Protein phosphorylation and dephosphorylation are dependent on the interactions of kinases and phosphatases with their targets. How SlPP2C67 could dephosphorylate SlCDPK21 without direct interaction?
5.Figure 7: Why the phosphatase activities of SlPP2C67 also significantly increased in the control treatments (CK, GFP) as shown in Figure 7a and b? Does the measurement system is unstable? For Figure 7g seems to lack an important control that the phosphorylation levels of SlPP2C67 should also be invested in SlCDPK21 mutant plants with or without Al treatments.
6.Considering the significant roles of SlCDPK21 and SlPP2C67 in regulating SlSLAH2 activity, I recommend that the authors further investigate their involvement in Al-induced malate exudation and Al resistance by utilizing mutant and overexpressing transgenic plants.
7.Line 288, phosphorylation should be revised as dephosphorylation.

Version 1:

Reviewer comments:

Reviewer #1

(Remarks to the Author)
In this revised manuscript, the authors have adequately addressed my previous concerns, particularly by incorporating electrophysiological assays for SlSLAH2. This study elucidates a novel pathway through which tomato roots secrete malate to alleviate aluminum toxicity. Using the Micro-Tom tomato cultivar, the authors characterized the function of SlSLAH2 in aluminum-induced malate exudation. Based on promoter cis-element analysis and RNA-seq data mining, they demonstrated that SlWRKY37—but not SlSTOP1—regulates SlSLAH2 expression under Al stress. Furthermore, they showed that SlCDPK21 can interact with and phosphorylate SlSLAH2. In addition to phosphorylation, SLAH2 undergoes dephosphorylation to modulate its activity. The study also revealed that SlPP2C72 indirectly mediates the dephosphorylation of SlSLAH2, thereby negatively regulating malate efflux.

The following issues remain to be clarified:

The authors did not describe the method used for malate quantification.

The evidence supporting the plasma membrane localization of SlSLAH2 remains insufficiently convincing.

Reviewer #2

(Remarks to the Author)
The revised manuscript shows improvement, with most of my previous questions addressed. However, I still have several concerns:

1.The data still lack solid genetic evidence. For instance, the authors identified Thr167 as the phosphorylation site targeted by CDPK21. To conclusively demonstrate that SLAH2 phosphorylation by CDPK21 is necessary for Al-induced malate exudation, complementary experiments expressing the phospho-deficient (SLAH2T167A) and phospho-mi metic (SLAH2T167D) variants in the slah2, cdpk21, pp2c72 mutant backgrounds should be performed. Additionally, does PP2C72 dephosphorylate SLAH2 at the same Thr167 site?

2.Why do the aluminum treatment concentrations vary across assays measuring root elongation, malate secretion, gene expression, and protein phosphorylation? The root elongation and malate exudation used 30 μM Al, whereas phosphorylation experiments used 90 μM Al. Is 30 μM Al insufficient to regulate protein phosphorylation levels? The concentrations of aluminum treatment should be standardized to ensure the comparability of experimental results.

3.The response regarding PP2C phosphatase activity assays remains unsatisfactory. The current figure may mislead readers: the increasing A405 absorbance over time reflects the accumulation of reaction products rather than an increase in enzyme activity. Therefore, I recommend presenting these data as enzyme activity rates (e.g., nmol substrate converted per minute per mg protein) rather than raw A405 values.

4.How many biological replicates were performed for the western blot experiments? The blot images should include visible protein markers and the entire membrane. Without full membrane images, it is difficult to assess the specificity and reliability of the results. Since PP2C72 interacts with CDPK21, the authors primarily focus on how CDPK21 phosphorylates PP2C72. However, does PP2C72 also dephosphorylate CDPK21?

5.The methods section lacks a description of the electrophysiological analysis of Xenopus oocytes.

6.The current title may be misleading, as it suggests that both the kinase and phosphatase promote malate exudation and enhance aluminum tolerance, whereas in reality, they perform opposing functions. Consider revising the title to more accurately reflect their antagonistic roles.

Version 2:

Reviewer comments:

Reviewer #1

(Remarks to the Author)
I am satisfied that my concerns have been fully addressed by the authors. I have no additional comments or questions at this time.

Reviewer #2

(Remarks to the Author)
The manuscript has been substantially improved and is now suitable for publication.

Open Access This Peer Review File is licensed under a Creative Commons Attribution 4.0 International License, which permits use, sharing, adaptation, distribution and reproduction in any medium or format, as long as you give appropriate credit to the original author(s) and the source, provide a link to the Creative Commons license, and indicate if changes were made.
In cases where reviewers are anonymous, credit should be given to 'Anonymous Referee' and the source.
The images or other third party material in this Peer Review File are included in the article's Creative Commons license, unless indicated otherwise in a credit line to the material. If material is not included in the article's Creative Commons license and your intended use is not permitted by statutory regulation or exceeds the permitted use, you will need to obtain permission directly from the copyright holder.
To view a copy of this license, visit https://creativecommons.org/licenses/by/4.0/

Reviewer #1:

This study reports that the tomato SlSLAH2 is involved in aluminum-induced root malate secretion, contributing to aluminum stress resistance, and further elucidates the regulatory mechanisms governing SlSLAH2. At the transcriptional level, SlWRKY37 activates SlSLAH2 expression. At the post-transcriptional level, aluminum stress activates SlCDPK21, which directly phosphorylates SlSLAH2 and concurrently phosphorylates SlPP2C67 to inhibit its phosphatase activity, thereby reducing dephosphorylation of SlSLAH2. These findings offer valuable insights into aluminum resistance mechanisms in tomatoes. However, the following issues require attention from the authors:

Q1. It is well established that members of the ALMT family are responsible for aluminum-activated malate secretion, with wheat ALMT1 being the first identified aluminum-activated malate transporter. Previous studies have also shown that aluminum stress induces the expression of SlALMT9 (Ye et al., The Plant Cell, 2017, 29: 2249-2268), which contributes to aluminum tolerance in tomatoes. Here, the authors suggest that ALMT family members are not involved in Al-induced root malate secretion in Micro-Tom, and instead propose a role for SlSLAH2. The authors should provide more compelling experimental evidence, including: **Q1-1.** Line 131: Expression of SlALMT genes across different tomato varieties. Are the selected varieties representative? For instance, the discussion mentions that SlALMT9 (in tomato variety TS40) was significantly induced by Al stress in a 2017 study by Ye et al. published in The Plant Cell. This suggests substantial varietal differences in SlALMT expression. Please supplement with SlALMT expression data from at least TS40 and AC varieties. Additionally, among the varieties examined, SlSLAH2 was consistently induced while SlALMT expression was suppressed. Is there a potential relationship between these two trends?

A1-1: Thank you very much for raising this insightful and important point. The points you raised regarding the representativeness of tomato varieties and the potential complexity of SlALMT9 in aluminum stress response are crucial for improving our research. We have conducted supplementary experiments and in-depth analyses based on your suggestions. Ye et

al. (2017) demonstrated that tomato varieties can be categorized into low-malate content type and high-malate content types based on differences in *SlALMT9* expression level, with high malate content types exhibiting greater tolerance to aluminum stress due to exuding more malate under aluminum stress. In the tomato variety TS-40 (representative of high-malate types), *SlALMT9* promoter activity is induced under high aluminum conditions (200 μM) (Figure R1). To systematically evaluate the expression patterns of *SlALMT* across varieties, we additionally selected TS-40, AC, TS-135, and TS-261 (high-malate content types) as well as TS-129 and TS-186 (low-malate content types) and detected the expression of *SlALMT* genes in their root tips under 60 μM Al^{3+} conditions (Figure S2). The results showed that in all tested varieties, the expression of *SlALMT* genes was suppressed under aluminum stress. This finding was consistent with our previous observations in MicroTom, indicating that the transcriptional repression of the *SlALMT* genes under early/moderate aluminum stress (60 μM) was a relatively common phenomenon in tomatoes, rather than a characteristic specific to certain varieties. Meanwhile, we analyzed the RNA-seq data of AC tomato treated with Al^{3+} (10 μM) and obtained similar results, *SlALMT* expression was still suppressed by aluminum stress (Figure S1b).

We acknowledge the findings of Ye et al. and believe they are not contradictory to our own results. Instead, they reveal a multi-layered, stage-specific, and finely tuned strategy employed by tomato in response to aluminum stress. Based on our research and that of Ye's results, we propose the following model to explain the potential relationship between SlALMT9 and SlSLAH2. **Early/Moderate Al Stress**: When plant roots perceive Al^{3+} in the soil, they rapidly activate a malate exudation detoxification mechanism primarily mediated by SlSLAH2. *SlSLAH2* is quickly activated at the transcriptional level by SlWRKY37 and modified post-translationally by SlCDPK21 phosphorylation, efficiently secreting malate into the rhizosphere to directly chelate Al^{3+}. At this stage, to prioritize resources for this immediate defense response, the plant may downregulate genes associated with other non-essential metabolic pathways (such as fruit acidity maintenance involving SlALMT9). This could explain the observed suppression of *SlALMT* family genes during this phase. Therefore, the upregulation of *SlSLAH2* and the down-regulation of *SlALMTs* reflect the plant's strategic shift of resources and metabolic flux toward the most effective external detoxification

mechanism during early stress. **Severe Al Stress/Later Stages**: As aluminum stress intensifies, some Al^{3+} may break the external defenses and enter the plant, potentially translocating to the above-ground part of the plant. At this point, the plant activates an internal tolerance mechanism dominated by SlALMT9. As shown by Ye et al., the promoter of *SlALMT9* is activated by high Al^{3+} concentrations. Since SlALMT9 is localized to the vacuolar membrane, its function is not to secrete malate extracellularly but to regulate malate flow between the vacuole and the cytoplasm [OsALMT7 exhibits bidirectional transport properties in rice (Heng et al., 2018)]. Its Arabidopsis homolog, AtALMT9, has been confirmed to be responsible for maintaining vacuolar malate homeostasis. We hypothesize that the induced expression of *SlALMT9* under severe aluminum stress represents a "survival strategy": the plant may actively mobilize malate stored in vacuoles, particularly in sink organs like fruits. The released malate could be used in the cytoplasm to chelate Al^{3+} that have entered the cell, mitigating intracellular toxicity. Alternatively, it might be transported over long distances or via other means to the roots, providing substrate support for the sustained, high-intensity malate exudation in the rhizosphere. We have integrated the above discussion into the "Discussion" section of the revised manuscript (line 439 to 460) to present a more comprehensive view of the complexity of aluminum tolerance mechanisms in tomato.

Persistent induction of *SlSLAH2* coupled to sustained repression of *SlALMT* may point to an energy- and carbon-allocation trade-off—the “survival strategy” of SlALMT9 outlined above. Under Al stress the plant prioritizes its limited resources for the most efficient detoxification route, SlSLAH2. Classic ALMT transporters are costly to express and activate; tomato appears to actively shut down multiple SlALMT branches that are sub-optimal in this species, freeing resources for construction and operation of the high-efficiency SlSLAH2 pathway. Simultaneous activation of functionally similar anion channels could also generate signaling conflicts: parallel malate-exudation systems (ALMT and SLAC/SLAHs) might compete for the same cytosolic second messengers (Ca^{2+} bursts), diluting the core defense signal. By silencing *SlALMT*, the plant focuses SlSLAH2; Al^{3+}-induced Ca^{2+} bursts are funneled exclusively through SlCDPK21 to phosphorylate and activate SlSLAH2 without cross-talk. Later, when continuous malate supply is needed to feed rhizosphere chelation or to chelate Al^{3+} that has already entered the cell, SlALMT genes are activated, delivering malate

from the vacuole or other tissues to the cytosol and root—an internal tolerance mechanism that synergizes with the external detoxification.

Published Data Figure Redacted

Figure R1. Histochemical localization of GUS activity in roots of *SlALMT9*^{HMH} pro::GUS (Ye et al., 2017).

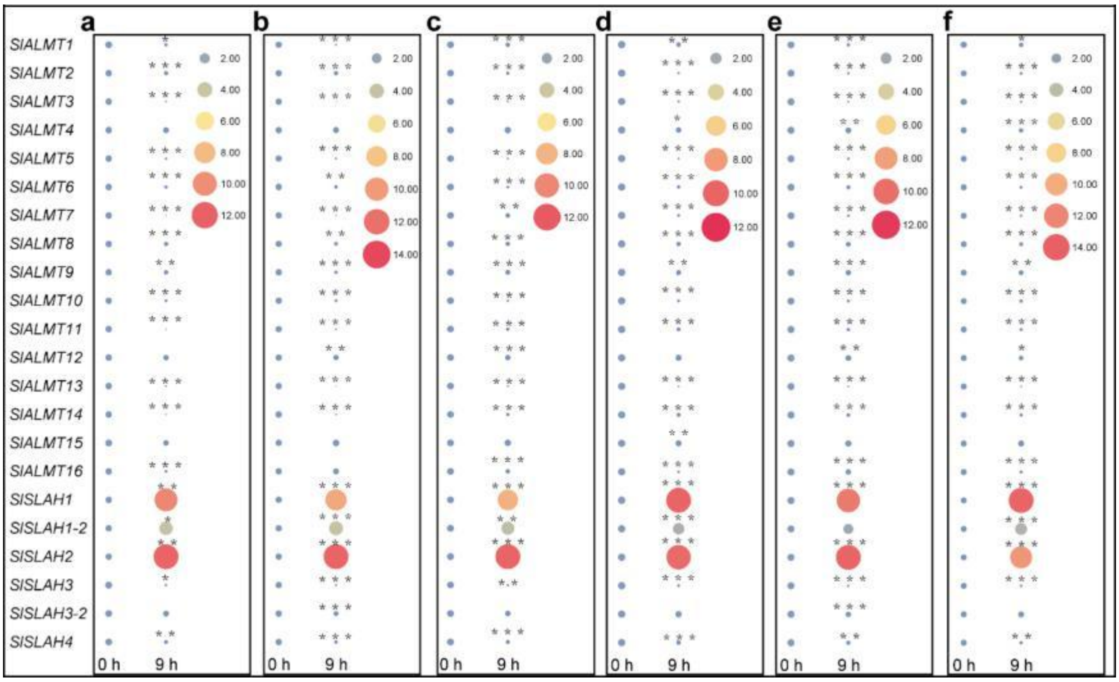

Figure S2. The temporal expression profile of the *SlALMT* and *SlSLAC/SLAHs* genes in tomato under aluminum stress. The expression level of *SlALMT* and *SlSLAC/SLAHs* were detected by RT-qPCR in different tomato cultivars under aluminum stress. (a) Cerise VFNT (TS-40), (b) Ailsa Craig (TS-9), (c) Hacienda Rosario (TS-135), (d) Moyobamba (TS-129) (e) Rowpac (TS-186) (f) TS-261.

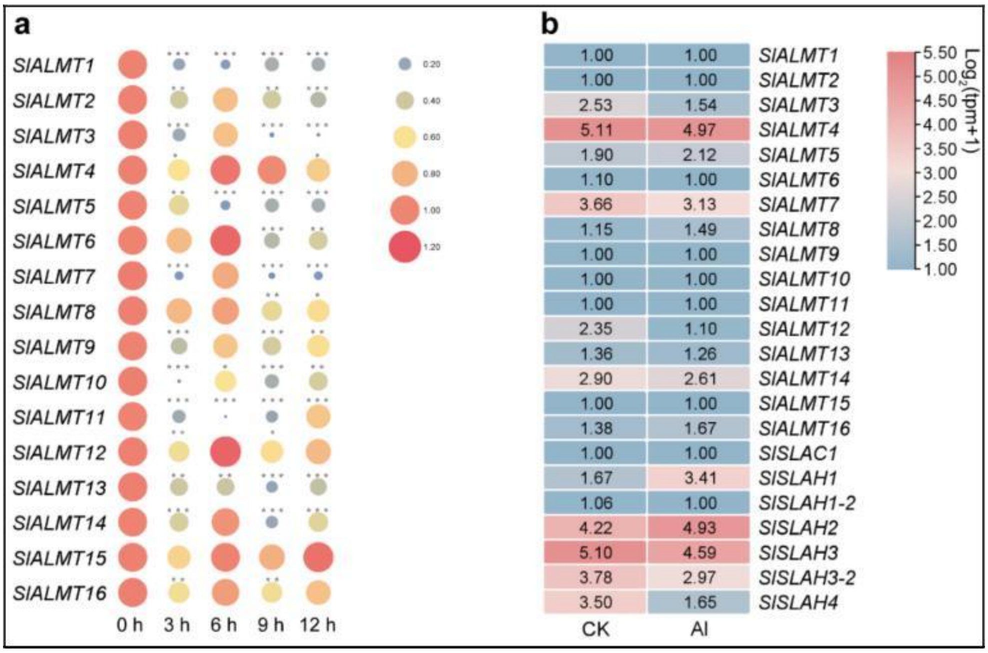

Figure S1. The temporal expression profile of the *SlALMT* genes in tomato under aluminum stress. (a) The expression level of *SlALMT* were detected by RT-qPCR in MicroTom WT under aluminum stress. (b) Heatmap analysis of *SlALMT* gene expression in Ailsa Craig (AC) WT tomato treated with or without $AlCl_3$.

Q1-2. The malate transport capability of SlSLAH2 has only been indirectly demonstrated in yeast. Please provide electrophysiological evidence using Xenopus oocytes. Furthermore, its homolog in Arabidopsis, SLAC1, has been reported to exhibit high selectivity for nitrate, with weaker affinity for chloride and malate. Given the high conservation of transmembrane domains between SlSLAH2 and SLAC1, please clarify whether SlSLAH2 has similar ion selectivity—specifically, whether it primarily transports malate or other anions.

A1-2: Thank you very much for your valuable suggestions. As suggested, we have added patch-clamp experiments to demonstrate that SlSLAH2, as an anion transport channel, can transport not only malate but also nitrate and chloride (Figure 2b and S3). By expressing SlSLAH2 in *Xenopus* oocytes and applying 50 mM solutions containing the corresponding anions, we found that all these anions can activate anion currents of SlSLAH2, with a greater anion current response observed for nitrate.

The reviewer correctly points out the high conservation of transmembrane domains between SlSLAH2 and AtSLAC1, which is known for its high nitrate selectivity. Our new

electrophysiological data confirm that, similar to its homologs, SlSLAH2 does exhibit a significant permeability to nitrate, which appears to be higher than its permeability to malate under the tested conditions. However, we would like to emphasize that the physiological function of an ion channel is determined not solely by its inherent ion selectivity in vitro, but more critically by its expression regulation and physiological context within specific tissues and under specific stress conditions. This principle is well-exemplified by other members of the SLAC/SLAH family. For instance: AtSLAH3, while permeable to nitrate and chloride, is recruited for different physiological roles—mediating nitrate efflux to alleviate ammonium toxicity (Zheng et al., 2015; Xiao et al., 2022), facilitating chloride and nitrate fluxes during pollen tube growth (Gutermuth et al., 2013), releasing nitrate under flooding stress (Lehmann et al., 2021), facilitating chloride transport by forming complex with AtSLAH1 (Cubero-Font et al., 2016). Thus, the specific anion transported is dictated by the physiological signal and cellular context, not merely by the channel's innate preference. *SlSLAH2* was specifically and highly expressed in roots (Figure 2d) and was strongly and rapidly upregulated by aluminum stress (Figures 1c, d), coinciding with the demand for malate exudation, while most SlALMT genes were repressed. The *Slslah2* mutants show a significant reduction in Al^{3+}-induced malate exudation (Figures 2e), providing direct genetic proof of its physiological role in malate transport under Al stress. Therefore, we propose that although SlSLAH2 possesses a high permeability to nitrate, the Al^{3+} stress signaling cascade (involving transcriptional activation by SlWRKY37 and phosphorylation by SlCDPK21) specifically recruits and activates this channel in the root apex to facilitate malate efflux. In the context of aluminum stress, malate is the primary anion required for external detoxification, and the plant's regulatory machinery ensures that SlSLAH2 is deployed for this purpose.

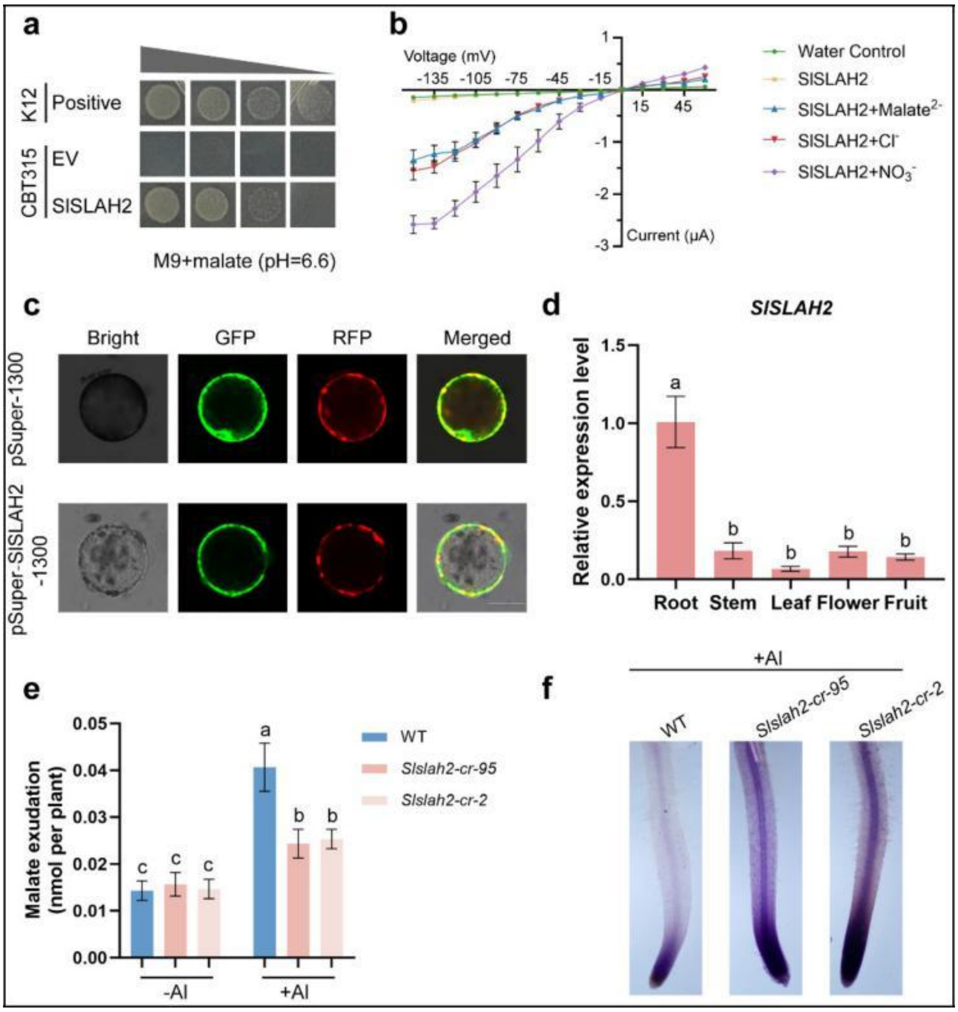

Figure 2. SlSLAH2 was a plasma membrane-localized malate transporter. (a) SlSLAH2 partially restored the growth defect of dicarboxylate-uptake-deficient *E. coli* mutant CBT315. (b) SlSLAH2 was an anion channel. (c) Subcellular location of SlSLAH2. (d) Tissue-specific expression analysis of *SlSLAH2* in tomato. (e) Malate exudation in roots of MicroTom WT lines and *Slslah2* mutant lines. (f) Hematoxylin staining of root Al content.

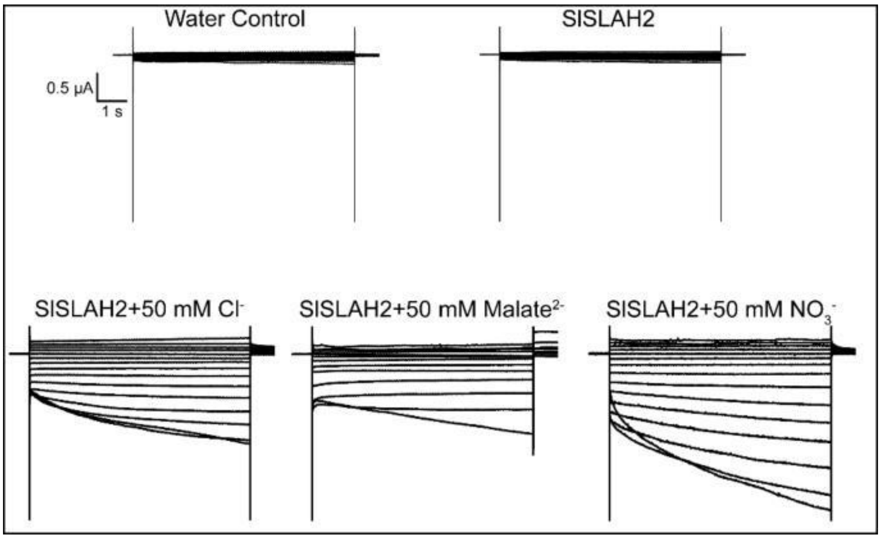

Figure S3. **SlSLAH2 was an anion transporter.** Typical whole-cell current traces.

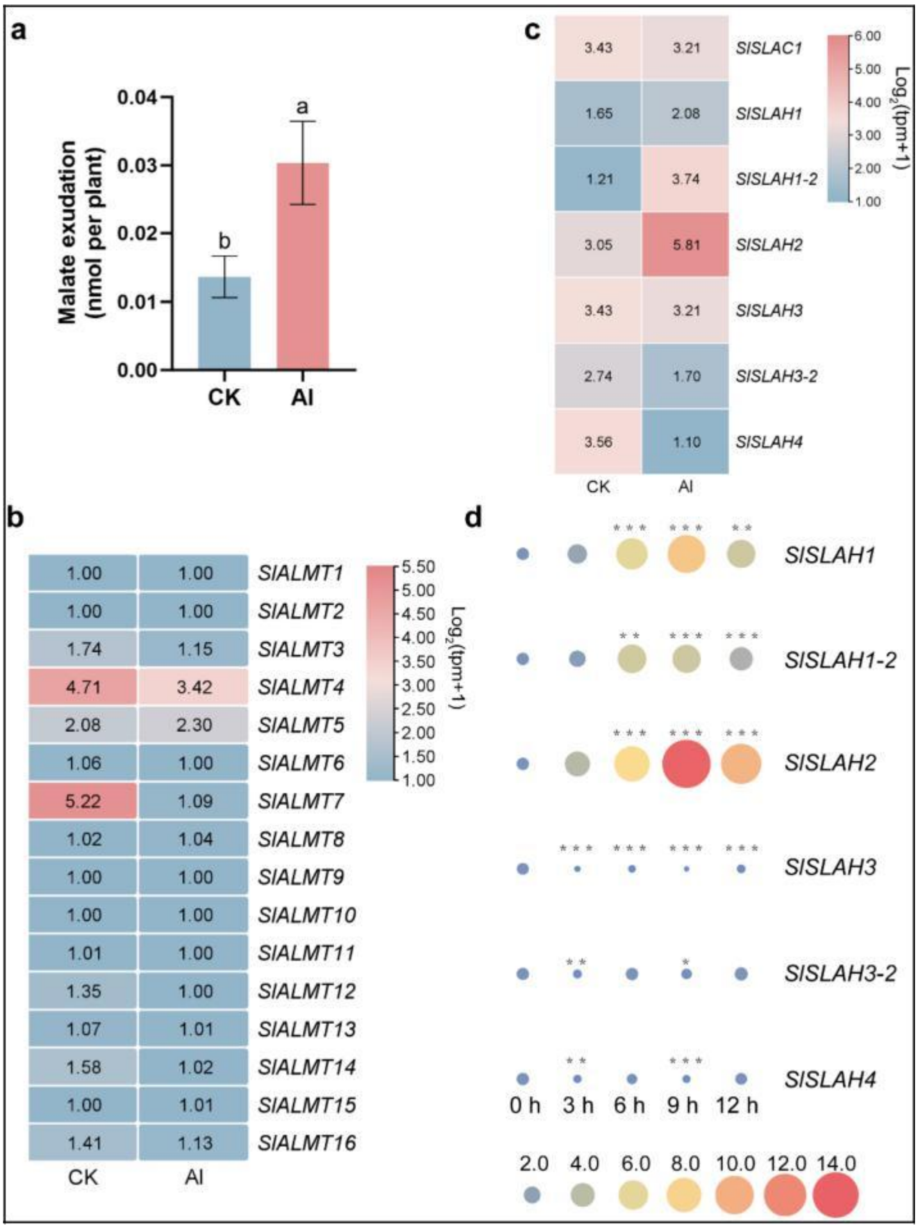

Figure 1. Al^{3+} induced expressions of *SlSLAC*/*SLAHs* instead of *SlALMT* in tomato. (a) Malate exudation in roots of MicroTom WT. Heatmap analysis of *SlALMT* (b) and *SlSLAC*/*SLAHs* (c) gene expression in MicroTom WT treated with or without aluminum stress. (d) The expression level of *SlSLAC*/*SLAHs* were detected by RT-qPCR under aluminum stress.

Q2-1. If STOP1 does not regulate SlSLAH2, is malate secretion impaired in stop1 mutants? If so, how does the impairment compare to that in SlSLAH2 mutants? Could this support the conclusion that SlSLAH2 is the primary aluminum-induced malate transporter in tomato? **A2-1:** We sincerely thank the reviewer for this insightful comment. We propose that additional members of the tomato SlSLAC/SLAH family may contribute to malate efflux under Al stress, yet SlSLAH2 functions as the predominant Al-induced malate transporter. Following your suggestion, we measured malate exudation in the *Slstop]* mutant after aluminum treatment and compared it with that in the *Slslah2* mutant. We found that malate exudation was significantly reduced in the *Slstop]* mutant lines under aluminum stress, indicating that SlSTOP1 alleviates aluminum toxicity in tomato through the malate exudation pathway (Figure R2A). Therefore, there must be aluminum stress-activated malate transporter downstream of the SlSTOP1 pathway. Further comparison revealed that the *Slslah2* mutant exhibited a more severe defect in malate secretion compared to the *Slstop]*mutant (Figure R2B), establishing the dominant role of SlSLAH2 in aluminum-induced malate exudation in tomato. The more severe phenotype of *Slslah2* compared to *Slstop]* indicates that even in the presence of SlSTOP1, *Slslah2* can still exhibit a severe loss of malate exudation. This genetically confirms the existence of a pathway for SlSLAH2 activation that is independent of SlSTOP1 (the SlWRKY37 pathway we identified). Furthermore, our other research findings indicate that SlSTOP1 promotes malate exudation under aluminum stress by regulating another member of the SlSLAC/SLAHs family, SlSLAH1. In that study, SlSLAH1 responds to aluminum stress and functions within the SlSTOP1 pathway (Figure R3). Moreover, SlSLAH1 likely facilitates channel opening of SlSLAH2 by forming a heteromeric complex with it (Figure R4). Our proposed mechanism is analogous to the relationship between AtSLAH1 and AtSLAH3 in Arabidopsis. Phylogenetic analysis revealed that SlSLAH1 clusters closely with AtSLAH1, while SlSLAH2 is orthologous to AtSLAH3 (Figure R5). In Arabidopsis, AtSLAH1 acts as a silent subunit that gates and activates AtSLAH3—an anion channel dependent on extracellular nitrate and kinase-mediated phosphorylation. When co-expressed, AtSLAH1 and AtSLAH3 form a heteromeric complex that enables sustained anion currents of AtSLAH3 even in the absence of nitrate or kinase activation. Under salt stress or ABA signaling, plants specifically downregulate the expression of *AtSLAH]* (but not

AtSLAH3) to reduce Cl^- transport to the shoots while preserving nitrate supply, thereby optimizing ion homeostasis to enhance salt tolerance. Thus, AtSLAH1 not only acts as a functional activator of AtSLAH3 but also serves as a molecular switch for environmental signals. Through heteromeric complex formation, it dynamically regulates AtSLAH3-mediated anion transport efficiency. Together, AtSLAH1 and AtSLAH3 constitute a key regulatory module enabling plants to adapt to salt stress. This mechanism allows fine-tuned regulation of anion homeostasis under stress conditions (Figure R6 and R7). Based on this model, we speculate that in tomato, SlSLAH2 functions downstream of SlSLAH1, and they synergistically promote malate exudation. Therefore, at least two aluminum stress-activated malate exudation pathways exist in tomato: the first is the SlSTOP1-mediated SlSLAH1 pathway, and the second is the SlWRKY37-mediated SlSLAH2 pathway. Ultimately, the aluminum stress signaling converges on SlSLAH2, establishing SlSLAH2 as the primary aluminum-induced malate transporter in tomato. We have integrated the above discussion into the "Discussion" section of the revised manuscript (line 461 to 485) to present a more comprehensive view of aluminum tolerance mechanisms in tomato.

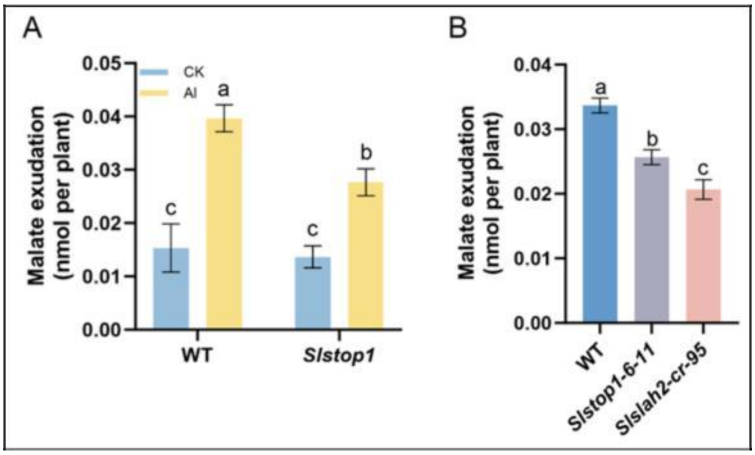

Figure R2. SlSTOP1 and SlSLAH2 operate in distinct pathways to regulate malate exudation. (A) Malate exudation in roots of WT and *Slstop]* mutant lines under aluminum stress. (B) Malate exudation in roots of WT, *Slstop]*, *Slslah2* mutant lines under aluminum stress.

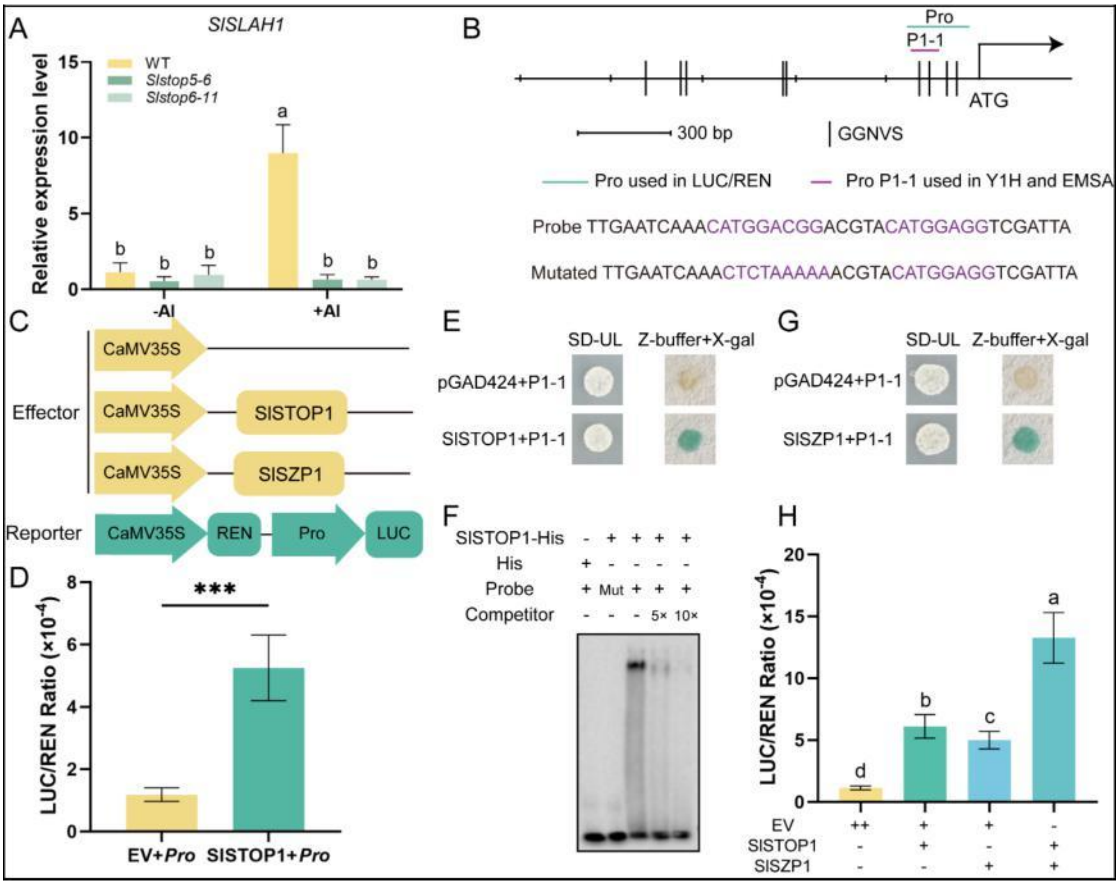

Figure R3. SlSTOP1 and SlSZP1 formed a complex activated the expression of *SlSLAH1*. (A) Expression level of *SlSLAH]* in WT and *Slstop]* knockout lines under aluminum stress. (B) Diagram of the *SlSLAH]* promoter. The GGNVS sequence of the predicted SlSTOP1-binding site was marked with vertical bar. (C) Diagram of LUC/REN assay. (D) LUC/REN assay indicated SlSTOP1 activated *SlSLAH]*. (E) Y1H assay indicated SlSTOP1 bound *SlSLAH]*. (F) EMSA assay indicated SlSTOP1 bound *SlSLAH]*. (G) Y1H assay indicated SlSZPP1 bound *SlSLAH]*. (H) LUC/REN assay indicated SlSTOP1-SlSZP1 activated *SlSLAH]*.

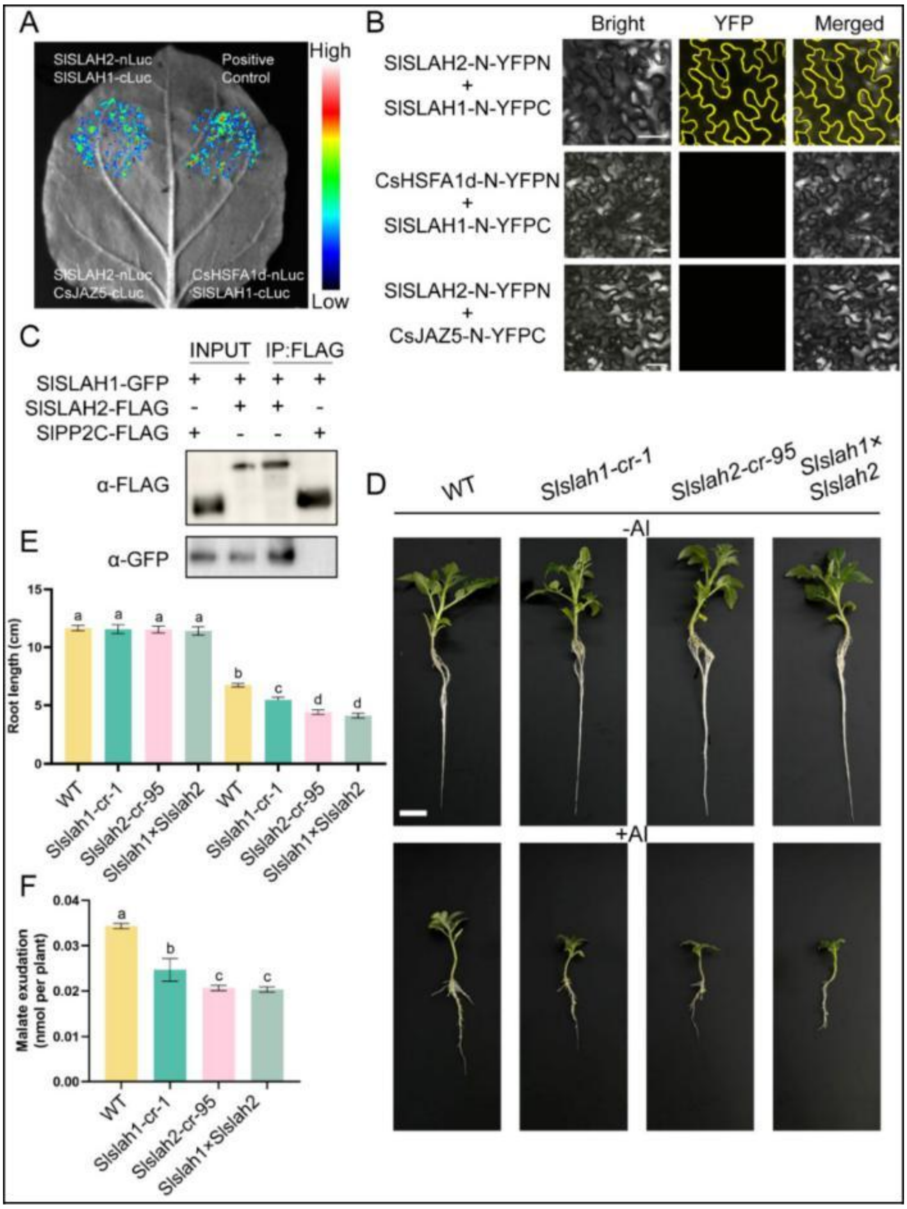

Figure R4. SlSLAH1 and SlSLAH2 formed a heteromeric complex that cooperatively regulated malate exudation under Al stress. (A) LCI assay confirming SlSLAH1-SlSLAH2 interaction. (B) Plasma membrane-localized interaction of SlSLAH1 and SlSLAH2 by BiFC. (C) Co-IP assay showed SlSLAH1 interacted with SlSLAH2 in vivo. (D) Root growth of WT, *Slslah1-cr-1*, *Slslah2-cr-95*, and *Slslah1* × *Slslah2* plants under normal condition and Al stress. (E) Root length of different lines. (F) Malate exudation of different lines after Al stress.

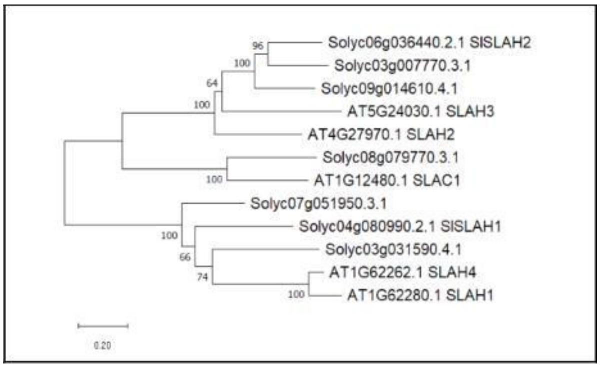

Figure R5. SlSLAH1 exhibited the closest phylogenetic relationship with AtSLAH1/4 in Arabidopsis.

Published Data Figure Redacted

Figure R6. AtSLAH1 modifies the activation properties of AtSLAH3. (A and B) Relative voltage-dependent open probabilities (rel. PO) derived from oocytes co-expressing AtSLAH3 with AtCPK21ΔEF (A) or AtSLAH3 with AtSLAH1 (B) in the presence of varying Cl^- concentrations (black) or 30 mM NO_3^-(Hedrich and Geiger, 2017).

Published Data Figure Redacted

Figure R7. AtSLAH1, together with AtSLAH3, feeds chloride into the root xylem. (A and B) Percentage of Cl^- (A) or NO_3^- (B) xylem sap concentration, relative to the wild-type (WT; 100%), in the *Atslah1-2* mutant line and in the two *Atslah1-2*/*AtSLAH1* complemented lines (P1-12 and P1-31). (C and D) Percentage of Cl^- (C) or NO_3^- (D) xylem sap concentration relative to the WT line (100%) in the *Atslah3-1* and *Atslah3-4* mutant lines (Cubero-Font et al., 2016).

Q2-2. Additionally, SlCDPK21 shows high homology with AtCPK21/23, which acts as a calcium sensor and phosphorylates STOP1. Does SlCDPK21 phosphorylate STOP1 in tomato? Please provide additional evidence and discuss in the Discussion section the relationship between SlCDPK21-mediated phosphorylation of STOP1 and its phosphorylation of SlSLAH2. How do these two pathways interact and function synergistically within the acid-aluminum stress regulatory network?

A2-2: We sincerely thank you for this constructive comment. To investigate whether SlCDPK21 performs a similar function (AtCPK21/23 phosphorylate AtSTOP1) in tomato, we first assessed the potential for direct protein-protein interaction between SlCDPK21 and SlSTOP1 by BiFC. However, no direct interaction was observed, likely due to the distinct subcellular localization of SlCDPK21 as a membrane-associated kinase (Figure S16a).

Interestingly, during our investigation, we discovered that SlCDPK20, a close homolog of SlCDPK21 in tomato, interacts with SlSTOP1 in the nucleus (as confirmed by BiFC) (Figure S16a). Moreover, in vitro kinase assays demonstrated that SlCDPK20 effectively phosphorylates SlSTOP1-MBP (Figure S16b).

Based on these results, we conclude that while SlCDPK21 and SlCDPK20 are phylogenetically related, they have evolved distinct functional roles in tomato. SlCDPK20, rather than SlCDPK21, is likely responsible for phosphorylating SlSTOP1. The aluminum stress response in tomato is orchestrated by a multi-layered SlCDPK signaling network, where distinct members assume specialized roles for a coordinated defense. This division of labor creates a two-tiered defense system. The first layer of defense is rapid and physiological: SlCDPK21 directly phosphorylates and activates the SlSLAH2 malate transporter at the plasma membrane, promoting immediate malate exudation to chelate Al^{3+} externally. Concurrently, a second, sustained layer of adaptation is achieved through

SlCDPK20, which phosphorylates the transcription factor SlSTOP1. Therefore, the stabilization of SlSTOP1 promotes its activation of downstream aluminum-responsive genes. This action initiates a transcriptional program for long-term acclimation. Thus, the SlCDPK network ensures a robust and coordinated defense, enabling tomato to combat aluminum toxicity effectively. As suggested, we have added the discussion of this part into our revised manuscript (line 527 to 547).

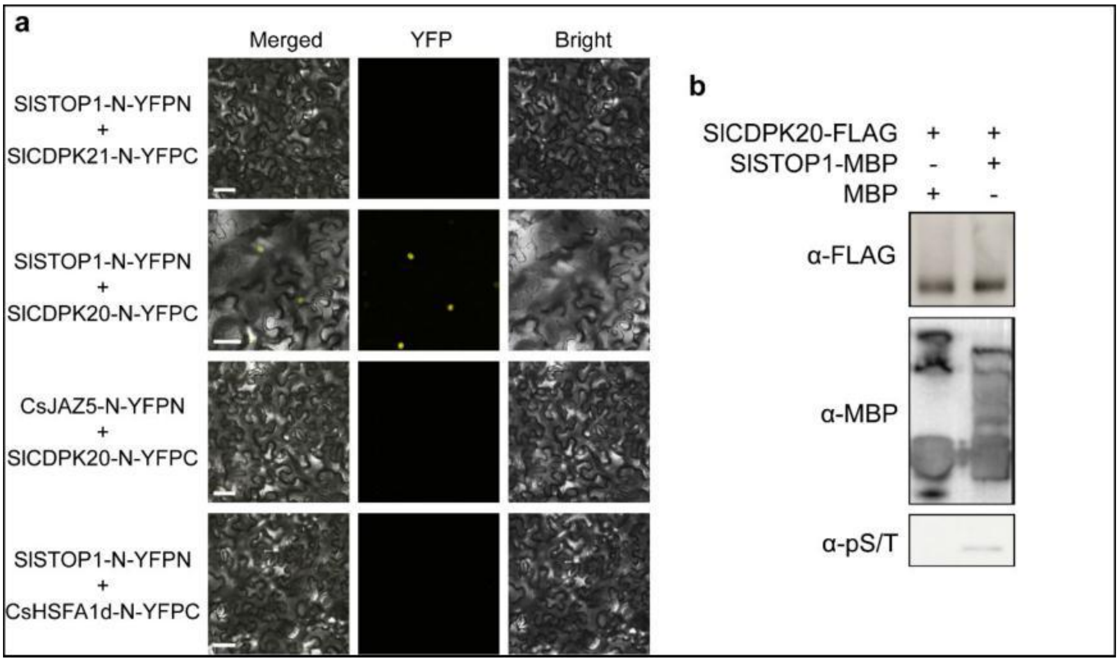

Figure S16. SlCDPK20 interacted and phosphorylated SlSTOP1. (a) BiFC assay showed the interaction between SlCDPK20 and SlSTOP1 occurred at the nuclear. (b) In vitro kinase assay showed the phosphorylation of SlSTOP1 by SlCDPK20.

Q3. In Fig. 6a, the gene expression annotation for SlPP2C67 appears to be incorrect; please verify.

A3: Thank you for your suggestion. We have checked the annotation of this PP2C in tomato genome and its homologs in other plants such as Arabidopsis and rice, we corrected its annotation as SlPP2C72.

Q4-1. Moreover, SlPP2C67 is localized to both the plasma membrane and the nucleus. Why does it exit the nucleus upon aluminum stress to be phosphorylated by SlCDPK21, thereby

inhibiting its phosphatase activity? Wouldn't direct regulation at the plasma membrane be more efficient?

A4-1: We sincerely thank you for your valuable feedback regarding the details and molecular mechanisms of SlPP2C72. We concur that the phosphorylation of SlPP2C72 by SlCDPK21 at the plasma membrane represents the most efficient regulatory mode. Our supplementary experiments have confirmed that SlPP2C72 localizes to the nucleus, cytoplasm, and plasma membrane (Figure S15c). Furthermore, BiFC assays directly demonstrated a specific interaction between SlCDPK21 and SlPP2C72 at the plasma membrane (Figure S15b). This indicates that SlPP2C72 can be directly phosphorylated by the similarly plasma membrane-localized SlCDPK21, without requiring a "nuclear export" step.

As important negative regulators of signaling, the nuclear localization of PP2C family phosphatases suggests their potential involvement in modulating nuclear signaling events, such as the activity or stability of transcription factors. Aluminum stress constitutes a complex global stress response, requiring the plant cell to coordinate responses across different compartments.

Under aluminum stress, activated SlCDPK21 preferentially phosphorylates and inhibits the activity of SlPP2C72 at the plasma membrane. This ensures the maintenance of SlSLAH2 phosphorylation status, facilitating rapid malate exudation. This constitutes the most direct and efficient regulatory mechanism, guaranteeing both the specificity and efficacy of the signaling response.

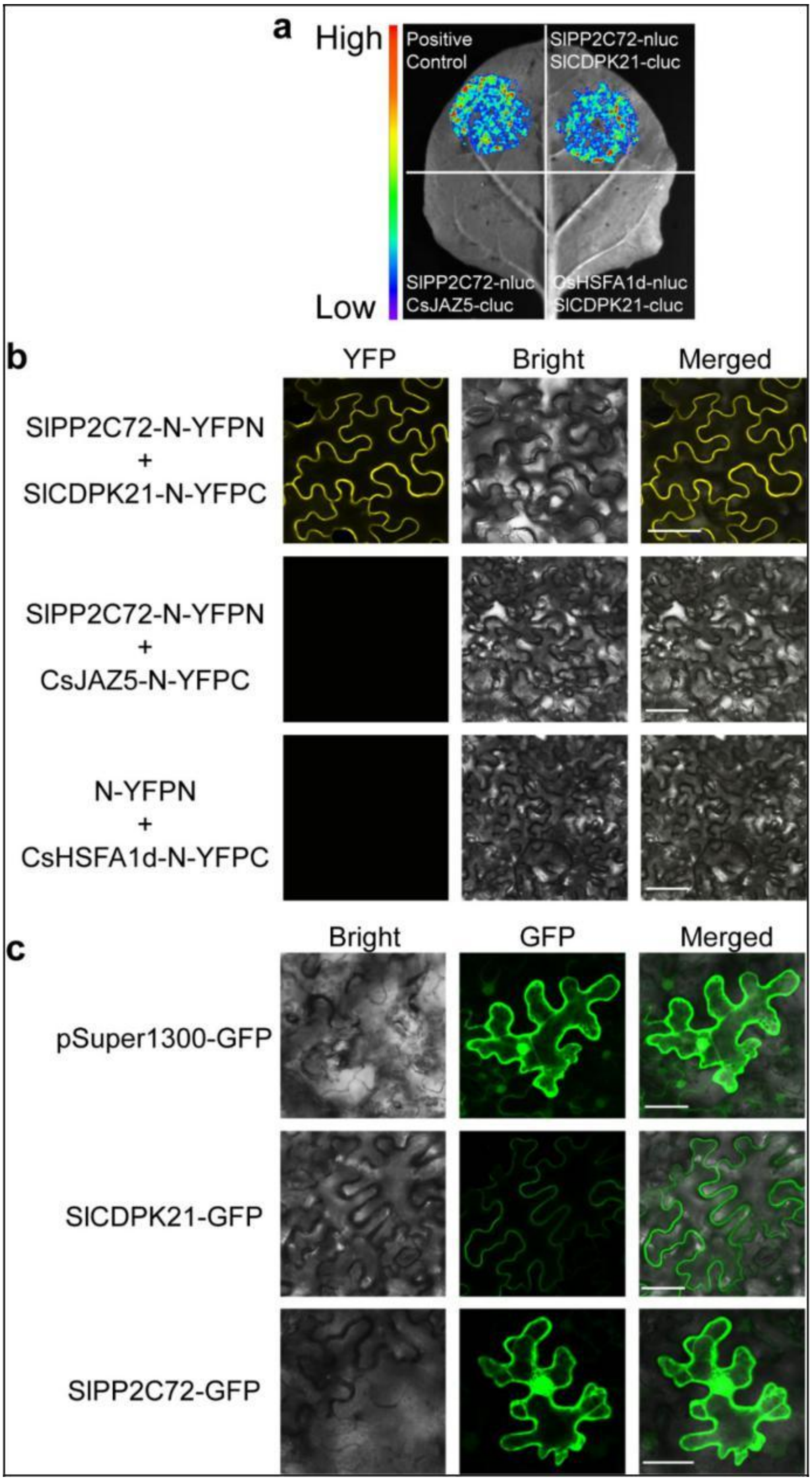

Figure S15. SlCDPK21 interacted with SlPP2C72. (a) BiFC assay showed the interaction between SlCDPK21 and SlPP2C72 occurred at the plasma membrane. (b) LCI assay showed the interaction between SlCDPK21 and SlPP2C72. (c) Subcellular location of SlPP2C72 and SlCDPK21.

Q4-2. Additional evidence is needed to demonstrate that SlCDPK21 phosphorylates SlPP2C67 to inhibit its phosphatase activity, particularly in vivo. For instance, please assess SlPP2C67 phosphatase activity in SlCDPK21 mutants.

A4-2: As recommended, we provided in vivo evidence by measuring the phosphatase activity of SlPP2C72 (Figure 8d). The *Slcdpk21* mutant group exhibited higher SlPP2C72 phosphatase activity compared to the WT group. This finding indicates that the inhibition of SlPP2C72 phosphatase activity is relieved in the absence of SlCDPK21. This result is consistent with increasing phosphorylation levels of SlPP2C72 in *Slcdpk21* mutant group (Figure 8f). We have incorporated these findings into the revised manuscript (line 355 to 356 and 364 to 367).

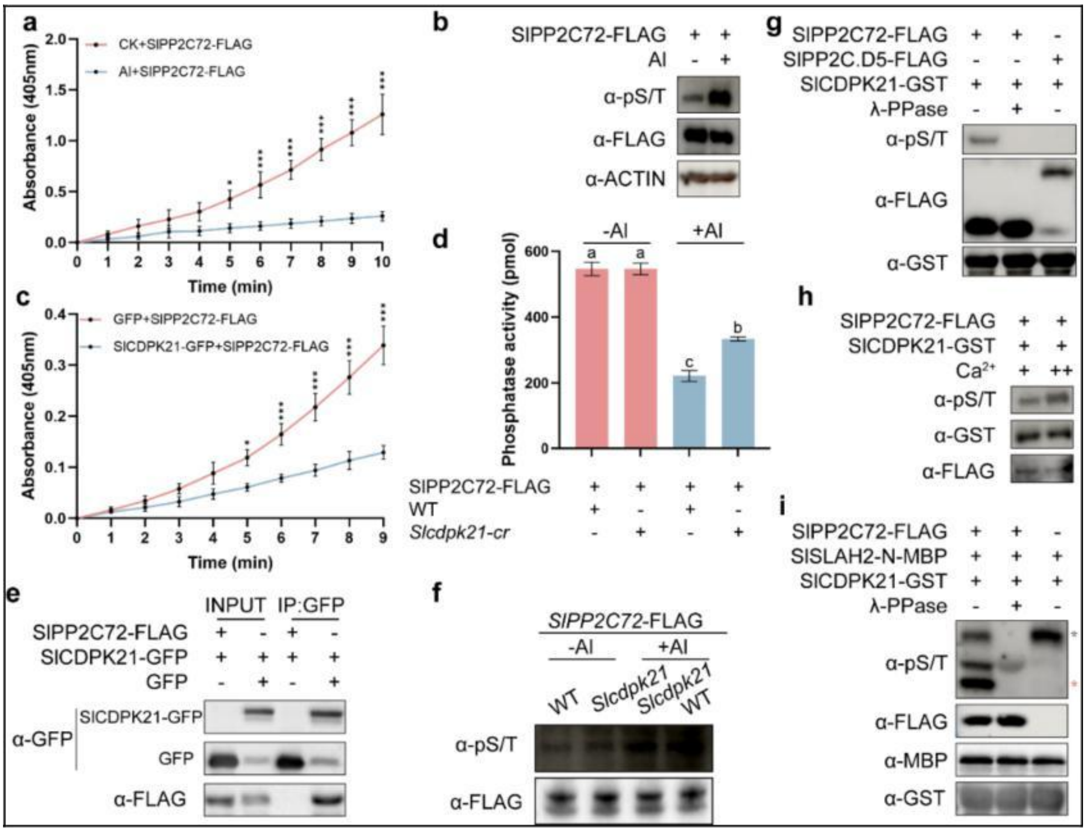

Figure 8. SlCDPK21 phosphorylated SlPP2C72 to ensure SlSLAH2 high phosphorylation state. (a) Effect of aluminum stress on phosphatase activity of SlPP2C72. (b) Phosphorylation of SlPP2C72 under aluminum stress. (c) Effect of SlCDPK21 on phosphatase activity of SlPP2C72. (d) Effect of SlCDPK21 on phosphatase activity of SlPP2C72. (e) Co-IP assay showed SlCDPK21 interacted with SlPP2C72 in vivo. (f) Phosphorylation levels of SlPP2C72 in WT and *Slcdpk21*. (g) In vitro kinase

assay showed the phosphorylation of SlPP2C72 by SlCDPK21. (h) In vitro kinase assay showed the phosphorylation of SlPP2C72 by SlCDPK21was calcium-dependent. (i) In vitro kinase assay showed SlCDPK21 simultaneously phosphorylated SlSLAH2-N terminus and SlPP2C72.

Q5-1. Additionally, include acid-aluminum stress phenotypes and malate secretion data for SlCDPK21 and SlPP2C67 mutants/overexpression lines.

A5-1: We sincerely thank you for this valuable suggestion. We completely agree with your point. This experiment was indeed part of our original plan, but at the time of the initial submission, we did not have sufficient homozygous seeds. Now, we have finally obtained enough homozygous offspring to proceed with the work. We subjected the *SlCDPK* and *SlPP2C72* mutants/overexpression lines to a long-term Al^{3+} exposure, simultaneously monitoring root elongation and malate exudation to verify whether SlCDPK21 and SlPP2C72 functioned under aluminum stress. After aluminum treatment, we observed that the *Slcdpk21* mutant and *SlPP2C72* overexpression lines exhibited significantly shorter roots under aluminum stress. Conversely, the *SlCDPK21* overexpression lines and *Slpp2c72* mutant showed significantly greater root elongation compared to the WT under aluminum stress. Furthermore, malate exudation measurements collected after short-term aluminum treatment corroborated the root growth phenotypes observed in the long-term assay. Specifically, malate secretion was significantly lower in the *Slcdpk21* mutant and *SlPP2C72* overexpression lines, while it was significantly higher in the *SlCDPK21* overexpression lines and *Slpp2c72* mutant (Figure 6e, 6f, 7e and 7f). These consistent physiological data demonstrate that SlCDPK21 positively regulates aluminum-induced malate exudation, whereas SlPP2C72 negatively regulates this process through dephosphorylation.

Q5-2. Although SlCDPK21 interacts with and inhibits SlPP2C67 phosphatase activity, SlPP2C67 does not directly interact with SlSLAH2 yet can dephosphorylate it. What is the mechanistic basis for this? Please discuss and provide in vivo evidence—for example, the phosphorylation status of SlSLAH2 in SlPP2C67 mutants.

A5-2: We sincerely thank you for raising this key mechanistic question. You have correctly identified a pivotal link in our model: how SlPP2C72 dephosphorylates SlSLAH2 without

directly interacting with it. This is indeed central to elucidating the completeness of this regulatory circuit. We fully agree with your perspective and, following your suggestion, we have obtained critical in vivo evidence to support this model by analyzing the phosphorylation status of SlSLAH2 co-incubated with *Slpp2c72* and WT. Under aluminum stress conditions, the phosphorylation level of SlSLAH2 in the *Slpp2c72* group was significantly elevated compared to WT (Figure 7f). Therefore, this confirms that SlPP2C72 is the key phosphatase responsible for the in vivo dephosphorylation of SlSLAH2.

Based on the phenomenon that "SlPP2C72 dephosphorylates SlSLAH2 without direct binding," we hypothesize that the mechanistic basis involves the presence of one or more unknown intermediary factors. We speculate that such an intermediary factor could function through the following possibilities: (1) A scaffold protein might exist that can simultaneously bind to both SlPP2C72 and SlSLAH2. This would recruit the phosphatase into close proximity with its substrate, enabling specific dephosphorylation. (2) SlPP2C72 might dephosphorylate a specific protein kinase that acts upstream of SlSLAH2, indirectly leading to a reduction in the phosphorylation level received by SlSLAH2 – effectively creating a dephosphorylation. A similar phenomenon has been reported. For instance, in maize, ZmSLAC1 is activated by phosphorylation through the ZmSIMK1-ZmMEK1 cascade. ZmPP84, an F-clade PP2C, dephosphorylates ZmMEK1, thereby attenuating the phosphorylation level of ZmSLAC1 (Guo et al., 2023). Therefore, it is highly plausible that a protein kinase activating SlSLAH2 also exists in tomato, and the phosphorylation status of this kinase is regulated by SlPP2C72. Consequently, SlPP2C72 indirectly regulates the phosphorylation level of SlSLAH2. As suggested, we have added this part in our discussion part in revised manuscript (line 559 to 574).

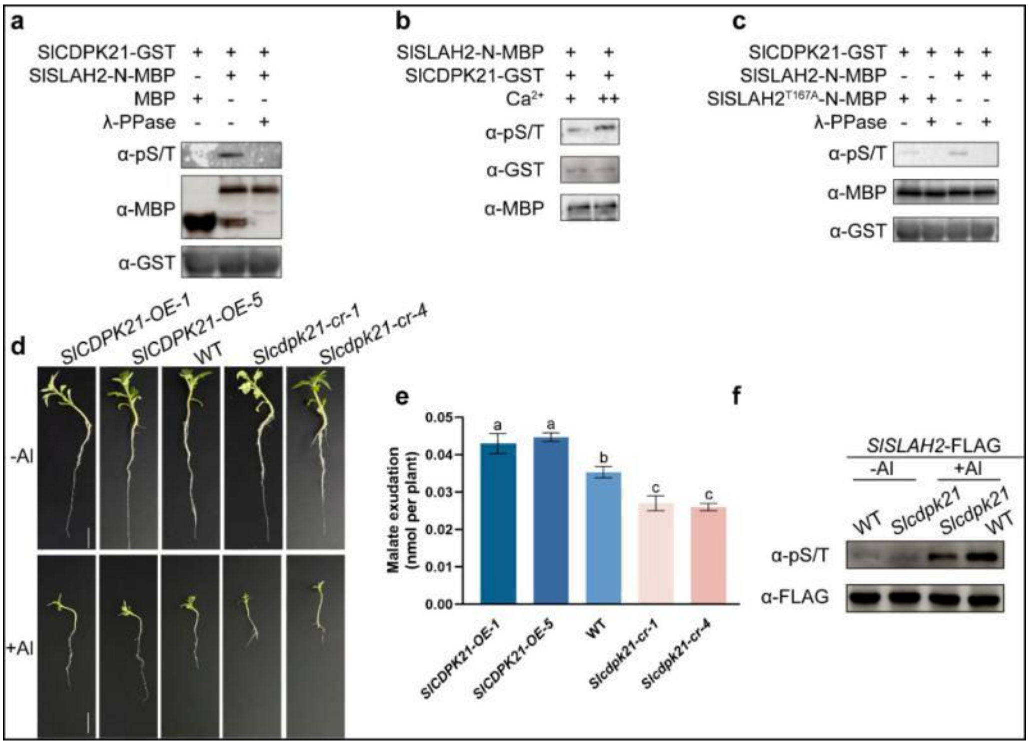

Figure 6. SlCDPK21 enhanced aluminum tolerance in tomato by increasing phosphorylation of SlSLAH2. (a) In vitro kinase assay showed the phosphorylation of SlSLAH2 -N by SlCDPK21. (b) In vitro kinase assay showed the phosphorylation of SlSLAH2 by SlCDPK21 was calcium-dependent. (c) In vitro kinase assay showed T167 of SlSLAH2 was a target of SlCDPK21. (d) Root growth of WT, *SlCDPK2]* overexpression lines and knockout lines under normal condition and aluminum stress. (e) Malate exudation of different lines. (f) Phosphorylation levels of SlSLAH2 in WT and *Slcdpk2]*.

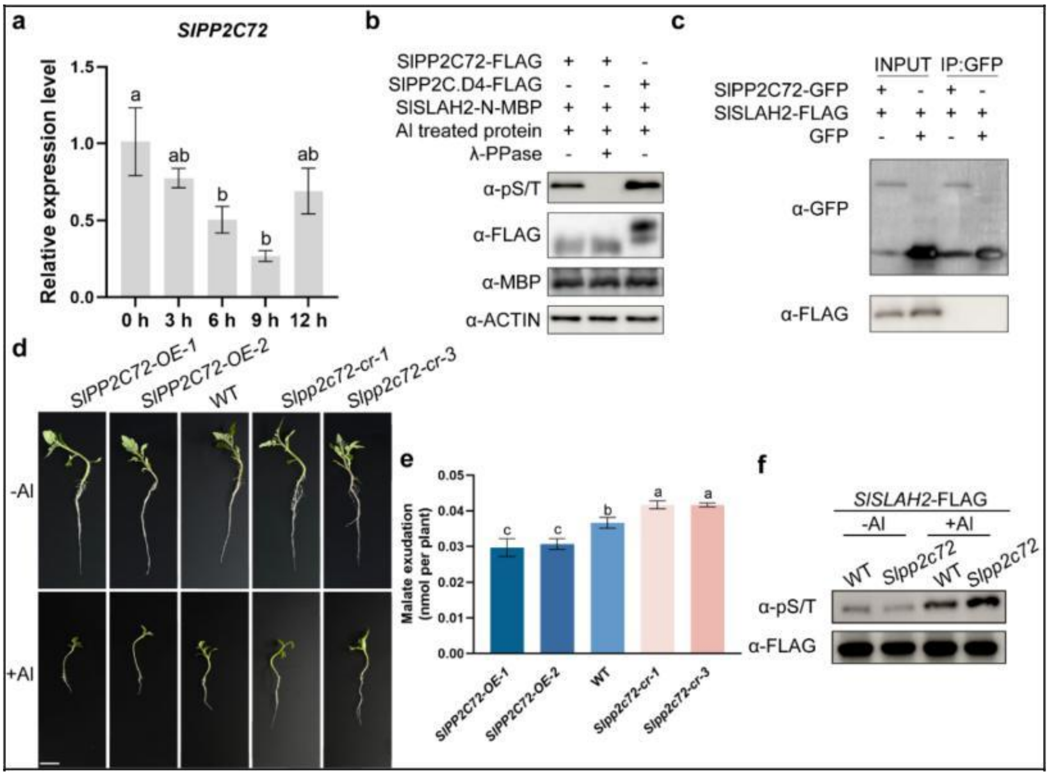

Figure 7. SlSLAH2 was indirectly dephosphorylated by SlPP2C72. (a) The expression level of *SlPP2C72* in WT under aluminum stress. (b) SlSLAH2 was dephosphorylated by SlPP2C72 in vitro. (c) Co-IP assay showed SlSLAH2 did not interact with SlPP2C72 in vivo. (d) Root growth of WT, *SlPP2C72* overexpression lines and knockout lines under normal condition and aluminum stress. (f) Phosphorylation levels of SlSLAH2 in WT and *Slpp2c72*.

Q6. Please provide data on malate secretion levels in wrky37 mutants.

A6: We sincerely thank you for this crucial suggestion. As recommended, we have supplemented the malate secretion data for the *Slwrky37* mutant (Figure 4c). The experimental results demonstrate that, compared to the WT, the *Slwrky37* mutant exhibits a significant reduction in malate secretion under aluminum stress. This phenotype aligns with that observed in the *Slslah2* mutant, providing key physiological functional evidence for the central role of the "SlWRKY37-SlSLAH2" transcriptional regulatory module in aluminum-induced malate exudation. This data has been incorporated into the revised manuscript (Figure 4c).

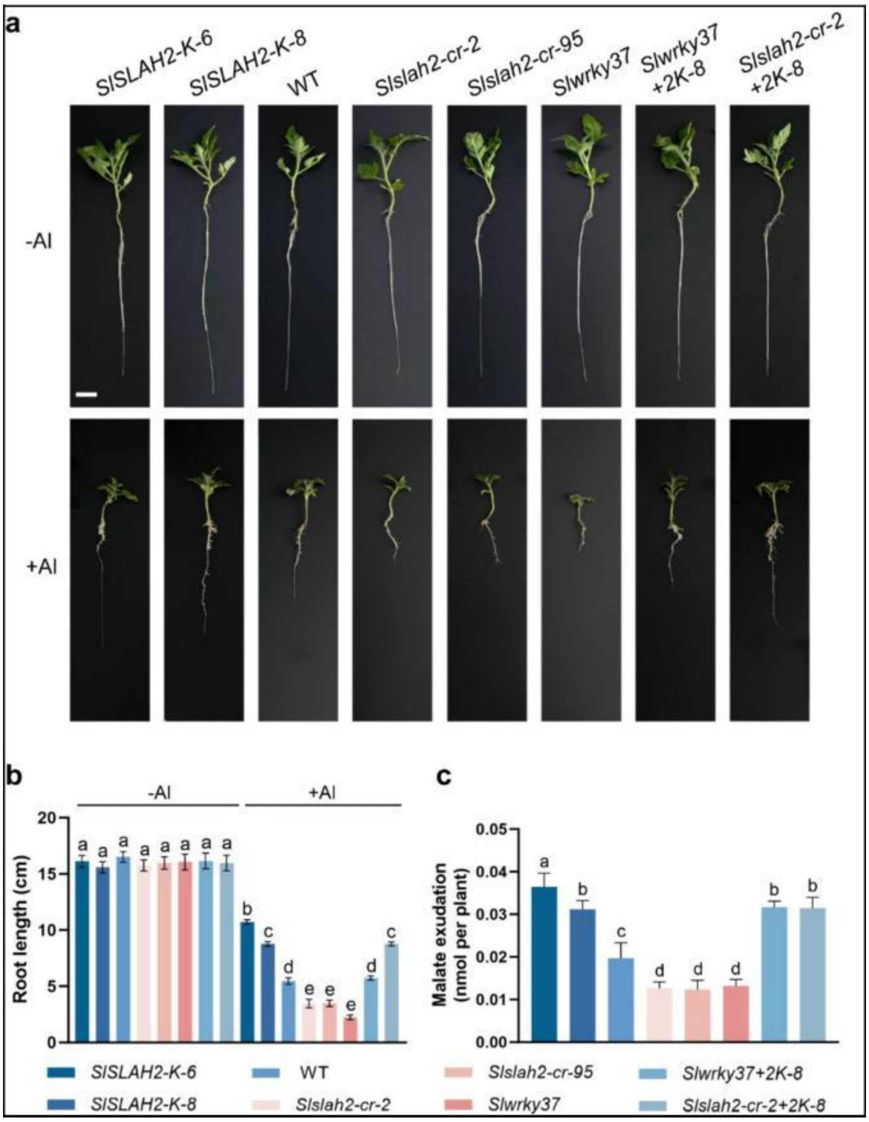

Figure 4. SlSLAH2 enhanced the aluminum tolerance by increasing the exudation of malate in tomato. (a) Root growth of WT, *SlSLAH2* overexpression lines and knockout lines under normal condition and aluminum stress. (b) Root length of different lines. (c) Malate exudation of different line under aluminum stress.

Minor Concerns:

Q1. Aluminum-induced organic acid secretion occurs primarily in the root tip. Please supplement with spatial expression patterns of each gene involved.

A1: We sincerely thank you for your valuable suggestion. As recommended, we have conducted an analysis of the expression patterns of *SlSLAH2*, *SlWRKY37*, *SlCDPK21*, and *SlPP2C72* in the root tips. The results clearly demonstrate that all four genes are expressed in

the root tip region, which is the primary site for aluminum perception and response. We have added these results in Figure S8, S9 and S12.

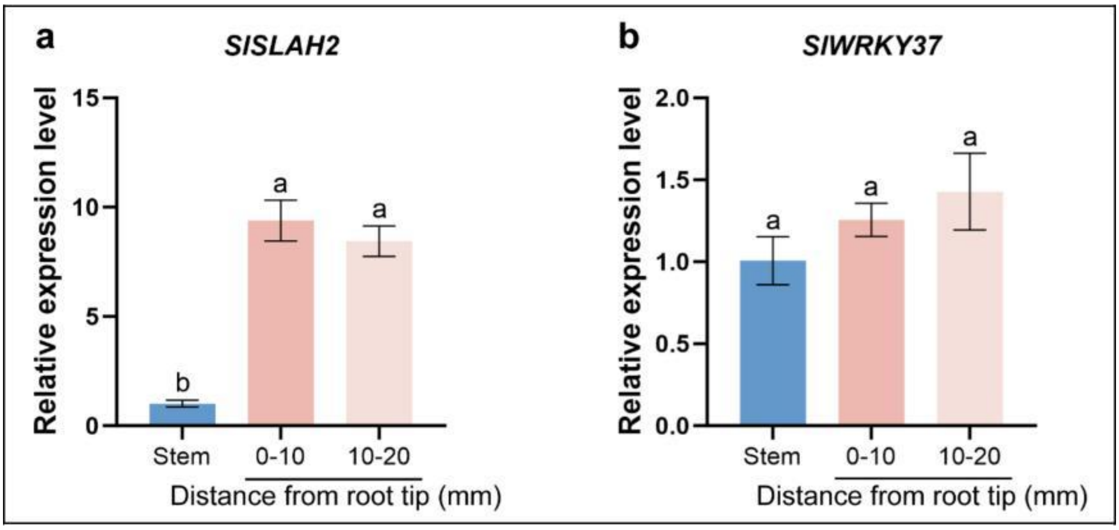

Figure S8. *SlSLAH2* and *SlWRKY37* were expressed at root tip. Relative expression levels of *SlSLAH2* (a) and *SlWRKY37* (b) in root tips and stem.

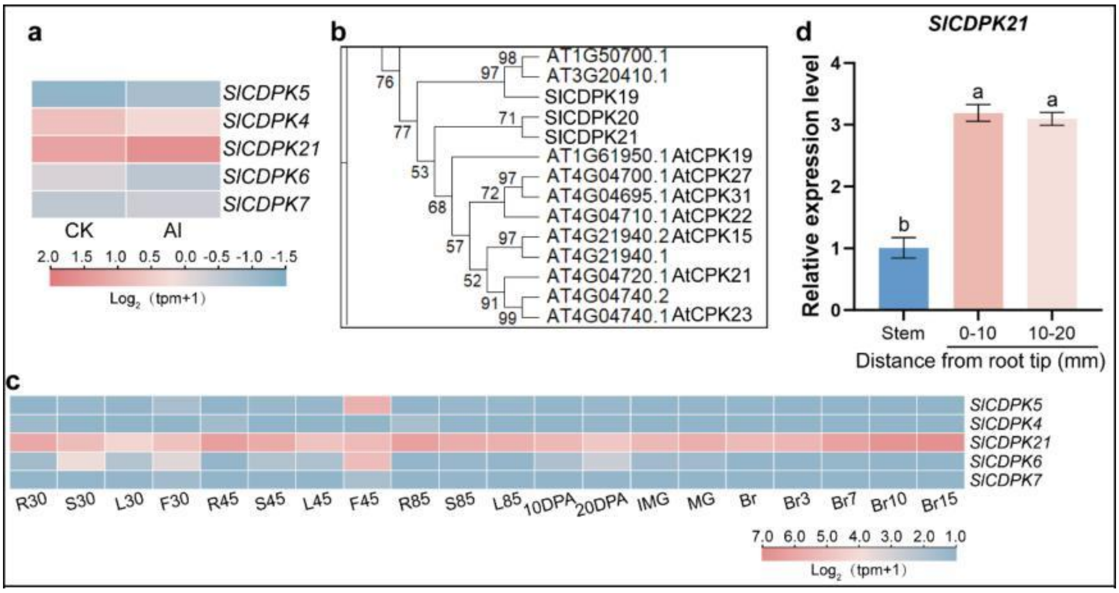

Figure S9. *SlCDPK21* responded to aluminum stress and was expressed at roots. (a) Heatmap analysis of *SlCDPK* genes in WT under aluminum stress. (b) Part phylogenetic tree of SlCDPK in tomato and AtCDPK in Arabidopsis. (c) Tissue expression of *SlCDPKs*. (d) Relative expression level of *SlCDPK2]* in root tips and stem.

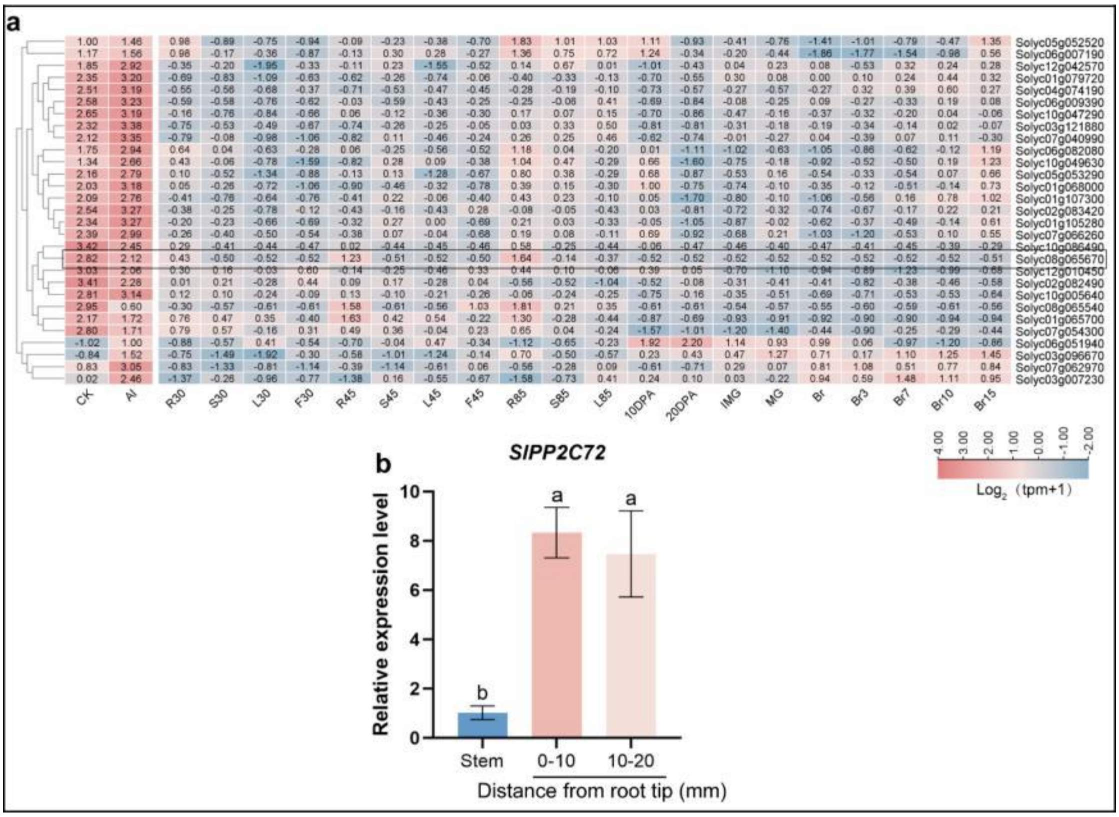

Figure S12. *SlPP2C72* was inhibited by aluminum stress and expressed at root tip. (a) Heatmap analysis of *SlPP2C* genes (left) in WT under aluminum stress, (right) in different tissue. (b) Relative expression level of *SlPP2C72* in root tips and stem.

Q2. Please specify the age of seedlings used in the experiments.

A2: We sincerely thank you for this valuable suggestion. As recommended, we have now explicitly specified the seedling age used in all experiments throughout the manuscript.

Reviewer #2:

The exudation of organic acids, such as malate and citrate, mediated by ALMTs and MATEs, is regarded as a major mechanism of aluminum (Al) resistance in plants. Studies have found that plants may utilize either citrate or malate, or both to chelate external Al. In this manuscript, Dong et al. identified SlSLAH2 is associated with aluminum toxicity in tomato. The authors intend to uncover the molecular mechanisms underlying how Al regulates SlSLAH2 activity, thereby conferring Al resistance. The manuscript provides a substantial amount of data; however, I still have several questions.

Q1-1. Figure 1: It is curious to note that nearly all of the genes from the ALMT family are reduced by Al (Figure 1). Although the authors discussed the possible role of SlALMT9 in Al toxicity as located in the vacuolar membrane, its overexpression significantly enhances malate exudation by Al (Ye et al., 2017).

A1-1: We sincerely thank you for this valuable suggestion. We fully acknowledge the findings reported by Ye et al. and contend that their results do not contradict our own, but rather reveal a multi-layered, stage-specific, and finely regulated strategy employed by tomato in response to aluminum stress. As demonstrated by Ye et al., the *SlALMT9* promoter is activated under high Al^{3+} concentrations (200 μM) (Figure R1). Given its tonoplast localization, SlALMT9 does not function in exuding malate to rhizosphere but instead regulates malate flux between the vacuole and the cytoplasm [a bidirectional transport capability also observed for OsALMT7 in rice (Heng et al., 2018)]. Its Arabidopsis homolog, AtALMT9, has been confirmed to maintain vacuolar malate homeostasis. Concurrently, overexpression of *SlALMT9* driven by the *CaMV35S* promoter significantly enhanced malate exudation under aluminum stress. We propose that the induction of *SlALMT9* under severe aluminum stress represents a "survival strategy": plants may actively mobilize malate stored in vacuoles, particularly from sink organs such as fruits. The released malate can serve two critical functions: 1) chelating Al^{3+} that has entered the cytoplasm to alleviate intracellular toxicity, and 2) being transported to the roots via long-distance transport or other mechanisms to provide substrate support for the sustained, high-level malate exudation mediated by

SlSLAH2 at the rhizosphere. Integrating evidence from our work and that of Ye's team, we propose the following model to explain the potential relationship between SlALMT9 and SlSLAH2. **Mild/Moderate Aluminum Stress**: When plant roots perceive Al^{3+} in the soil, they rapidly activate an external detoxification mechanism primarily mediated by SlSLAH2. SlSLAH2 is transcriptionally activated by SlWRKY37 and post-translationally modified via phosphorylation by SlCDPK21, enabling efficient malate secretion into the rhizosphere for direct Al^{3+} chelation. During this phase, to prioritize resource allocation to this immediate defense response, the plant may downregulate genes involved in other non-essential metabolic pathways, such as those related to fruit acid maintenance involving SlALMT9. **Severe Aluminum Stress/Later Stage**: As aluminum stress intensifies, some Al^{3+}may break the plant's external defenses, enter the plant body, and potentially translocate to above-ground part of the plant. At this stage, the plant activates an internal tolerance mechanism predominately governed by SlALMT9. We hypothesize that the induced expression of *SlALMT9* under severe aluminum stress represents a "survival strategy": the plant may actively mobilize malate stored in vacuoles, particularly in sink organs like fruits. The released malate could be used in the cytoplasm to chelate Al^{3+} that have entered the cell, mitigating intracellular toxicity. Alternatively, it might be transported over long distances or via other means to the roots, providing substrate support for the sustained, high-intensity malate exudation in the rhizosphere. We have integrated the above discussion into the "Discussion" section of the revised manuscript (line 439 to 460) to present a more comprehensive view of the complexity of aluminum tolerance mechanisms in tomato.

Published Data Figure Redacted

Figure R1. Histochemical localization of GUS activity in roots of *SlALMT9*^{HMH} pro::GUS. (Ye et al., 2017).

Q1-2. Additionally, SlSTOP1 plays a major role in Al-induced organic acid exudation; but it cannot bind with the promoter of SlSLAH2. Therefore, SlSLAH2 may not play a predominant role in Al-induced malate exudation. The authors should interpret in the discussion section.

A1-2: Actually, we have investigated the SlSTOP1 mediated *SlSLAH* pathway. Results showed that SlSTOP1 promotes aluminum stress-induced malate exudation by regulating *SlSLAH1*, another member of the SlSLAC/SLAHs family. In this study, SlSLAH1 responds to aluminum stress and functions within the SlSTOP1 pathway (Figure R2 and R3). Furthermore, SlSLAH1 likely facilitates channel opening by forming heteromeric complexes with SlSLAH2 (Figure R4). The mechanism we propose is analogous to the relationship between AtSLAH1 and AtSLAH3 in Arabidopsis. Phylogenetic analysis reveals that SlSLAH1 clusters closely with AtSLAH1, while SlSLAH2 is homologous to AtSLAH3 (Figure R5). In Arabidopsis, AtSLAH1 acts as a silent subunit that gates and activates the anion channel AtSLAH3 in a manner dependent on extracellular nitrate and kinase-mediated phosphorylation (Figure R6 and R7) (Cubero-Font et al., 2016).

Based on this model, we speculate that in tomato, SlSLAH2 functions downstream of SlSLAH1, and they act synergistically to promote malate exudation. Therefore, at least two aluminum stress-activated malate exudation pathways exist in tomato: the first is the SlSTOP1-mediated SlSLAH1 pathway, and the second is the SlWRKY37-mediated SlSLAH2

pathway. Ultimately, the aluminum stress signaling converges on SlSLAH2, establishing SlSLAH2 as the primary aluminum-induced malate transporter in tomato. As suggested, we have added these into the discussion part (line 461 to 485).

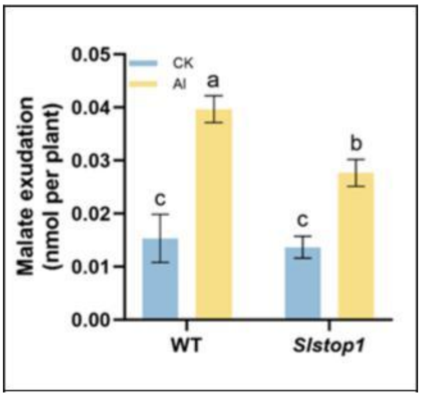

Figure R2. SlSTOP1 regulates malate exudation to enhance tomato aluminum tolerance. Malate exudation was measured in roots of WT and *Slstop]* mutant lines under aluminum stress.

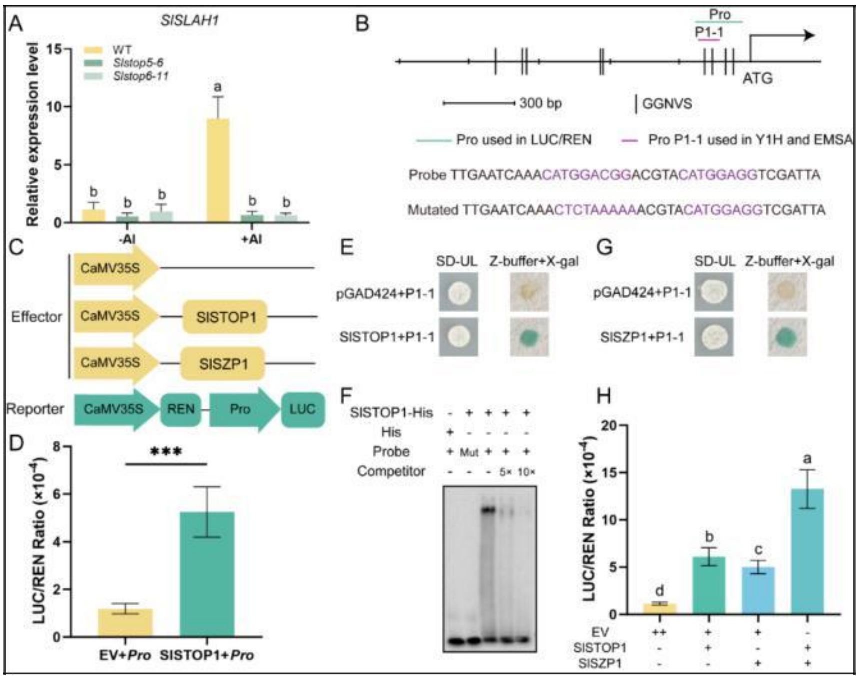

Figure R3. SlSTOP1 and SlSZP1 formed a complex activated the expression of *SlSLAH1*. (A) Expression level of *SlSLAH]* in WT and *Slstop]* knockout lines under aluminum stress. (B) Diagram of the *SlSLAH]* promoter. The GGNVS sequence of the predicted SlSTOP1-binding site was marked with vertical bar. (C) Diagram of LUC/REN assay. (D) LUC/REN assay indicated SlSTOP1 activated *SlSLAH]*. (E) Y1H assay indicated SlSTOP1 bound *SlSLAH]*. (F) EMSA assay indicated SlSTOP1

bound *SlSLAH1*. (G) Y1H assay indicated SlSZPP1 bound *SlSLAH1*. (H) LUC/REN assay indicated SlSTOP1-SlSZP1 activated *SlSLAH1*.

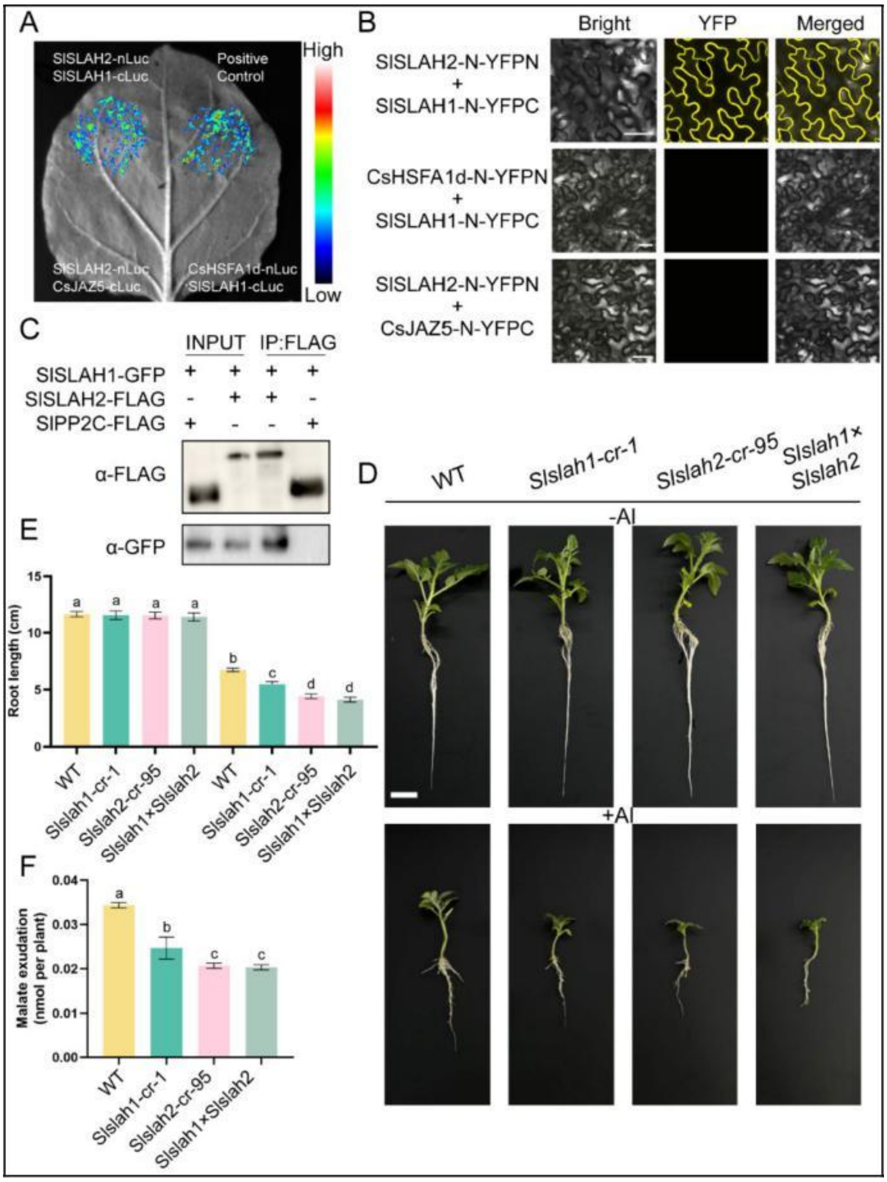

Figure R4. SlSLAH1 and SlSLAH2 formed a heteromeric complex that cooperatively regulated malate exudation under Al stress. (A) LCI assay confirming SlSLAH1-SlSLAH2 interaction. (B) Plasma membrane-localized interaction of SlSLAH1 and SlSLAH2 by BiFC. (C) Co-IP assay showed SlSLAH1 interacted with SlSLAH2 in vivo. (D) Root growth of WT, *Slslah1-cr-1*, *Slslah2-cr-95*, and *Slslah1* × *Slslah2* plants under normal condition and Al stress. (E) Root length of different lines. (F) Malate exudation of different lines after Al stress.

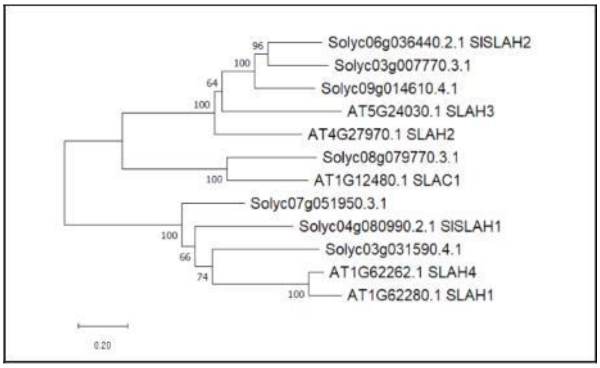

Figure R5. SlSLAH1 exhibited the closest phylogenetic relationship with AtSLAH1/4 in Arabidopsis.

Published Data Figure Redacted

Figure R6. AtSLAH1 modifies the activation properties of AtSLAH3. (A and B) Relative voltage-dependent open probabilities (rel. PO) derived from oocytes co-expressing AtSLAH3 with AtCPK21ΔEF (A) or AtSLAH3 with AtSLAH1 (B) in the presence of varying Cl^- concentrations (black) or 30 mM NO_3^- (Cubero-Font et al., 2016).

Published Data Figure Redacted

Figure R7. AtSLAH1, together with AtSLAH3, feeds chloride into the root xylem. (A and B) Percentage of Cl^- (A) or NO_3^- (B) xylem sap concentration, relative to the wild-type (WT; 100%), in the *Atslah]-2* mutant line and in the two *Atslah]-2/AtSLAH]* complemented lines (P1-12 and P1-31). (C and D) Percentage of Cl^- (C) or NO_3^- (D) xylem sap concentration relative to the WT line (100%) in the *Atslah3-]* and *Atslah3-4* mutant lines (Cubero-Font et al., 2016).

Q2-1. Figure 2: Data from E. coli to confirm the role of SlSLAH2 in malate transport (possible influx or adsorption) is insufficient. I would suggest that the authors use Xenopus oocytes to determine whether this protein mediates malate efflux. Furthermore, root elongation and Al content should also be included here.

Q2-2. Figure 4b: Root length and malate concentrations should be presented as two separate figures.

A2-1: Thank you very much for your valuable suggestions. As suggested, we have added patch-clamp experiments to demonstrate that SlSLAH2, as an anion transport channel, can transport not only malate but also nitrate and chloride (Figure 2b and S3). By expressing SlSLAH2 in *Xenopus* oocytes and applying 50 mM solutions containing the corresponding anions, we found that all these anions can activate anion currents of SlSLAH2, with a greater anion current response observed for nitrate.

As recommended, we have now added comprehensive root elongation data and Al content measurements into the revised manuscript, specifically in Figures 2e, 2f, 4b, and 4c. Aluminum stress treatment significantly inhibits both root elongation and malate exudation in the *Slslah2* mutant (Figure 2e, 4b and 4c). Furthermore, hematoxylin staining of roots subjected to short-term aluminum treatment revealed substantially greater Al accumulation in the *Slslah2* mutant compared to the WT (Figure 2f). This finding provides direct visual evidence that the loss of *SlSLAH2* function compromises the plant's ability to exclude aluminum from root tissues.

A2-2: As suggested, we have now presented the root length and malate concentrations separately in the revised manuscript (Figure 4b and 4c).

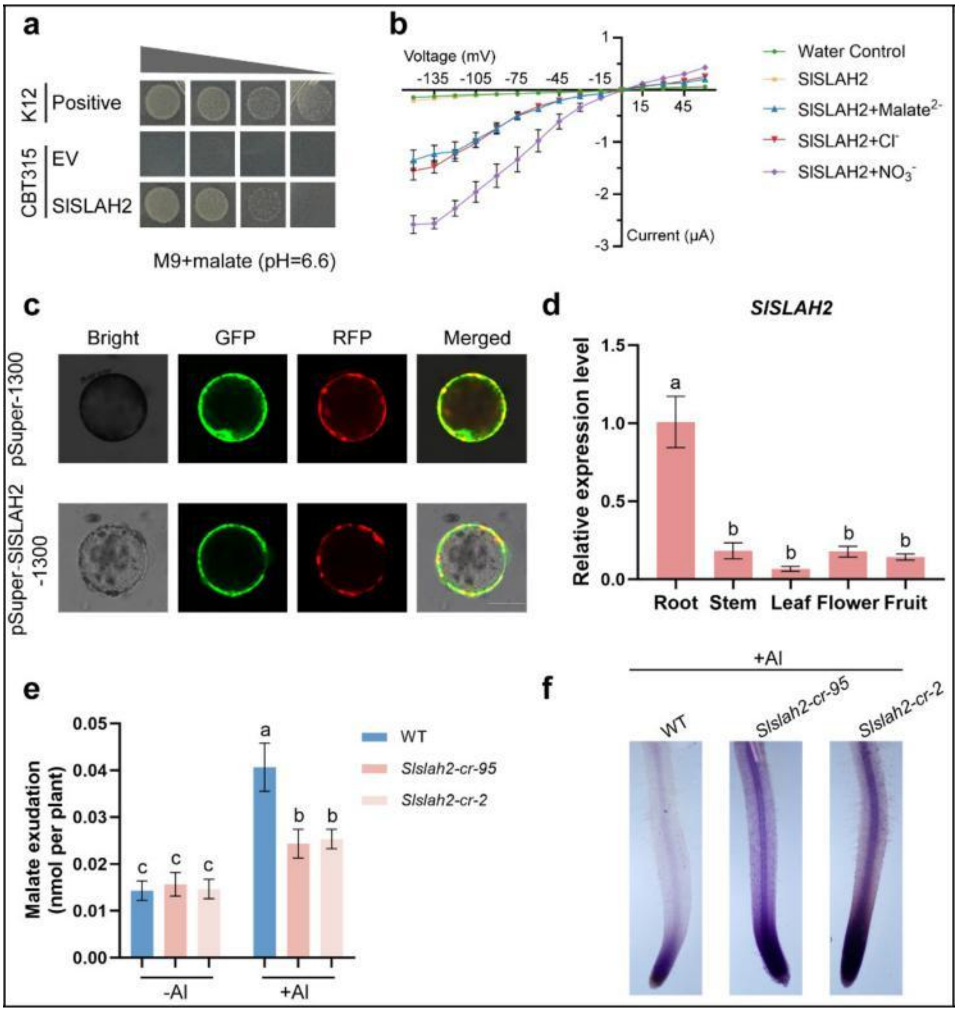

Figure 2. SlSLAH2 was a plasma membrane-localized malate transporter. (a) SlSLAH2 partly restored the growth defect of dicarboxylate-uptake-deficient *E. coli* mutant CBT315. **(b)** SlSLAH2 was an anion channel. **(c)** Subcellular location of SlSLAH2. **(d)** Tissue-specific expression analysis of *SlSLAH2* in tomato. **(e)** Malate exudation in roots of MicroTom WT lines and *Slslah2* mutant lines, under aluminum stress. **(f)** Hematoxylin staining of root Al content.

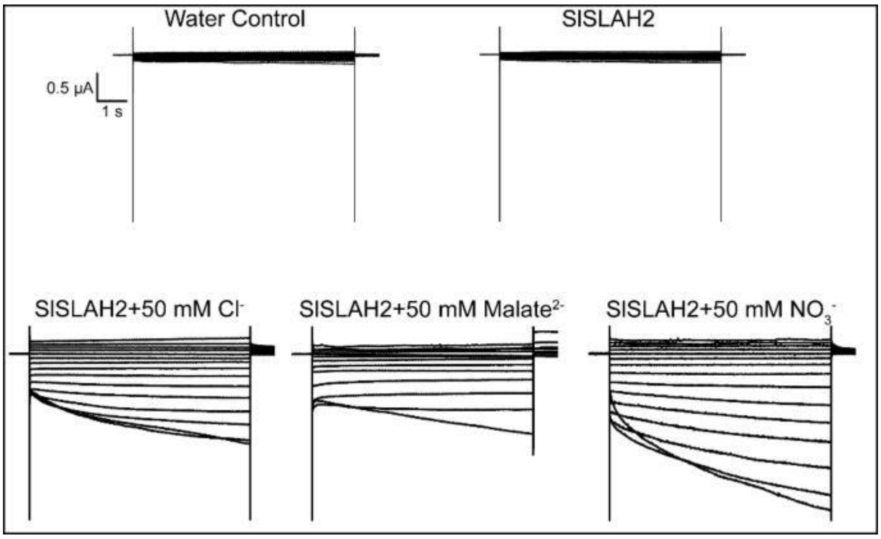

Figure S3. **SlSLAH2 was an anion transporter.** Typical whole-cell current traces.

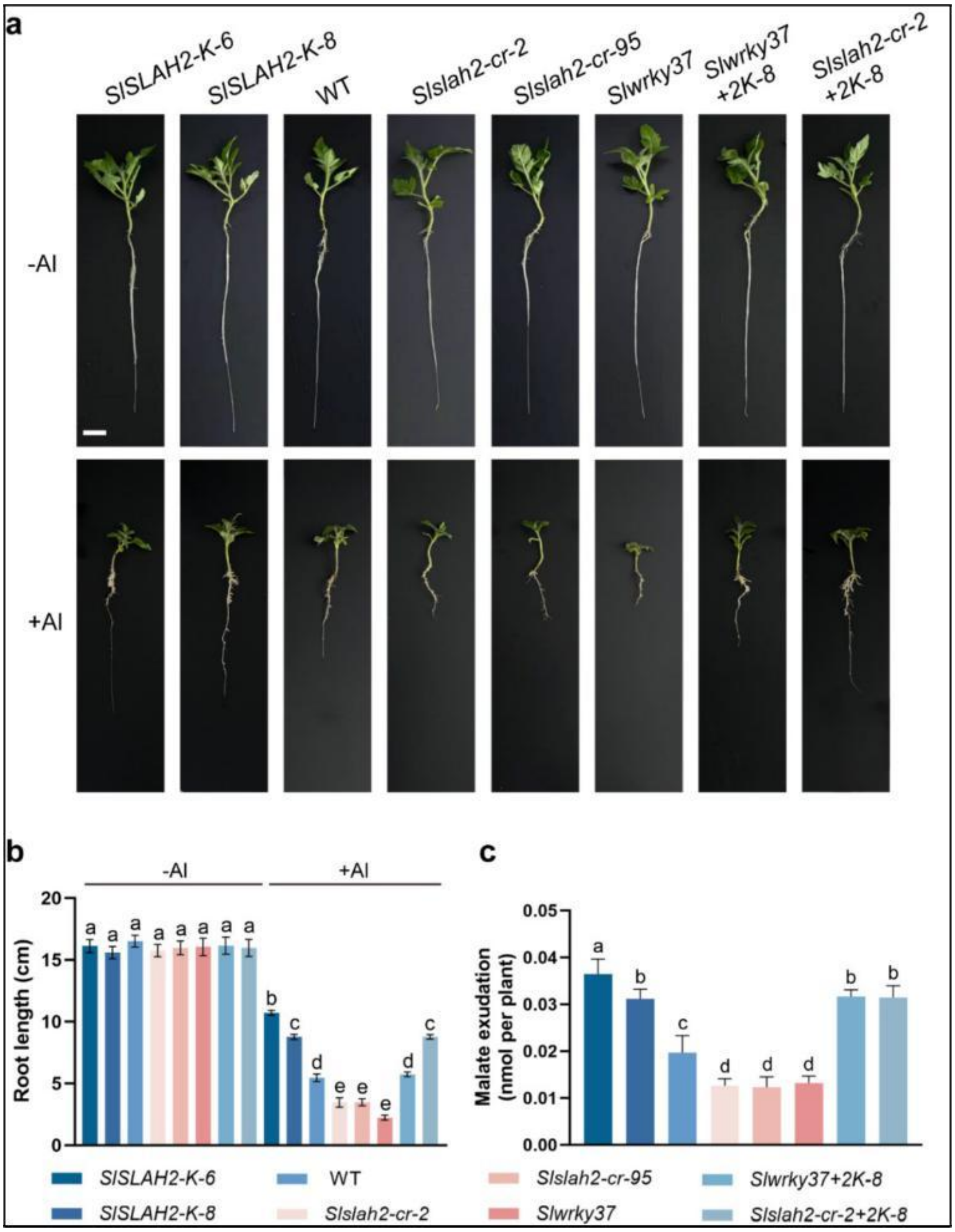

Figure 4. SlSLAH2 enhanced the aluminum tolerance by increasing the exudation of malate in tomato. (a) Root growth of WT, *SlSLAH2* overexpression lines and knockout lines under normal condition and aluminum stress. **(b)** Root length of different lines. **(c)** Malate exudation of different lines.

Q3-1. Figure 6: Protein phosphorylation and dephosphorylation are dependent on the interactions of kinases and phosphatases with their targets. How SlPP2C67 could dephosphorylate SlCDPK21 without direct interaction?

Q3-2. Considering the significant roles of SlCDPK21 and SlPP2C67 in regulating SlSLAH2 activity, I recommend that the authors further investigate their involvement in Al-induced malate exudation and Al resistance by utilizing mutant and overexpressing transgenic plants.

A3-1: We sincerely thank you for raising this key mechanistic question. You have correctly identified a pivotal link in our model: how SlPP2C72 (following Reviewer 1's suggestion, we have corrected the annotation of SlPP2C67 to SlPP2C72) dephosphorylates SlSLAH2 without directly interacting with it. This is indeed central to elucidating the completeness of this regulatory circuit.

Based on the phenomenon that "SlPP2C72 dephosphorylates SlSLAH2 without direct binding," we hypothesize that the mechanistic basis involves the presence of one or more unknown intermediary factors. We speculate that such an intermediary factor could function through the following possibilities: (1) A scaffold protein might exist that can simultaneously bind to both SlPP2C72 and SlSLAH2. This would recruit the phosphatase into close proximity with its substrate, enabling specific dephosphorylation. (2) SlPP2C72 might dephosphorylate a specific protein kinase that acts upstream of SlSLAH2, indirectly leading to a reduction in the phosphorylation level received by SlSLAH2 – effectively creating a dephosphorylation. A similar phenomenon has been reported. For instance, in maize, ZmSLAC1 is activated by phosphorylation through the ZmSIMK1-ZmMEK1 cascade. ZmPP84, an F-clade PP2C, dephosphorylates ZmMEK1, thereby attenuating the phosphorylation level of ZmSLAC1 (Guo et al., 2023). Therefore, it is highly plausible that a protein kinase activating SlSLAH2 also exists in tomato, and the phosphorylation status of this kinase is regulated by SlPP2C72. Consequently, SlPP2C72 indirectly regulates the phosphorylation level of SlSLAH2. We have added this part in discussion part (line 559 to 574).

A3-2: We sincerely thank you for this valuable suggestion. We completely agree with your point. This experiment was indeed part of our original plan, but at the time of the initial

submission, we did not have sufficient homozygous seeds. Now, we have finally obtained enough homozygous offspring to proceed with the work. We subjected the *SlCDPK* and *SlPP2C72* mutants/overexpression lines to a long-term Al^{3+} exposure, simultaneously monitoring root elongation and malate exudation to verify whether SlCDPK21 and SlPP2C72 functioned under aluminum stress. After aluminum treatment, we observed that the *Slcdpk21* mutant and *SlPP2C72* overexpression lines exhibited significantly shorter roots under aluminum stress. Conversely, the *SlCDPK21* overexpression lines and *Slpp2c72* mutant showed significantly greater root elongation compared to the WT under aluminum stress. Furthermore, malate exudation measurements collected after short-term aluminum treatment corroborated the root growth phenotypes observed in the long-term assay. Specifically, malate secretion was significantly lower in the *Slcdpk21* mutant and *SlPP2C72* overexpression lines, while it was significantly higher in the *SlCDPK21* overexpression lines and *Slpp2c72* mutant (Figure 6e, 6f, 7e and 7f). These consistent physiological data demonstrate that SlCDPK21 positively regulates aluminum-induced malate exudation, whereas SlPP2C72 negatively regulates this process through dephosphorylation.

We have obtained critical in vivo evidence to support SlCDPK21 phosphorylated SlSLAH2 and SlPP2C72 dephosphorylated SlSLAH2 by analyzing the phosphorylation status of SlSLAH2. Under aluminum stress conditions, the phosphorylation level of SlSLAH2 in the *Slpp2c72* group was significantly elevated compared to WT (Figure 7f). And the phosphorylation level of SlSLAH2 in the *Slcdpk21* group was significantly lower than WT (Figure 6f). These consistent physiological data demonstrate that SlCDPK21 positively regulates aluminum-induced malate exudation through SlSLAH2 and SlPP2C72 is the key phosphatase responsible for the in vivo dephosphorylation of SlSLAH2 under aluminum stress. We have added these results into the revised manuscript (line 295 to 302 and 333 to 340).

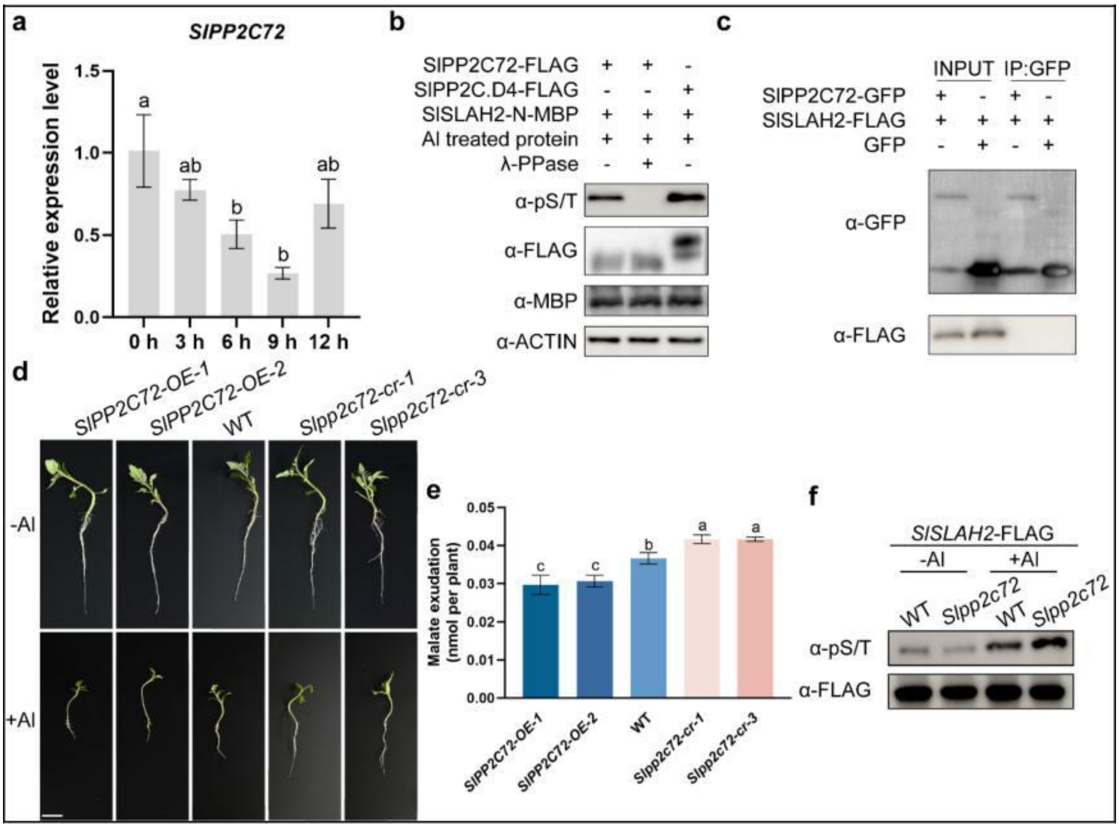

Figure 7. SlSLAH2 was indirectly dephosphorylated by SlPP2C72. (a) The expression level of *SlPP2C72* in WT treated under aluminum stress. (b) SlSLAH2 was dephosphorylated by SlPP2C72 in vitro. (c) Co-IP assay showed SlSLAH2 did not interact with SlPP2C72 in vivo. (d) Root growth of WT, *SlPP2C72* overexpression lines and knockout lines under normal condition and aluminum stress. (e) Malate exudation of different lines. (f) Phosphorylation levels of SlSLAH2 in WT and *Slpp2c72*.

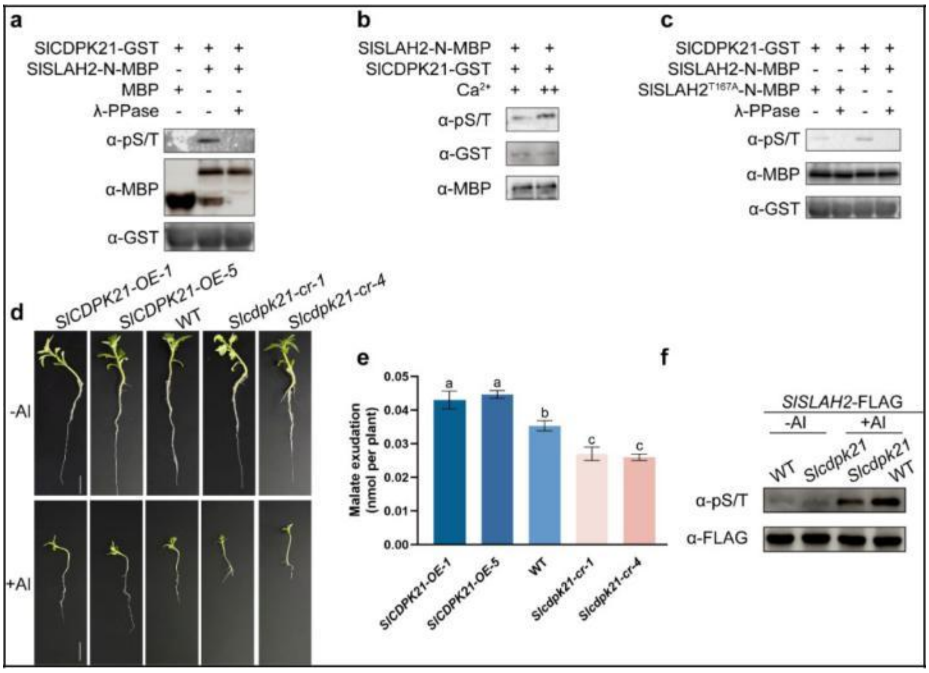

Figure 6. SlCDPK21 enhanced aluminum tolerance in tomato by increasing phosphorylation of SlSLAH2. (a) In vitro kinase assay showed the phosphorylation of SlSLAH2 -N by SlCDPK21. (b) In vitro kinase assay showed the phosphorylation of SlSLAH2 by SlCDPK21 was calcium-dependent. (c) In vitro kinase assay showed T167 of SlSLAH2 was a target of SlCDPK21. (d) Root growth of WT, *SlCDPK2]* overexpression lines and knockout lines under normal condition and aluminum stress. (e) Malate exudation of different lines. (f) Phosphorylation levels of SlSLAH2 in WT and *Slcdpk2]*.

Q4. Figure 7: Why the phosphatase activities of SlPP2C67 also significantly increased in the control treatments (CK, GFP) as shown in Figure 7a and b? Does the measurement system is unstable? For Figure 7g seems to lack an important control that the phosphorylation levels of SlPP2C67 should also be invested in SlCDPK21 mutant plants with or without Al treatments.

A4: We sincerely thank you for raising this key comment. The significantly elevated phosphatase activity of SlPP2C72 in the control treatments is indeed a critical point that lies at the core of the regulatory pathway we have elucidated. We believe the activity of SlPP2C72 is strongly inhibited by aluminum stress signals, and its basal activity is inherently high under non-Al^{3+}-stress conditions. The elevated activity observed in the control groups (CK and GFP) accurately reflects the constitutive, basal activity level of SlPP2C72 in plant cells under homeostasis. This observation carries significant biological meaning. We propose a "brake

release" model to explain its function: Under normal conditions (without aluminum), the "malate exudation brake" is engaged (high SlPP2C72 activity). It continuously dephosphorylates and closes the SlSLAH2 channel, preventing malate efflux and thereby conserving energy and carbon resources for the plant.

As recommended, we assessed the phosphatase activity of SlPP2C72 *in vivo* (Figure 8d). The results were consistent with the in vitro assay: the Al^{3+}-treated *Slcdpk2]* mutants resulted in significantly elevated phosphatase activity of SlPP2C72 compared to the WT. This evidence solidifies the proposed signaling cascade where aluminum stress activates SlCDPK21, which in turn phosphorylates and inhibits SlPP2C72, leading to the sustained activation of SlSLAH2. We have incorporated these results into the revised manuscript.

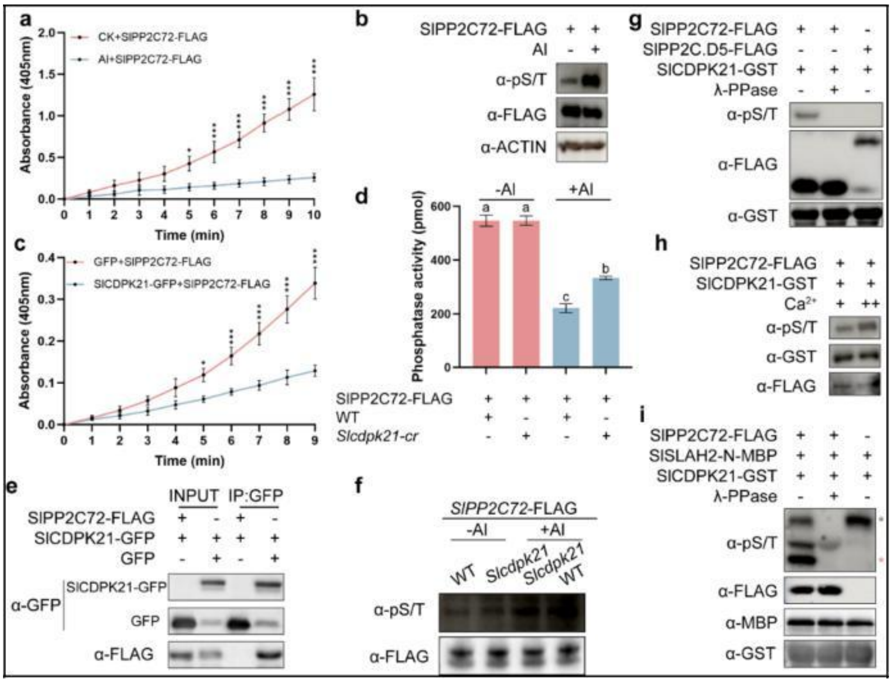

Figure 8. SlCDPK21 phosphorylated SlPP2C72 to ensure SlSLAH2 high phosphorylation state. (a) Effect of aluminum stress on phosphatase activity of SlPP2C72. (b) Phosphorylation of SlPP2C72 induced by 0 or 90 μM $AlCl_3$. (c) Effect of SlCDPK21 on phosphatase activity of SlPP2C72. (d) Effect of SlCDPK21 on phosphatase activity of SlPP2C72. (e) Co-IP assay showed SlCDPK21 interacted with SlPP2C72 in vivo. (f) Phosphorylation levels of SlPP2C72 in WT and *Slcdpk2]*. (g) In vitro kinase assay showed the phosphorylation of SlPP2C72 by SlCDPK21. (i) In vitro kinase assay showed SlCDPK21 simultaneously phosphorylated SlSLAH2-N terminus and SlPP2C72.

Q5. Line 288, phosphorylation should be revised as dephosphorylation.

A5. Thanks for your careful checks. We are sorry for our carelessness. Based on your comments, we have corrected it in the manuscript (line 317).

References:

Cubero-Font, P., Maierhofer, T., Jaslan, J., Rosales, M.A., Espartero, J., Diaz-Rueda, P., Muller, H.M., Hurter, A.L., Al-Rasheid, K.A., Marten, I., Hedrich, R., Colmenero-Flores, J.M., and Geiger, D. (2016). Silent s-type anion channel subunit SLAH1 gates SLAH3 open for chloride root-to-shoot translocation. Curr. Biol. **26,** 2213-2220.

Guo, Y., Shi, Y., Wang, Y., Liu, F., Li, Z., Qi, J., Wang, Y., Zhang, J., Yang, S., Wang, Y., and Gong, Z. (2023). The clade F PP2C phosphatase ZmPP84 negatively regulates drought tolerance by repressing stomatal closure in maize. New Phytol. **237,** 17281744.

Gutermuth, T., Lassig, R., Portes, M.T., Maierhofer, T., Romeis, T., Borst, J.W., Hedrich, R., Feijó, J.A., and Konrad, K.R. (2013). Pollen tube growth regulation by free anions depends on the interaction between the anion channel SLAH3 and calcium-dependent protein kinases CPK2 and CPK20. Plant Cell **25,** 4525-4543.

Hedrich, R., and Geiger, D. (2017). Biology of SLAC1-type anion channels - from nutrient uptake to stomatal closure. New Phytol. **216,** 46-61.

Heng, Y., Wu, C., Long, Y., Luo, S., Ma, J., Chen, J., Liu, J., Zhang, H., Ren, Y., Wang, M., Tan, J., Zhu, S., Wang, J., Lei, C., Zhang, X., Guo, X., Wang, H., Cheng, Z., and Wan, J. (2018). OsALMT7 maintains panicle size and grain yield in rice by mediating malate transport. Plant Cell **30,** 889-906.

Lehmann, J., Jorgensen, M.E., Fratz, S., Muller, H.M., Kusch, J., Scherzer, S., Navarro-Retamal, C., Mayer, D., Bohm, J., Konrad, K.R., Terpitz, U., Dreyer, I., Mueller, T.D., Sauer, M., Hedrich, R., Geiger, D., and Maierhofer, T. (2021). Acidosis-induced activation of anion channel SLAH3 in the flooding-related stress response of Arabidopsis. Curr. Biol. **31,** 3575-3585.e3579.

Xiao, C., Sun, D., Liu, B., Fang, X., Li, P., Jiang, Y., He, M., Li, J., Luan, S., and He, K. (2022). Nitrate transporter NRT1.1 and anion channel SLAH3 form a functional unit to regulate nitrate-dependent alleviation of ammonium toxicity. J. Integr. Plant Biol. **64,** 942-957.

Ye, J., Wang, X., Hu, T.X., Zhang, F.X., Wang, B., Li, C.X., Yang, T.X., Li, H.X., Lu, Y.G., Giovannoni, J.J., Zhang, Y.Y., and Ye, Z.B. (2017). An InDel in the promoter of Al-ACTIVATED MALATE TRANSPORTER 9 selected during tomato domestication determines fruit malate contents and aluminum tolerance. Plant Cell **29,** 2249-2268.

Zheng, X., He, K., Kleist, T., Chen, F., and Luan, S. (2015). Anion channel SLAH3

functions in nitrate-dependent alleviation of ammonium toxicity in Arabidopsis. Plant Cell Environ. **38,** 474-486.

For Reviewer #1:

In this revised manuscript, the authors have adequately addressed my previous concerns, particularly by incorporating electrophysiological assays for SlSLAH2. This study elucidates a novel pathway through which tomato roots secrete malate to alleviate aluminum toxicity. Using the Micro-Tom tomato cultivar, the authors characterized the function of SlSLAH2 in aluminum-induced malate exudation. Based on promoter cis-element analysis and RNA-seq data mining, they demonstrated that SlWRKY37—but not SlSTOP1—regulates SlSLAH2 expression under Al stress. Furthermore, they showed that SlCDPK21 can interact with and phosphorylate SlSLAH2. In addition to phosphorylation, SLAH2 undergoes dephosphorylation to modulate its activity. The study also revealed that SlPP2C72 indirectly mediates the dephosphorylation of SlSLAH2, thereby negatively regulating malate efflux. The following issues remain to be clarified:

Q1: The authors did not describe the method used for malate quantification.

A1: Thank you for your suggestion. We have added the method for malate measurement at the Materials and methods section (line 865-869).

Q2: The evidence supporting the plasma membrane localization of SlSLAH2 remains insufficiently convincing.

A2: Thank you for your suggestion. We expressed SlSLAH2-MYC in *N. benthamiana* leaves, and isolated the plasma membrane from the extracted total protein, to find that SlSLAH2-MYC was expressed in the total protein and membrane, but not in the cytoplasm (Figure R1). This confirms its localization to the plasma membrane.

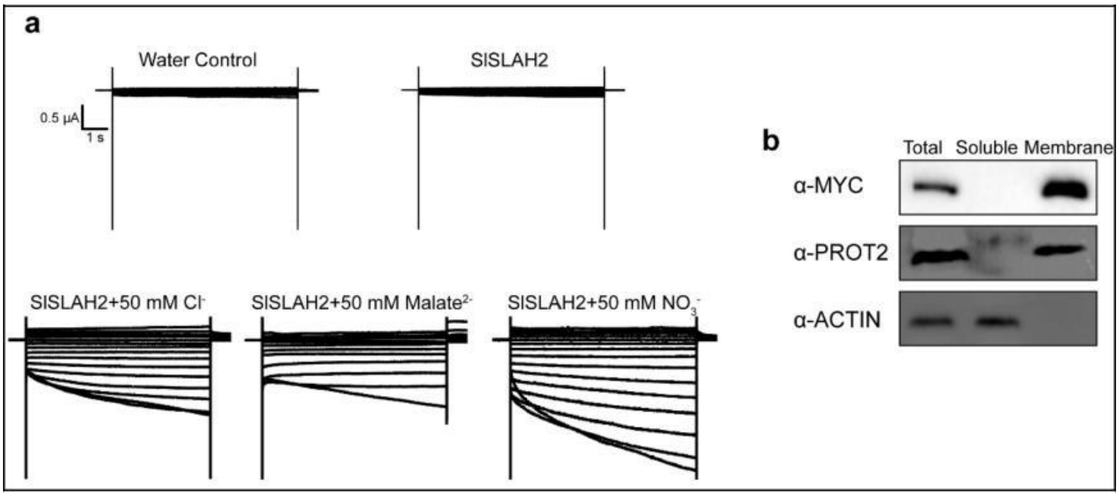

Figure R1. SlSLAH2 was a plasma membrane localized anion transporter. (a) Typical whole-cell current traces were recorded from the *Xenopus* oocytes injected with water perfused with extracellular solution including none anion or from the oocytes injected with SlSLAH2 perfused with extracellular solution including none anion, 50 mM NaCl, 50 mM $NaNO_3$ or 50 mM $C_4H_4O_5Na_2$ (Malate^{2-}). **(b)** Immunoblotting confirmed MYC tagged SlSLAH2 in the plasma membrane protein components extracted from *N. benthamiana* leaves. PROT2, membrane protein marker; ACIN, soluble protein marker. The figure R1 corresponds to Figure S3 in the supplementary materials of the manuscript.

For Reviewer #2:

The revised manuscript shows improvement, with most of my previous questions addressed. However, I still have several concerns:

Q1. The data still lack solid genetic evidence. For instance, the authors identified Thr167 as the phosphorylation site targeted by CDPK21. To conclusively demonstrate that SLAH2 phosphorylation by CDPK21 is necessary for Al-induced malate exudation, complementary experiments expressing the phospho-deficient (SLAH2T167A) and phospho-mimetic (SLAH2T167D) variants in the slah2, cdpk21, pp2c72 mutant backgrounds should be performed. Additionally, does PP2C72 dephosphorylate SLAH2 at the same Thr167 site? **A1-1:** Thank you for your valuable and insightful comment. We completely agree that generating complementation lines expressing the phospho-deficient ($SLAH2^{T167A}$) and phospho-mimetic ($SLAH2^{T167D}$) variants in the *Slslah2*, *Slcdpk21*, and *Slpp2c72* backgrounds would provide crucial genetic evidence.

To directly address your specific question regarding whether Thr167 is the target site for both SlCDPK21-mediated phosphorylation and SlPP2C72-mediated dephosphorylation, we have performed complementary in vitro phosphorylation assays based on your suggestion and the editor's suggestion (Figure R1).

We incubated purified recombinant proteins—SlSLAH2 (wild-type), $SlSLAH2^{T167A}$, and $SlSLAH2^{T167D}$—with root protein extracts from Al^{3+}-treated WT and *Slcdpk21-cr* mutant plants. The phosphorylation level of all three SLAH2 proteins was significantly lower in the *Slcdpk21-cr* extract compared to the WT extract, confirming that SlCDPK21 is the primary kinase responsible for Al^{3+}-induced SlSLAH2 phosphorylation. Importantly, within the *Slcdpk21-cr* background, $SlSLAH2^{T167D}$ exhibited a higher phosphorylation level than $SlSLAH2^{T167A}$. This result is consistent with Thr167 being a major phosphorylation site for SlCDPK21. In parallel assays using root protein extracts from Al^{3+}-treated *Slpp2c72-cr* mutants, we observed that the phosphorylation level of SlSLAH2 was markedly higher than in the WT extract. This provides direct evidence that SlPP2C72 acts as a phosphatase for SlSLAH2. Similar to the results observed in the *Slcdpk21-cr* group, $SlSLAH2^{T167D}$ exhibited a higher phosphorylation level than $SlSLAH2^{T167A}$. While incubation with extracts from the *Slslah2-cr* mutant showed no significant difference in the phosphorylation level of SlSLAH2

proteins compared to the WT. And SlSLAH2^{T167D} also exhibited a higher phosphorylation level than SlSLAH2^{T167A}.

The data indicate that Thr167 is a key regulatory site on SlSLAH2, with SlCDPK21 likely responsible for its phosphorylation and SlPP2C72 for its dephosphorylation. This supports our proposed model wherein the dynamic modification of SlSLAH2 at Thr167 by these enzymes regulates malate exudation.

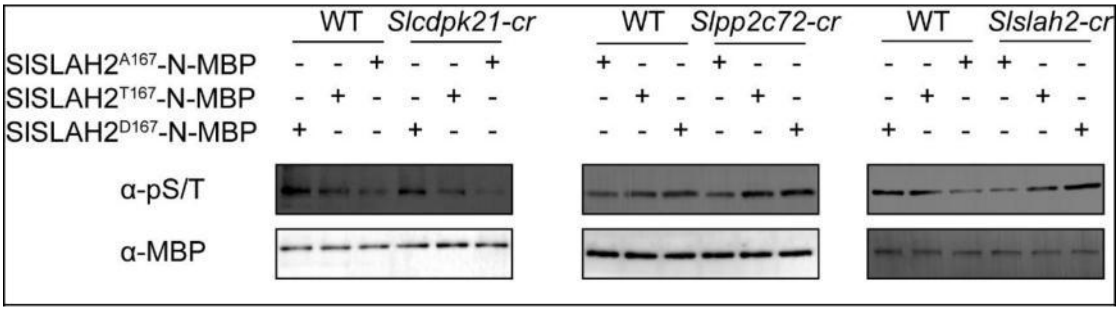

Figure R1. T167 was a target of SlCDPK21 and SlPP2C72. Recombinant SlSLAH2-N-MBP, SlSLAH2^{T167A}-N-MBP (mutant form with the Thr167 replaced with Ala167) and SlSLAH2^{T167D}-N-MBP (mutant form with the Thr167 replaced with Asp167) were incubated Al^{3+} treated WT, *Slcdpk21-cr*, *Slpp2c72-cr*, and *Slslah2-cr* total protein respectively.

A1-2: To determine whether Thr167 is the target site of SlPP2C72, we incubated SlPP2C72-FLAG with either SlSLAH2 or SlSLAH2^{T167A} (Figure R2). The results revealed that mutating Thr167 to Ala167 significantly reduced the phosphorylation level of SlSLAH2, indicating that SlPP2C72 decreases SlSLAH2 phosphorylation through specific dephosphorylation of the Thr167 site.

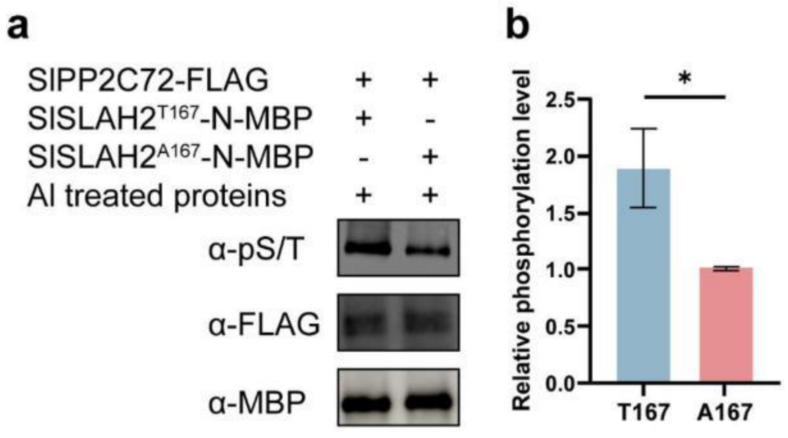

Figure R2. T167 was a target of SlPP2C72. (a) Recombinant SlSLAH2-N-MBP and SlSLAH2^{A167}-N-MBP and 90 jiM $AlCl_3$ treated tomato root extracted proteins were co-incubated. Then SlPP2C72-FLAG protein was added to this system. **(b)** The statistical analysis of signal intensity in (a). The figure R2 corresponds to Figure S18 in the supplementary materials of the manuscript.

Q2. Why do the aluminum treatment concentrations vary across assays measuring root elongation, malate secretion, gene expression, and protein phosphorylation? The root elongation and malate exudation used 30 jiM Al, whereas phosphorylation experiments used 90 jiM Al. Is 30 jiM Al insufficient to regulate protein phosphorylation levels? The concentrations of aluminum treatment should be standardized to ensure the comparability of experimental results.

A2: Thank you for your valuable and insightful comment. Research has shown that even micromolar concentrations of Al^{3+} can cause severe damage to plants. Considering that prolonged exposure to high Al^{3+} concentrations in tomato root cultures may lead to significant harm, potentially resulting in excessive root damage or even death under long-term treatment—which could interfere with observable physiological phenotypes—we selected a relatively low Al^{3+} concentration (30 jiM) for extended-duration experiments. Conversely, to obtain sufficiently strong signal changes within a short timeframe and facilitate clear detection of dynamic phosphorylation level regulation, we opted for a higher Al^{3+} concentration (90 jiM). Additionally, relevant studies on aluminum toxicity in plants revealed that different Al^{3+} concentrations are also selected for various experimental purposes (Figure

R3).

In response to your feedback and based on relevant literature, we have made partial revisions: the Al^{3+} concentration for long-term root growth observation remains 30 jiM (unchanged), while the concentrations for malate exudation (modified) and phosphorylation detection (unchanged) have been standardized to 90 jiM. Accordingly, we have re-evaluated root malate exudation under 90 jiM Al^{3+} concentration. For gene expression analysis, we maintained the Al^{3+} concentration consistent with the RNA-seq treatment at 60 jiM.

Furthermore, to address your query on whether "30 jiM Al is insufficient to regulate protein phosphorylation levels," we conducted supplementary validation experiments to confirm the rationale for selecting 90 jiM (Figure R4): we treated *SlSLAH*2-overexpressing lines with a gradient of Al^{3+} concentrations (0, 10, 30, 60, 90 jiM) and assessed the phosphorylation levels of SlSLAH2. The results clearly demonstrated that SlSLAH2 phosphorylation levels increased significantly with rising Al^{3+} concentrations, reaching their peak at 90 jiM. This dose-dependent response confirms that 90 jiM Al^{3+} effectively and potently induces SlSLAH2 phosphorylation modifications, thereby justifying its selection as the standard treatment concentration for subsequent biochemical assays.

Published Data Figure Redacted

Figure R3. A. Phosphorylation of ALR1 in response to different times of 100 μM Al^{3+} treatment in vivo. **B.** Root growth of genotypes following 7-day control and 1.2 mM Al^{3+} treatments. **C.** Malate secretion from roots following 24 h of control and Al^{3+} (25 μM) treatments. **D.** Expression analysis of Al-responsive genes *ALMT1* in 1-week-old roots of seedlings treated with or without 75 μM Al^{3+} for 6 h (A-D (Xu et al., 2025)). **E.** STOP1-HA levels. WT and *rae6* seedlings were grown on soaked gel medium containing no (−Al) or 0.75 mM Al^{3+} (+Al) for 7 d. **F.** Representative photographs of Al resistance. Seedlings were grown on gel medium soaked with no Al^{3+} or 1.25 mM Al^{3+} for 7 d. **G.** WT and *rae6* seedlings were treated with a 0.5 mM $CaCl_2$ solution containing no Al^{3+} or 20 μM Al^{3+} for 12 h. **H.** The expression of STOP1-regulated genes. Seedlings were grown on gel medium soaked with no Al^{3+} or 0.75 mM Al^{3+} for 7 d (E-H (Wei et al., 2024)). **I.** Six-day-old seedlings of *CPK21p:CPK21-3HA* or *CPK23p:CPK23-3HA* transgenic lines were exposed to either 0 and 20 μM Al^{3+} at pH 5.0 for 0 to 6 h. **J.** Primary root growth. Seedlings were grown in 2% MGRL solution containing 0 and 2 μM Al^{3+} at pH 5.0 for 5 days. **K.** Five-day-old seedlings were treated with 2 ml 2% MGRL nutrient medium with or without 20 μM Al^{3+} for 24 h (I-K (Cao et al., 2025)).

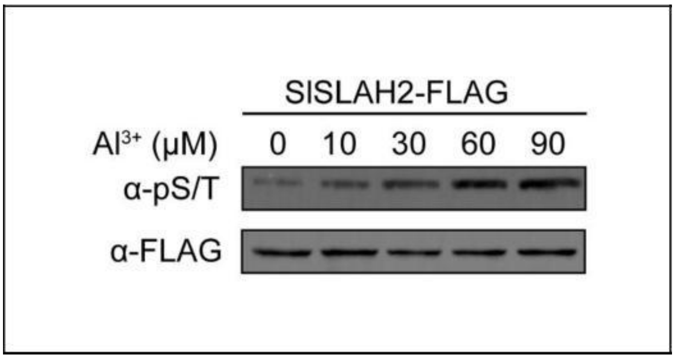

Figure R4. Phosphorylation of the SlSLAH2 under different Al^{3+} concentration. Phosphorylation of the SlSLAH2-FLAG induced by a gradient of Al^{3+} concentrations was detected with the α-Pan Phospho-Ser/Thr antibody (α-pS/T). Total proteins were extracted from *SlSLAH2* overexpression lines treated with increasing concentrations of $AlCl_3$. The figure R4 corresponds to Figure S9 in the supplementary materials of the manuscript.

Q3. The response regarding PP2C phosphatase activity assays remains unsatisfactory. The current figure may mislead readers: the increasing A405 absorbance over time reflects the accumulation of reaction products rather than an increase in enzyme activity. Therefore, I recommend presenting these data as enzyme activity rates (e.g., nmol substrate converted per minute per mg protein) rather than raw A405 values.

A3: Thank you for your valuable and insightful comment. Based on your comment, we have revised the SlPP2C72 phosphatase activity assay as follows: We have re-evaluated SlPP2C72 phosphatase activity using the Serine/Threonine Phosphatase Assay System, which determines the amount of free phosphate released in the phosphatase reaction by measuring the absorbance of the molybdate: malachite green: phosphate complex. Additionally, we calculated SlPP2C72 phosphatase activity as pmol of phosphate produced per minute per mg of protein. The updated data have been included in Figure 8.

Q4. How many biological replicates were performed for the western blot experiments? The blot images should include visible protein markers and the entire membrane. Without full membrane images, it is difficult to assess the specificity and reliability of the results. Since PP2C72 interacts with CDPK21, the authors primarily focus on how CDPK21 phosphorylates PP2C72. However, does PP2C72 also dephosphorylate CDPK21?

A4: Thank you for your suggestion. Our phosphorylation Western blot experiments were performed with three biological replicates, and we have now added quantitative statistical analysis of the phosphorylated protein level in the supplementary materials (Figure S9, S13, S15, S18 and S20). Additionally, the full membrane images, including protein markers, have been uploaded in the source data file.

Regarding whether SlPP2C72 also dephosphorylates SlCDPK21, we conducted in vitro phosphorylation assay (Figure R5). The results showed that SlPP2C72 did not reduce the phosphorylation level of SlCDPK21, indicating that SlPP2C72 does not dephosphorylate SlCDPK21.

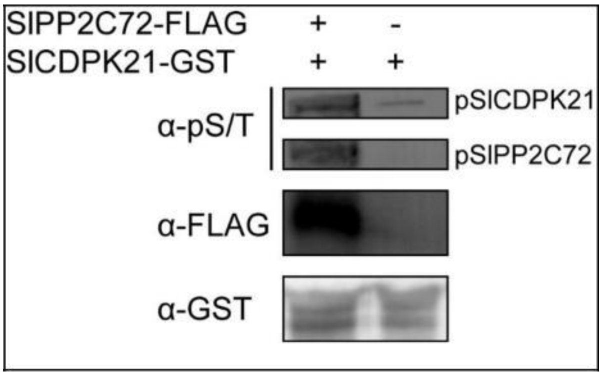

Figure R5. SlPP2C72 does not dephosphorylate SlCDPK21. Recombinant SlCDPK21-GST and purified SlPP2C72-FLAG were co-incubated in protein kinase buffer supplementing ATP.

Q5. The methods section lacks a description of the electrophysiological analysis of Xenopus oocytes.

A5: Thank you for your suggestion. We have added the method for electrophysiological analysis of *Xenopus* oocytes at the Materials and methods section (line 871-880).

Q6. The current title may be misleading, as it suggests that both the kinase and phosphatase promote malate exudation and enhance aluminum tolerance, whereas in reality, they perform opposing functions. Consider revising the title to more accurately reflect their antagonistic roles.

A6: Thank you for your suggestion. We have modified the title into “A Dual Kinase-Phosphatase Circuit Antagonistically Targets SlSLAH2 to Mediate Malate Exudation and

Aluminum Tolerance in Tomato”.

CAO, H. R., et al. (2025) Ca-dependent cytoplasmic and nuclear phosphorylation of STOP1 by CPK21 and CPK23 confers ALMT1-dependent aluminum resistance. *Nat. Commun.,* **16** (1).

WET, X., et al. (2024) H2O2 negatively regulates aluminum resistance via oxidation and degradation of the transcription factor STOP1. *Plant Cell,* **36** (3), 688-708.

XU, C., et al. (2025) The PP2CH-and PBL27-mediated phosphorylation switch of aluminium ion receptor PSKR1/ALR1 controls plant aluminum sensing ability. *Nat. Plants,* **11** (5).

For Reviewer #1:

I am satisfied that my concerns have been fully addressed by the authors. I have no additional comments or questions at this time.

Thank you very much for your positive feedback and for confirming that all your concerns have been satisfactorily addressed. We sincerely appreciate the time, expertise, and constructive suggestions you have provided throughout the review process, which have significantly strengthened the quality and clarity of our manuscript.

Once again, thank you for your valuable contribution to our work.

For Reviewer #2:

The manuscript has been substantially improved and is now suitable for publication.

We are very grateful for your thoughtful evaluation and the encouraging feedback that our revised manuscript is now suitable for publication. Your constructive comments and suggestions throughout the review process have been invaluable in strengthening the scientific rigor and clarity of our work. We sincerely appreciate the time and expertise you have dedicated to helping us improve this study.

Thank you once again for your support and positive assessment.
